# Terminal uranium(V)-nitride hydrogenations involving direct addition or Frustrated Lewis Pair mechanisms

**DOI:** 10.1038/s41467-019-14221-y

**Published:** 2020-01-17

**Authors:** Lucile Chatelain, Elisa Louyriac, Iskander Douair, Erli Lu, Floriana Tuna, Ashley J. Wooles, Benedict M. Gardner, Laurent Maron, Stephen T. Liddle

**Affiliations:** 1 Department of Chemistry, The University of Manchester, Oxford Road, Manchester, M13 9PL UK; 2 LPCNO, CNRS & INSA, Université Paul Sabatier, 135 Avenue de Rangueil, Toulouse, 31077 France; 3 Department of Chemistry and Photon Science Institute, The University of Manchester, Oxford Road, Manchester, M13 9PL UK

**Keywords:** Coordination chemistry, Inorganic chemistry

## Abstract

Despite their importance as mechanistic models for heterogeneous Haber Bosch ammonia synthesis from dinitrogen and dihydrogen, homogeneous molecular terminal metal-nitrides are notoriously unreactive towards dihydrogen, and only a few electron-rich, low-coordinate variants demonstrate any hydrogenolysis chemistry. Here, we report hydrogenolysis of a terminal uranium(V)-nitride under mild conditions even though it is electron-poor and not low-coordinate. Two divergent hydrogenolysis mechanisms are found; direct 1,2-dihydrogen addition across the uranium(V)-nitride then H-atom 1,1-migratory insertion to give a uranium(III)-amide, or with trimesitylborane a Frustrated Lewis Pair (FLP) route that produces a uranium(IV)-amide with sacrificial trimesitylborane radical anion. An isostructural uranium(VI)-nitride is inert to hydrogenolysis, suggesting the 5f^1^ electron of the uranium(V)-nitride is not purely non-bonding. Further FLP reactivity between the uranium(IV)-amide, dihydrogen, and triphenylborane is suggested by the formation of ammonia-triphenylborane. A reactivity cycle for ammonia synthesis is demonstrated, and this work establishes a unique marriage of actinide and FLP chemistries.

## Abstract (web summary)

Despite their importance as mechanistic models for Haber Bosch ammonia synthesis from N_2_ and H_2_, high oxidation state terminal metal-nitrides are notoriously unreactive towards H_2_. Here, the authors report hydrogenolysis of a uranium(V)-nitride, which can occur directly or by Frustrated Lewis Pair chemistry with a borane ancillary.

## Introduction

Terminal metal-nitrides, M≡N, represent a key fundamental class of metal-ligand linkage in coordination chemistry^1^. Although these M≡N triple bonds have been of elementary interest for over 170 years^2^, only in relatively recent times has there been a concerted effort to study their reactivity^1,3^. However, although a variety of reactivity patterns have emerged with metal-nitrides^1^, the vast majority are remarkably unreactive because strong, often highly covalent M≡N triple bonds that result from high oxidation state metal ions—needed to bind to the hard, charge-rich nitride, N^3−^—renders them inherently inert^1,3^. One strategy to increase the reactivity of metal-nitrides is to utilise low oxidation state electron-rich metals to destabilise the M≡N triple bond, but by definition such metals are ill-matched to nitrides and so are difficult to prepare^4^. Additionally, reactivity of metal-nitrides often involves ancillary ligands rather than the M≡N triple bond itself. Overcoming this challenge is difficult because there are very few metal-nitrides where the metal oxidation state or co-ligands can be varied within a homologous family to encourage M≡N triple bond reactivity^1,5^.

Since there is an isoelectronic relationship between the M≡N and N≡N triple bonds of metal-nitrides and dinitrogen, N_2_, respectively, the former are fundamentally mechanistically important with respect to Haber Bosch chemistry where they are invoked as intermediates in the cleavage of the latter and conversion to ammonia, NH_3_, by hydrogenolysis with dihydrogen, H_2_^6,7^. There has thus been intense interest in the reactivity of metal-nitrides with H_2_, and indeed their use in *N*-atom transfer reactivity and catalysis more widely^8–12^, but there are few reports of molecular metal-nitrides reacting with H_2_, and indeed activating H_2_ in this homogeneous context remains a significant challenge in contrast to heterogeneous Haber Bosch chemistry where H_2_-cleavage is essentially barrier-less^6^. One solution to overcome this hydrogenolysis challenge may be to exploit Frustrated Lewis Pair (FLP) chemistry^13,14^, but so far this has been focussed on M–N_2_ complexes^15,16^. Usually with mid- or late-transition metals^3^, most metal-nitride hydrogenations involve sequential protonations^17–22^, but bridging nitrides in poly-iron/-titanium/-zirconium complexes have been reported to react with H_2_ to give imido-hydride and NH_3_ products^23–25^. Only three terminal metal-nitrides have been reported to undergo hydrogenolysis with H_2_. The isostructural *d*^4^ ruthenium(IV)- and osmium(IV)-nitrides [M{N(CH_2_CH_2_PBu^t^_2_)_2_}(N)] (M = Ru, Os) react with H_2_ using the ancillary ligand to shuttle H-atoms to evolve NH_3_^26,27^, and the 5*d*^6^ iridium(III)-nitride [Ir{NC_5_H_3_-2,2′-(C[Me]=N-2,6-Pr^i^_2_C_6_H_3_)_2_}(N)] undergoes concerted reactivity with H_2_ to give [Ir{NC_5_H_3_-2,2′-(C[Me]=N-2,6-Pr^i^_2_C_6_H_3_)_2_}(NH_2_)]^28^. Thus, direct hydrogenolysis of a M≡N triple bond with H_2_ remains exceedingly rare, and involves reasonably electron-rich (≥*d*^4^) metal complexes with low coordination numbers.

As part of our studies investigating actinide-ligand multiple bonding supported by triamidoamine ancillary ligands^29–35^, we have reported two closely related terminal uranium-nitrides [U^V^(Tren^TIPS^)(N)][K(B15C5)_2_] (**1**) and [U^VI^(Tren^TIPS^)(N)] (**2**) [Tren^TIPS^ = N(CH_2_CH_2_NSiPr^i^_3_)_3_^3−^; B15C5 = benzo-15-crown-5 ether]^36–38^ that, unusually^1,5,39^, permit examination of the electronic structure and reactivity of the same isostructural terminal nitride linkage with more than one metal oxidation state. Both react with the small molecules CO, CO_2_, and CS_2_^40,41^, but since only the protonolysis of **1** with H_2_O to give NH_3_ had been previously examined^36^ the ability of **1** and **2** to react with H_2_ has remained an open question. Indeed, the study of molecular uranium-nitride reactivity remains in its infancy^36,37,40–48^, and only very recently the diuranium(IV)-nitride-cesium complex [Cs{U(OSi[OBu^t^]_3_)_3_}_2_(μ-N)] was reported to reversibly react with H_2_ to give the diuranium-imido-hydride complex [Cs{U(OSi[OBu^t^]_3_)_3_}_2_(μ-NH)(μ-H)]^47^. Bridging nitrides tend to be more reactive than terminal ones, so whilst this nitride hydrogenolysis is enabled by the bridging nature of the nitride and polymetallic cooperativity effects^47^, we wondered whether H_2_ activation by **1** or **2** might still be accessible, given prior protonation studies^36^, since this would realise the first terminal f-block-nitride hydrogenolyses. Further motivation to study this fundamental reaction stems from the fact that bridging and terminal uranium-nitride reactivity with H_2_ is implicated in Haber Bosch NH_3_ synthesis when uranium is used as the catalyst^49^, and uranium-nitrides have been proposed as accident tolerant fuels (ATFs) for nuclear fission, but likely reactivity with H_2_ formed from radiolysis under extreme conditions or when stored as spent fuel remains poorly understood.

Here, we report that **2** does not react with H_2_ consistent with a strong U≡N triple bond that is inherently unreactive like many high oxidation state terminal metal-nitrides. However, in contrast **1** reacts with H_2_ under mild conditions despite the fact it can be considered to be a high oxidation state metal and not of a low coordination number nor electron-rich as a 5*f*^1^ metal ion. This hydrogenolysis reactivity is thus unprecedented in molecular metal-nitride chemistry, and further supports the emerging picture that suggests that the 5*f*-electron of **1** should not be considered as purely nonbonding. This study reveals two distinct H_2_-activation mechanisms. When the borane BMes_3_ (Mes = 2,4,6-trimethylphenyl) is present a FLP mechanism operates where two H_2_ heterolysis events and a borane reduction step sequentially combine to furnish a U^IV^-NH_2_ product, and this, to the best of our knowledge, is the first demonstration of the application of bona fide FLP reactivity to actinide chemistry. When the borane is absent, direct 1,2-addition of H_2_ across the U≡N triple bond to give a H−U^V^ = N−H intermediate followed by H-atom migration produces a U^III^-NH_2_ product that is easily oxidised to U^IV^-NH_2_. The direct addition is slower than the FLP-mediated mechanism, demonstrating the facilitating role of FLPs. We find evidence that treating the U^IV^-NH_2_ product with BPh_3_ and H_2_ produces further FLP hydrogenolysis reactivity, since H_3_NBPh_3_ has been detected in reaction mixtures, but this is reversible and produces products that react to give the starting materials. While currently of no practical use this demonstrates further potential for FLPs in this area. We demonstrate an azide to nitride to amide to ammonia reaction cycle, supported by overall hydrogenation involving hydrogenolysis and electrophilic quenching steps.

## Results

### Hydrogenolysis of the terminal uranium(V)-nitride bond

**Fig. 1:**
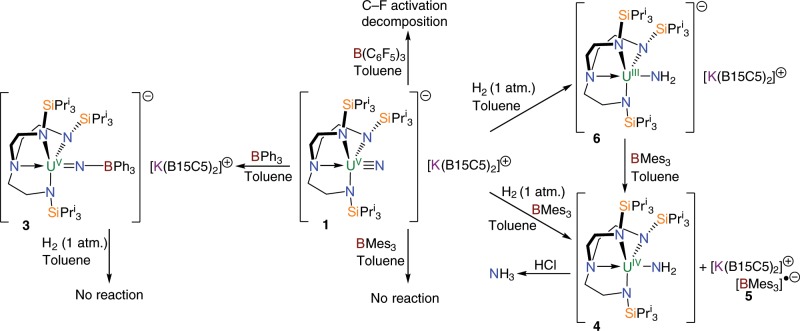
Synthesis of complexes 3–6. Treatment of **1** with the strong Lewis acid B(C_6_F_5_)_3_ results in decomposition, however the milder borane BPh_3_ produces the capped species **3**, which is inert with respect to reaction with H_2_. Complex **1** does not react with the sterically encumbered BMes_3_, but exposure of that mixture to H_2_ produces the amide complex **4** with concomitant formation of the radical anion complex **5**. Addition of H_2_ to **1** produces the amide complex **6**, and subsequent treatment with BMes_3_ produces **4** and **5**. Treating **4** with HCl produces NH_3_. B15C5 = benzo-15-crown-5 ether. Mes = 2,4,6-trimethylphenyl.

Since **2** was found to be unreactive or decomposed to a complex mixture of intractable products when exposed to boranes in the context of this study we examined the reactivity of **1**. With or without H_2_, treatment of **1** in toluene with the strong Lewis acid B(C_6_F_5_)_3_ (BCF) results in decomposition as evidenced by ^19^F NMR spectra of reaction mixtures that show multiple fluorine resonances consonant with multiple C–F activation reactions, Fig. 1. Deleterious C–F bond activation reactivity is well documented for BCF^50^, and so we examined the reaction of **1** with the less Lewis acidic BPh_3_. However, when **1** is treated with BPh_3_ in toluene the adduct complex [U^V^(Tren^TIPS^)(NBPh_3_)][K(B15C5)_2_] (**3**), which when compared to **1** and **2** is perhaps best formulated as a uranium(V)-imido-borate rather than a uranium(V)-nitrido-borane, is rapidly formed quantitatively and isolated in crystalline form in 66% yield, Fig. 1.

**Fig. 2:**
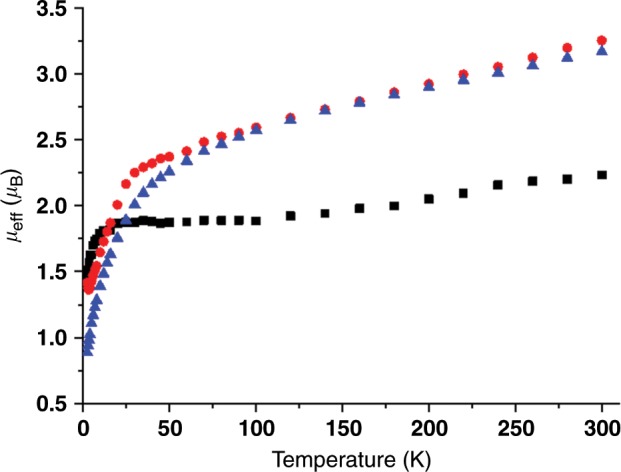
Variable-temperature SQUID magnetic moment data for 3, 6, and 8. Key: **3** (black squares), **6** (red circles), and **8** (blue triangles). Data were measured in an applied magnetic field of 0.1 Tesla.

**Fig. 3:**
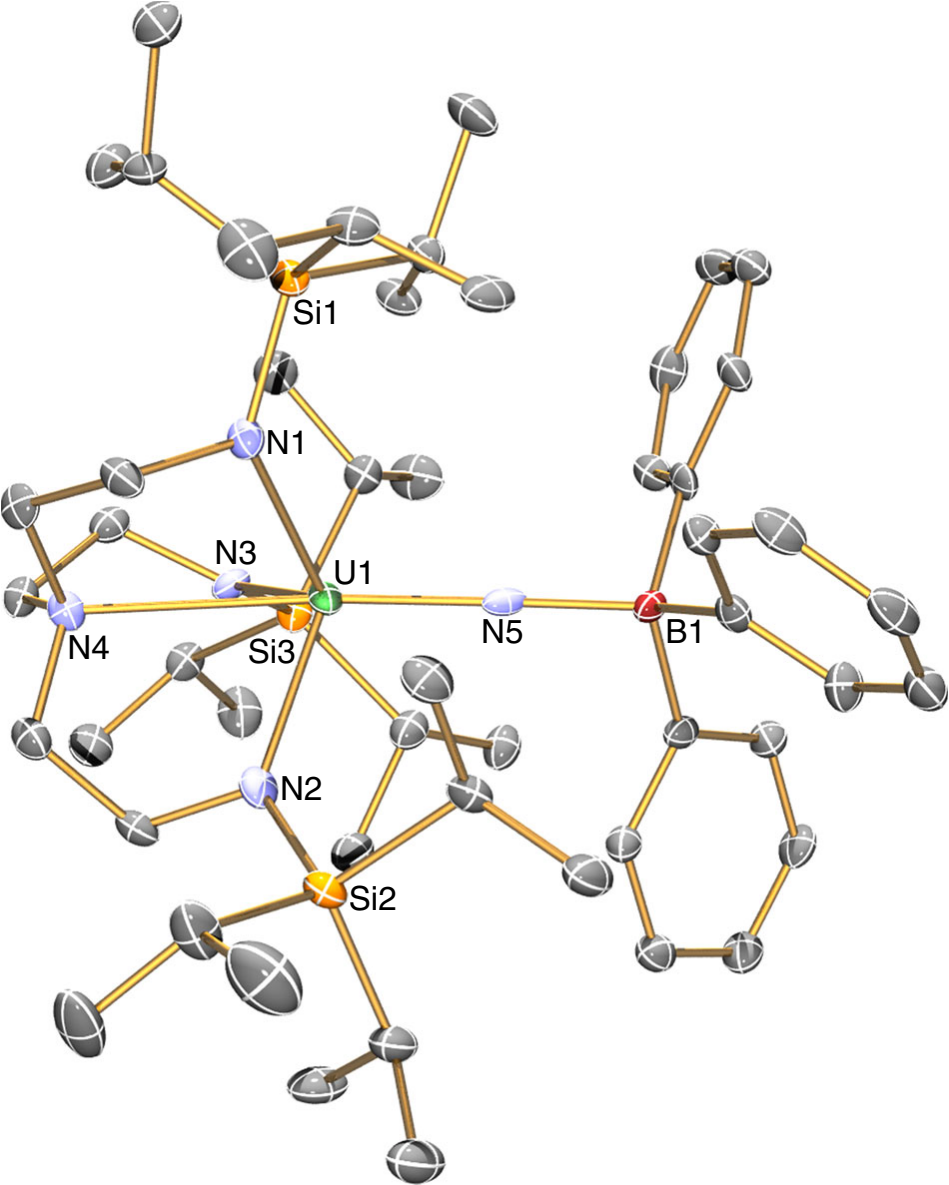
Molecular structure of the anion component of 3 at 150 K and displacement ellipsoids set to 40%. Hydrogen atoms, minor disorder components, lattice solvent, and the [K(B15C5)_2_]^+^ cation component are omitted for clarity. Selected bond lengths (Å): U1-N1, 2.305(5); U1-N2, 2.254(7); U1-N3, 2.312(6); U1-N4, 2.737(5); U1-N5, 1.911(6); B1-N5, 1.581(9).

The retention of uranium(V) in **3** is supported by absorptions in the 5000–12,500 cm^−1^ region of its UV/Vis/NIR spectrum (Supplementary Fig. 1) that are characteristic of intraconfigurational ^2^F_5/2_ to ^2^F_7/2_ transitions of uranium(V)^38,51^, and by variable-temperature SQUID magnetometry, Fig. 2 and Supplementary Fig. 2. A powdered sample of **3** returns a magnetic moment of 2.23 μ_B_ at 300 K (1.96 μ_B_ by solution Evans method) that changes little until 30 K where it falls quickly to a moment of 1.38 μ_B_ at 2 K and this is consistent with the magnetic doublet character of 5*f*^1^ uranium(V)^52–54^. The solid-state structure of **3**, Fig. 3 and Supplementary Fig. 3, reveals a separated ion pair formulation where the nitride has been capped by the BPh_3_ unit. The U–N_imido_ bond length of 1.911(6) Å is consistent with the imido-borate formulation, for example distances of 1.916(4), 1.954(3), and 1.946(13) Å are found in [(Bu^t^ArN)_3_U^V^(NBCF)][NBu^n^_4_] (Ar = 3,5-dimethylphenyl)^55^, [U^V^(Tren^TIPS^)(NSiMe_3_)]^37^, and [U^V^(Tren^TIPS^)(NAd)] (Ad = 1-adamantyl)^37^, respectively, and the B-N_imido_ distance of 1.581(9) Å compares well to the sum of the single bond covalent radii of B and N (1.56 Å)^56^. The U–N_amine_ distance of 2.737(5) Å is long, reflecting the dative nature of the amine donor and that it is *trans* to the strong imido donor, and the U–N_amide_ distances (2.254(7)-2.312(6) Å) are slightly long for such distances^57^, reflecting the formal anionic nature of the uranium component of **3**.

Complex **3** does not react with H_2_ (1 atm.), Fig. 1. Indeed, dissolving a mixture of **1** and BPh_3_ under H_2_ only generates **3**, and so since BPh_3_ has shut all reactivity down by strongly binding to the nitride of **1**, but BCF is too reactive, we examined the use of BMes_3_ (Mes = 2,4,6-trimethylphenyl). In principle, the *ortho*-methyls of the Mes groups of this borane block deactivating strong coordination of Lewis bases to the vacant p-orbital of boron whilst retaining a Lewis acidic boron centre.

To provide a reactivity control experiment, we stirred a 1:1 mixture of **1**:BMes_3_ in toluene under an atmosphere of N_2_ and find no evidence for any adduct formation, Fig. 1, with only free BMes_3_ being observed as evidenced by a resonance at 76.8 ppm in the ^11^B NMR spectrum of the reaction mixture. Repeating this reaction, but under H_2_ (1 atm.), over two days at 298 K results in complete consumption of starting materials with deposition of a dark blue solid. The brown supernatant was removed and found by NMR spectroscopy to contain the known uranium(IV)-amide [U^IV^(Tren^TIPS^)(NH_2_)] (**4**) in 67% yield, Fig. 1, as evidenced by a resonance at 107 ppm in its ^1^H NMR spectrum that corresponds to the amide protons^37^. A control experiment, stirring **1** in toluene over two weeks, also produces **4** from trace, adventitious sources of H^+^, though in far lower proportions, so to prove that the source of H-atoms in **4** originates from H_2_, and not adventitious H^+^^58^, the reaction was repeated under D_2_ (1 atm., 99.8% atom D). Interestingly, whilst [U^IV^(Tren^TIPS^)(ND_2_)] (**4″**, ^2^H δ 107.5 ppm) is formed, confirming that hydrogenolysis by H_2_/D_2_ does indeed occur, it is always accompanied by **4** and [U^IV^(Tren^TIPS^)(NHD)] (**4**′, ^1^H δ 106 ppm, ^2^H δ 106.8 ppm, ^2^*J*_HD_ not resolved). This reveals that H/D exchange occurs over time, so to determine the source of this exchange we studied the reaction of **1** and BMes_3_ with all combinations of H_2_/D_2_ with H_6_-/D_6_-benzene and H_8_-/D_8_-toluene (see Supplementary Methods). We find that when H_2_ is used only **4** is ever detected, but when D_2_ is used **4**, **4**′, and **4″** all form (av. 12, 24, and 64%, respectively) irrespective of whether the solvent is deuterated or not which rules out arene solvents as the H-source. However, when using C_6_H_6_ as solvent for the reaction of **1** with BMes_3_ and D_2_ a weak resonance is observed at −5.2 ppm in the ^2^H NMR spectrum (*cf* −5.35 and −5.87 ppm for *iso*-propyl methine and methyl protons, respectively, in the ^1^H NMR spectrum of **4**). We therefore suggest that the H-source is the Tren^TIPS^ Pr^i^ groups since they have precedent for forming cyclometallates^57^, a reversible amide/imido-cyclometallate + H_2_ equilibrium can be envisaged since it has been previously shown that uranium-Tren-cyclometallates can react reversibly with H_2_/D_2_^59^, and this would also account for the absence of D-scrambling into **4** since Tren^TIPS^ is void of D-atoms.

**Fig. 4:**
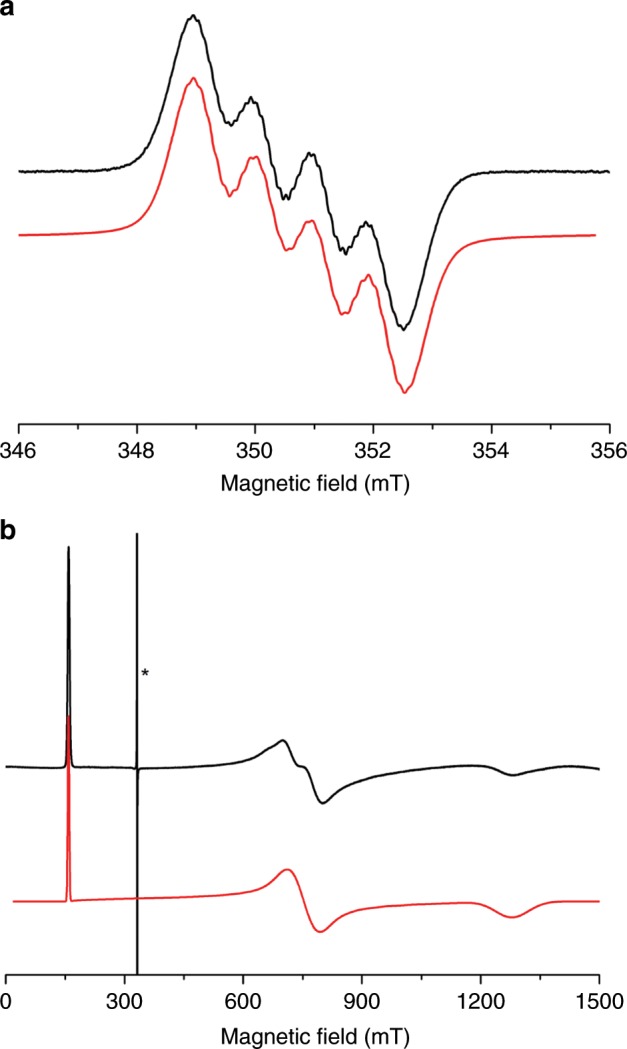
EPR data for 5 and 6. **a** X-band (9.8 GHz) EPR spectrum of **5** at 298 K as a 9 mM solution in THF. The black line is the experimental spectrum and the red line the simulation with *g* = 2.003, *A*(^11^B) = 9.44 G, *A*(^10^B) = 2.7 G, *A*(^1^H) = 1.2–1.4 G. **b** X-band (9.3 GHz) EPR spectrum of a powdered sample of **6** at 20 K. The black line is the experimental spectrum and the red line the simulation with *g* = 4.19, 0.88, and 0.52. The very sharp signal marked with asterisk is a very small quantity of radical impurity with *g* = 2.

The dark blue solid was isolated and after work-up obtained as dark blue crystals, identified as the radical species [K(B15C5)_2_][BMes_3_] (**5**), in 69% yield. This compound has been structurally characterised by single crystal diffraction, see Supplementary Fig. 4. Compound **5** is very similar to [Li(12-crown-4)_2_][BMes_3_]^60^ that contains the same radical anion component, and the EPR data of **5** (*g* = 2.003, *A*(^11^B) = 9.44 G, *A*(^10^B) = 2.7 G, *A*(^1^H) = 1.2–1.4 G), Fig. 4a, confirm the formation of the BMes_3_^•−^ radical anion formulation. The UV/Vis/NIR spectrum of **5** exhibits an intense, (ε = ~8000 M^−1^ cm^−1^) broad absorption centred at ~12,800 cm^−1^, which largely accounts for its dark blue colour, see Supplementary Fig. 5.

Since **1** does not form an adduct with BMes_3_, but the introduction of H_2_ leads to hydrogenolysis to give the amide **4**, we surmised that the **1**/BMes_3_ mixture may constitute a Frustrated Lewis Pair (FLP) system that is evidently capable of activating H_2_, which is confirmed computationally (see below). However, since H_2_ can be a two-electron reducing agent and uranium(V) is normally quite oxidising, we hypothesised that the BMes_3_ may not actually be required. In effect, when **4** is produced it is essentially at the expense of the sacrificial one-electron reduction of BMes_3_ to BMes_3_^•−^, which would formally invoke a uranium(III)-amide precursor that would be nicely in-line with a H_2_-uranium(V) two-electron redox couple. In order to test whether the FLP aspect of this hydrogenolysis chemistry is vital to effecting dihydrogen activation a toluene solution of **1** under H_2_ (1 atm.) was stirred without BMes_3_. Over seven days **1** is consumed with concomitant precipitation of a gray solid identified as the uranium(III)-amide [U^III^(Tren^TIPS^)(NH_2_)][K(B15C5)_2_] (**6**) (45% yield), Fig. 1. The hydrogenolysis reaction is now slower than when BMes_3_ is present, but the reaction is best conducted at 288 and not 298 K, which may also retard the rate of reactivity. When the reaction is alternatively conducted under D_2_ (1 atm.), a mix of **6**, [U^III^(Tren^TIPS^)(NHD)][K(B15C5)_2_] (**6**′) and [U^III^(Tren^TIPS^)(ND_2_)][K(B15C5)_2_] (**6″**) are isolated (66% yield by uranium content) analogously to **4**/**4**′/**4″**, again indicating H/D exchange but confirming the H-atoms of the amide unit in **6** originate from gaseous H_2_. Consistent with these observations, we find that **1** also reacts with 9,10-dihydroanthracene (pKa 31 in DMSO, *cf* 34±4 for H_2_ in DMSO)^61^ to produce an insoluble precipitate and **4** in solution. From this solution we isolated a small crop of red crystals formulated by ^1^H NMR spectroscopy and X-ray diffraction (see Supplementary Fig. 6) as [K(B15C5)_2_][C_14_H_11_]. We suggest that **1** is converted to **6**, and this oxidises to **4** with concomitant reduction of anthracene, ultimately producing [K(B15C5)_2_][C_14_H_11_] via proton abstraction from solvent.

**Fig. 5:**
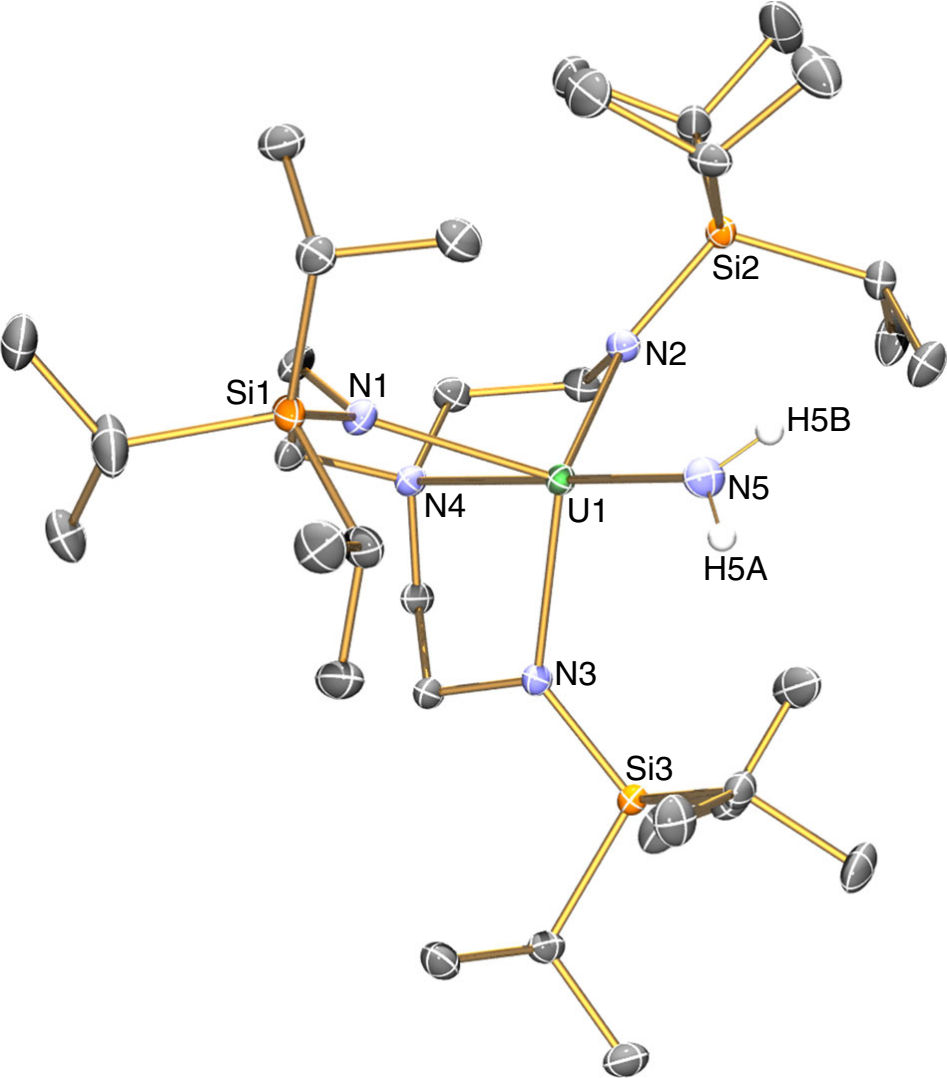
Molecular structure of the anion component of 6 at 150 K and displacement ellipsoids set to 40%. Nonamide hydrogen atoms and the [K(B15C5)_2_]^+^ cation component are omitted for clarity. Selected bond lengths (Å): U1-N1, 2.393(2); U1-N2, 2.404(2); U1-N3, 2.385(2); U1-N4, 2.721(2); U1-N5, 2.335(3).

Unfortunately, **6**/**6**′/**6″** are highly insoluble in non-polar solvents and decompose in polar media so NMR and UV/Vis/NIR data could not be obtained. Complexes **6**/**6**′/**6″**, as their trivalent formulations suggest, are easily oxidised, and the mother liquor from these reactions always contains variable quantities of **4**/**4**′/**4″**, respectively, and heating suspensions of **6**/**6**′/**6″** in C_6_D_6_ in an attempt to obtain ^1^H NMR spectra results in extraction of **4**/**4**′/**4″**, respectively. However, the 5*f*^3^ uranium(III) formulation of **6** is confirmed by variable-temperature SQUID magnetometry, Fig. 2 and Supplementary Fig. 7, where the magnetic moment of **6** is 3.25 μ_B_ at 300 K and this slowly decreases to 2.0 μ_B_ at ~20 K and then falls to 1.59 μ_B_ at 2 K^52–54^. Furthermore, the X-band EPR spectrum of **6** at 20 K, Fig. 4b, exhibits *g* values of 4.19, 0.88, and 0.52, from which a magnetic moment of 2.16 μ_B_ would be predicted that is in good agreement with the observed magnetic moment of **6** at 20 K. The solid-state structure of **6** has been determined, Fig. 5 and Supplementary Fig. 8, revealing a separated ion pair formulation. The salient feature of **6** is the presence of a U^III^-NH_2_ linkage within the uranium component, which has no precedent in uranium(III) chemistry, as evidenced by a U–N_amide_ distance of 2.335(3) Å, which is longer than analogous U^IV^-NH_2_ distances of 2.228(4) Å in **4**^37^, 2.217(4) Å in [U^IV^{η^8^-C_8_H_6_-1,4-(SiPr^i^_3_)_2_}(η^5^-C_5_Me_5_)(NH_2_)]^62^, and 2.183(6) and 2.204(6) Å in [U^IV^{η^5^-C_5_H_2_-1,2,4-(Bu^t^)_3_}(NH_2_)_2_]^63^. The Tren U–N_amine_ and U–N_amide_ distances of 2.721(2) and 2.385(2)-2.393(2) Å, respectively, reflect the anionic uranium(III) formulation of **6**, since, for example, the latter, which are usually quite sensitive to the oxidation state of uranium, are usually ~2.27 Å for uranium(IV) congeners^57^.

In order to experimentally link **6**–**4** we treated **6** with one equivalent of BMes_3_ resulting in immediate reduction of BMes_3_ to give a 1:1 mixture of **4** and **5**, Fig. 1, which is in-line with the reducing nature of **6** as evidenced by ready formation of **4** in supernatant reaction mixtures. These reactions show that although the FLP aspect of the reaction of H_2_ with **1** certainly facilitates and accelerates the hydrogenolysis of the nitride linkage, it is not essential, and the terminal uranium(V)-nitride linkage is reactive enough in its own right to be hydrogenated with H_2_ to give a uranium(III)-amide, and this is confirmed computationally (see below).

### Ammonia synthesis via strong acid

After the hydrogenolysis reactions that produce **4** and **6** we vacuum transferred volatile materials onto hydrochloric acid, but in each case no more than a 5% yield of NH_3_, as its conjugate acid NH_4_^+^, was detected by standard methods. This suggests that although the U^V^≡N-nitride linkage reacts with one equivalent of H_2_ to give U^III/IV^-NH_2_, further reaction of the latter linkages with H_2_ does not occur. Direct treatment of **4**′/**4″** with 0.01 M HCl in THF/Et_2_O, to differentiate the D^+^ as from D_2_ and not D^+^ acid, vacuum transfer onto a 2 M HCl in Et_2_O acid trap, then assay, revealed a mixture of NH_3_D^+^ (^2^D δ 7.12 ppm) and NH_4_^+^ (^1^H δ 7.28 ppm, 1:1:1 triplet, *J*_NH_ = 51 Hz) by ^1^H NMR spectroscopy. Addition of H_2_O results in full D/H exchange to give NH_4_^+^ as the sole ammonium species in 52% yield. Analogously, **6**′/**6**″ produces NH_4_^+^ in 46% yield, and if the HCl acid steps are replaced with analogous DCl reagents then NHD_3_^+^ is first obtained and when this is converted to NH_4_^+^ a similar yield of 48% is obtained showing the internal consistency of this approach, Supplementary Figs. 9, 10. Under the action of strong acid the main by-product is Tren^TIPS^H_3_ from over-protonation, but up to 31% [U^IV^(Tren^TIPS^)(Cl)] (**7**)^36^ could be observed by ^1^H NMR spectroscopy as would be expected from the reaction of **4** with HCl.

### Reversible ammonia-borane formation

**Fig. 6:**
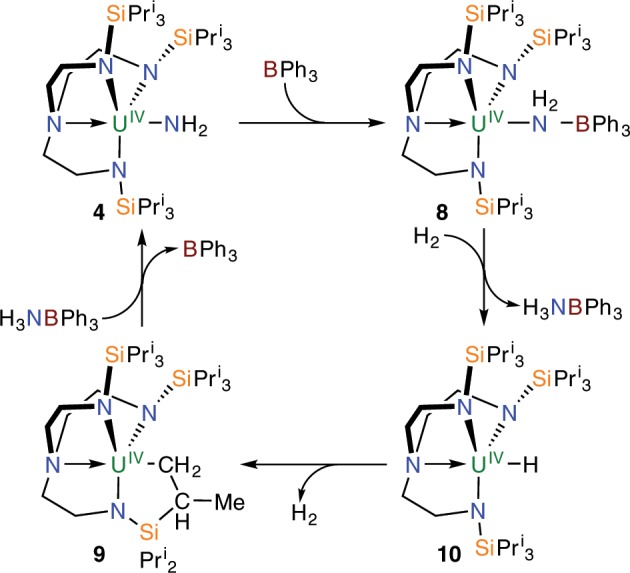
Reactivity of 4. Treatment of **4** with BPh_3_ results in formation of the adduct **8**. Addition of H_2_ to **8** produces the hydride **10** with concomitant elimination of H_3_NBPh_3_. Complex **10** eliminates H_2_ to produce the cyclometallate complex **9**, which in turn can react with H_3_NBPh_3_ to reform **4**.

**Fig. 7:**
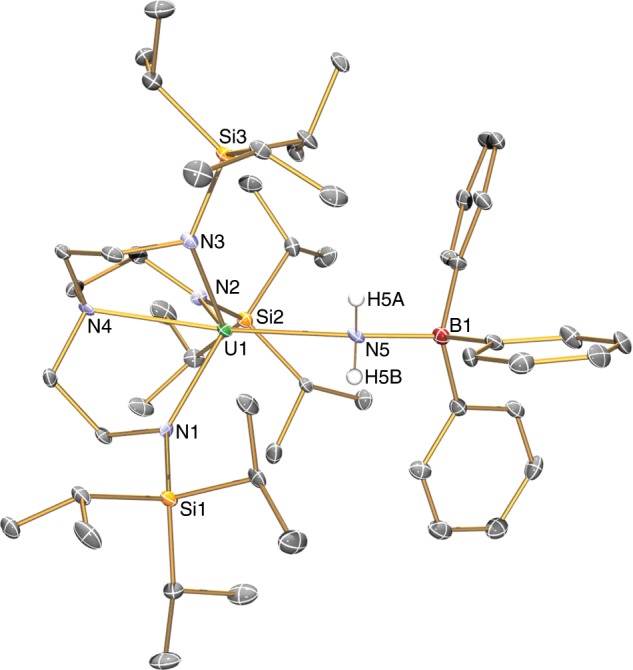
Molecular structure of 8 at 150 K and displacement ellipsoids set to 40%. Nonamide hydrogen atoms and lattice solvent are omitted for clarity. Selected bond lengths (Å): U1-N1, 2.236(5); U1-N2, 2.221(5); U1-N3, 2.246(4); U1-N4, 2.645(5); U1-N5, 2.578(5); B1-N5, 1.637(9).

Since H_2_ does not react with **4** or **6** on their own, we examined whether addition of a borane would facilitate a second hydrogenolysis step; utilising BCF or BMes_3_ with H_2_ results in no reaction and/or formation of unknown, intractable products. We find, however, that **4** reacts with BPh_3_ to form the uranium(IV)-amide-borane adduct [U^IV^(Tren^TIPS^)(NH_2_BPh_3_)] (**8**), Fig. 6, as evidence by its solid-state structure, Fig. 7. The salient feature of the structure of **8** is that although the Tren U–N_amine_ and U–N_amide_ distances of 2.645(5) and 2.221(5)-2.246(5) Å are unexceptional for Tren-uranium(IV) distances^57^, the U–NH_2_ U–N_amide_ distance of 2.578(5) Å is very long^64^, suggesting that coordination of BPh_3_ has severely weakened the U–NH_2_ linkage. However, there is clearly a balance of steric clashing in this region of the molecule since the B-N_amide_ distance of 1.637(9) Å is ~0.06 Å longer than the analogous distance in **3** and ~0.08 Å longer than the sum of the covalent single bond radii of B and N (1.56 Å)^56^. Variable-temperature SQUID magnetometry on a powdered sample of **8**, Fig. 2 and Supplementary Fig. 11, confirms the uranium(IV) formulation of this complex. Specifically, the magnetic moment of **8** at 300 K is 3.17 μ_B_ and this decreases smoothly to a value of 0.89 μ_B_ at 2 K and is tending to zero^52–54^. This is characteristic of uranium(IV) which is a magnetic singlet at low temperature but that exhibits a small contribution from temperature independent paramagnetism to give a nonzero magnetic moment.

The ^11^B NMR spectrum of **8** dissolved in C_6_D_6_ or C_6_D_5_CD_3_ at 293 K exhibits resonances at 67.5 and −3.2 ppm, corresponding to free BPh_3_ and H_3_NBPh_3_, respectively, as confirmed by comparison to authentic samples. The implication, consistent with the long U–N_amide_ and B–N_amide_ distances in **8**, is that **8** is in equilibrium with **4** and free BPh_3_ in solution by B–N bond cleavage, but also that the long U–N_amide_ bond is weakened increasing the basicity of this amide resulting in its rupture, C–H bond activation, and N–H bond formation to concomitantly form H_3_NBPh_3_ and the uranium(IV)-cyclometallate complex [U^IV^{N(CH_2_CH_2_NSiPr^i^_3_)_2_(CH_2_CH_2_NSiPr^i^_2_CH(Me)CH_2_)}] (**9**)^65^. Indeed, trace resonances that match reported data^65^ for **9** could be observed. A variable-temperature ^1^H and ^11^B{^1^H} NMR study (Supplementary Figs. 12, 13) reveals that at 293 K the dominant products are **4** and free BPh_3_, but as the temperature is lowered to 253 and then 233 K resonances attributable to **8** grow in as **4** diminishes such that at 253 K the ratio of **4**:**8** is ~2:1 and at 233 K this ratio is ~2:3. However, when **9** is treated with H_3_NBPh_3_ the formation of **4** and BPh_3_ are observed by ^1^H NMR spectroscopy. This suggests facile, unspecific reversible reactivity but also hints at FLP-type reactivity, so we dissolved a 1:1 mixture of **4** and BPh_3_ under H_2_ (1 atm.), but again find only trace quantities of H_3_NBPh_3_. If **8** reacts with H_2_ to form H_3_NBPh_3_ and [U^IV^(Tren^TIPS^)(H)] (**10**) the latter would be anticipated to eliminate H_2_ to give cyclometallate **9**^59,65^. Indeed, treating [U^IV^(Tren^TIPS^)(THF)][BPh_4_] with NaHBPh_3_ to nominally produce [U^IV^(Tren^TIPS^)(HBPh_3_)] gives H_2_, BPh_3_, and **9** in addition to the anticipated NaBPh_4_ by-product. The reaction cycle in Fig. 6 can thus be proposed where **4** reacts with BPh_3_ and H_2_ to give, possibly via **8**, H_3_NBPh_3_ and **10**, the latter of which extrudes H_2_ to give **9**. Since it is known that **9** reacts with H_3_NBPh_3_ to give **8** and/or **4** and free BPh_3_ then a cycle is most likely established where reactivity is occurring but no discernable products can be isolated since the products react with one another to give the starting materials. Though of little use currently, the formation of H_3_NBPh_3_ suggests that it may be possible to extract out and trap the NH_3_, though so far this system has resisted attempts to do so.

### Closing an ammonia synthesis reaction cycle

**Fig. 8:**
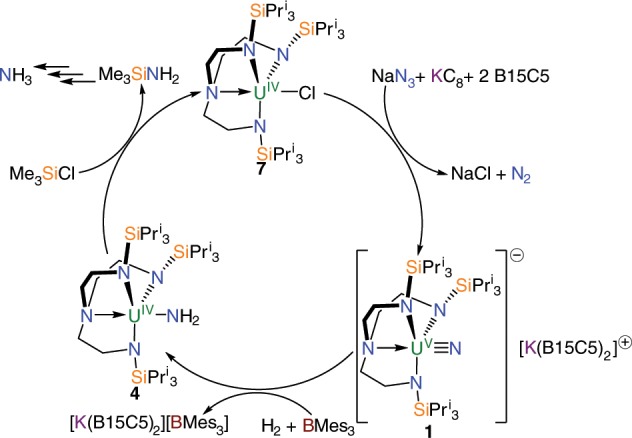
Reaction cycle for the production of ammonia. Treatment of **7** with sodium azide, KC_8_, and two equivalents of B15C5 produces the terminal uranium-nitride **1**, which in turn reacts with H_2_ and BMes_3_ to give **4**. Treatment of **4** with Me_3_SiCl, followed by work-up and acidification steps, as indicated by the multiple arrows, produces ammonia. B15C5 = benzo-15-crown-5 ether. Mes = 2,4,6-trimethylphenyl.

Having effected hydrogenolysis of **1** but found that further reaction with H_2_ either does not occur or seems to occur in a borane-cycle with no discernable products, we sought to close a reaction cycle utilising an electrophile. Accordingly, treatment of **4**, either prepared directly from **1**/H_2_/BMes_3_ or stepwise via **6**, with Me_3_SiCl produces **7** and Me_3_SiNH_2_ that can be quantitatively converted to NH_3_ in the form of ammonium salts. Under nonoptimised conditions an equivalent NH_3_ yield of 53% was achieved. Thus, a reaction cycle for azide to nitride to amide to ammonia by hydrogenation overall is demonstrated at uranium using hydrogenolysis of H_2_ followed by an electrophilic elimination and acid quench, Fig. 8.

### Computational reaction mechanism profiles

**Fig. 9:**
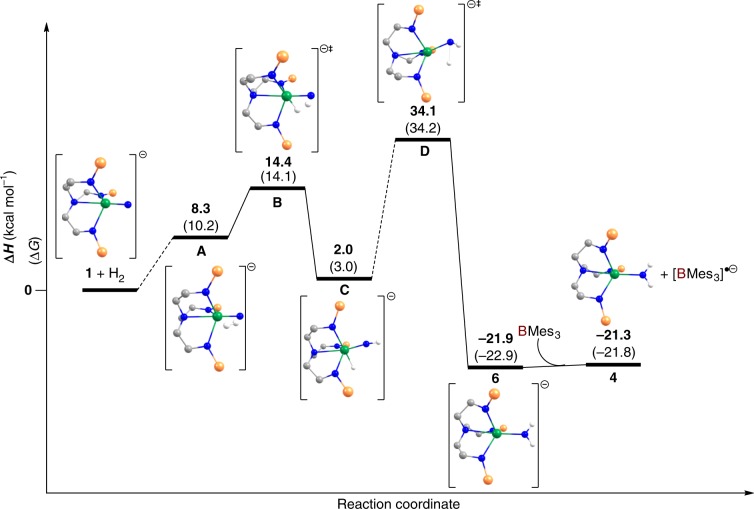
Computed reaction profile for the conversion of 1 to 6 in the absence of BMes_3_ and then conversion to 4 with the addition of BMes_3_. The *iso*-propyl groups of the silyl substituents, carbon-bound hydrogen atoms, and [K(B15C5_2_)]^+^ cation accommodated in the calculations are omitted for clarity. Bold numbers without parentheses refer to ΔH values and numbers in parentheses are ΔG values, both quoted in kcal mol^−1^.

In order to understand the reactions that produce **4**/**5** and **6**, DFT calculations (B3PW91) corrected for dispersion- and solvent-effects were carried out to determine possible reaction pathways for the reaction of complex **1** with H_2_ in the presence or absence of BMes_3_, Supplementary Tables 1–25. We also computed the reaction profile for the hypothetical reaction of **2** with H_2_ (Supplementary Fig. 14), which confirms the experimental situation of no observable reactivity of **2** with H_2_. In the absence of BMes_3_, Fig. 9, H_2_ reacts with **1** in a σ-bond metathesis fashion. The associated barrier is relatively low (14.4 kcal mol^−1^). At the transition state (**B**), the H–H bond is strongly elongated (1.02 Å) and the N–H bond is not yet formed (1.35 Å). The U–N_nitride_ bond is 1.84 Å and the U-H distance is long (2.20 Å). The N–H–H angle is 146.3°, which is quite acute for a metathesis reaction. The NPA charges at the transition state (TS) [U, +1.12; N, −0.84; H, +0.23; H, −0.10] indicate that the TS is better described as a proton transfer. Indeed, inspection of the spin densities of **1**, the H_2_-adduct **A**, and the TS **B** reveal little spin-depletion at N (−0.12 for **1**, −0.13 for **A**, −0.15 for **B**) and that the majority of spin density is at uranium (1.19 for **1**, 1.18 for **A**, and 1.24 for **B**) so *N*-radical character does not appear to play a significant role in the H_2_-activation. Following the intrinsic reaction coordinate yields a uranium(V)-imido-hydride complex (**C**), whose formation is almost athermic (loosely endothermic by 2.0 kcal mol^−1^). Complex **C** can rearrange through a H-atom migration from uranium to parent imido group (transition state **D**), i.e. undergoing a 1,1-migratory insertion, with a reduction of uranium oxidation state at this point from V to III. The associated activation barrier is 32.1 kcal mol^−1^ from **C** (34.1 from the start point). The height of this barrier is due to the need of the hydride to be transferred as a proton to the nucleophilic imido group. However, this barrier is kinetically accessible and in-line with the slow reaction observed experimentally. This TS yields trivalent **6** that is thermodynamically stable (−21.0 kcal mol^−1^). However, in the presence of BMes_3_, complex **6** can be easily oxidised into tetravalent **4** in a process that can be considered to be an essentially athermic electron transfer process since the computed energy difference between **4** and **6** is within the error of the calculation.

**Fig. 10:**
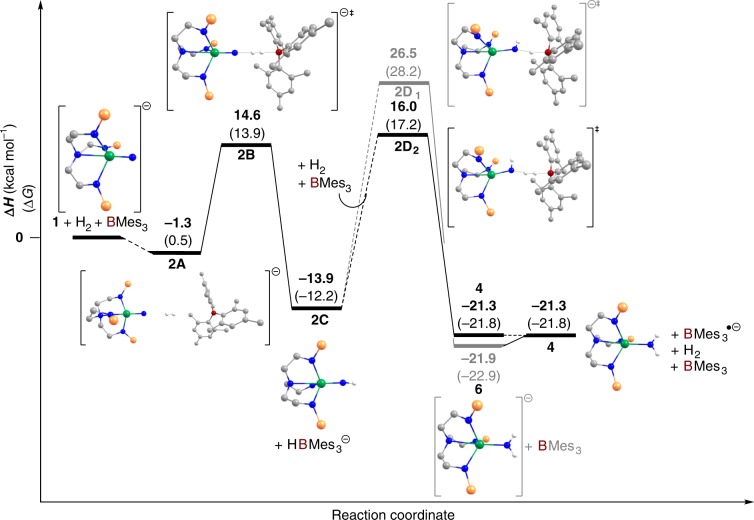
Computed reaction profile for the conversion of 1 to 6 and then 4 in the presence of BMes_3_. The *iso*-propyl groups of the silyl substituents, carbon-bound hydrogen atoms, and [K(B15C5_2_)]^+^ cation accommodated in the calculations are omitted for clarity. Bold numbers without parentheses refer to ΔH values and numbers in parentheses are ΔG values, both quoted in kcal mol^−1^.

In the presence of BMes_3_, Fig. 10, the computed reaction pathway is quite different. After the formation of a loosely bonded H_2_ adduct, the system reaches an H_2_ activation TS, that is reminiscent of FLP complex reactivity. Indeed, at TS **2B**, the H_2_ molecule interacts in a bridging end-on fashion with the nitride (that is the nucleophile of the FLP) and the borane (that is the electrophile). Unlike TS **2B**, the H_2_ molecule is very little activated at **2A** (1.02 vs 0.83 Å, respectively), and neither the N–H bond (1.68 Å) nor the B-H one (1.69 Å) are yet fully formed. The U–N_nitride_ bond distance is similar to that found for **2A** (1.81 Å). The associated barrier is relatively low (14.6 kcal mol^−1^ with respect to the start point) and similar to the σ-bond metathesis mechanism. Therefore, the presence of BMes_3_ does not impact the protonation of the strongly nucleophilic nitride that is very reactive. Again, there is essentially no spin-depletion at the nitride (−0.12 for **1**, −0.13 for **2A**, −0.12 for **2B**) and the unpaired spin density is clearly localised at uranium (1.19 for **1**, 1.20 for **2A**, 1.21 for **2B**), which argues against nitride radical character in this reactivity. The FLP TS **2B** evolves to the formation of a fully dissociative ion pair whose formation is exothermic (−13.9 kcal mol^−1^ from start point). From the uranium(V)-imido complex **2C**, the formation of trivalent **6** then tetravalent **4** was considered. The first, shown by the gray pathway, implies that the hydroborate (HBMes_3_)^−^ delivers the hydrogen to the imido (**2D**_**1**_). However, this route is not favoured because, like the problem in the absence of BMes_3_, the hydride has to be transferred as a proton. The computed barrier of 40.4 kcal mol^−1^ from **2C** (26.5 kcal mol^−1^ from the start point) is in-line with this. The second possibility, shown by the black pathway, involves a second FLP-type activation of H_2_ (**2D**_**2**_). The associated barrier is 10.5 kcal mol^−1^ lower than **2D**_**1**_, demonstrating the beneficial role of BMes_3_. However, the **2D**_**2**_ barrier is also higher than the first FLP barrier, indicating that the uranium(V)-imido complex **2C** is a less strong nucleophile than the uranium(V)-nitride **1**. This also evidenced by the geometry at the TS, where the N–H distance is far shorter than in **2B** (1.44 Å vs. 1.68 Å) inducing a shorter B–H distance (1.54 Å vs. 1.69 Å). The resulting more compact geometry enhances steric repulsion that increases the activation barrier. The **2D**_**2**_ TS yields ultimately tetravalent **4** (via trivalent **6**) whose formation is exothermic by 21.3 kcal mol^−1^.

## Discussion

Despite exhaustive attempts, we find no evidence for any reactivity between the uranium(VI)-nitride **2** and H_2_ irrespective of whether borane promoters are present or not. However, this is not surprising since prior computational studies have suggested that the U≡N triple bond is rather covalent^37^, possibly even more so than group 6 congeners, and so it conforms to the general phenomenon that many metal-nitrides, and especially high oxidation state electron-poor ones, are exceedingly unreactive. To date, CO, CO_2_, and CS_2_ have been found to react with **2**^40,41^, but always much more slowly than **1**, and these small, polar molecules with relatively low-lying π*-orbitals are considerably easier to activate than apolar H_2_ that has only a σ*-orbital available for activation.

In contrast, the reactivity of **1** with H_2_, the mechanisms of which sharply diverge with or without BMes_3_, is surprising and notable because the 5*f*^1^ uranium(V) ion in **1** is high oxidation state and cannot be considered to be electron-rich nor low-coordinate. Indeed, the only example of any molecular uranium-nitride reacting with H_2_ is the diuranium(IV)-nitride-cesium complex [Cs{U(OSi[OBu^t^]_3_)_3_}_2_(μ-N)];^47^ here, the product is the bridging parent imido-hydride complex [Cs{U(OSi[OBu^t^]_3_)_3_}_2_(μ-NH)(μ-H)] and this transformation is enabled by the bridging, polar nature of the nitride and polymetallic cooperativity effects. However, that chemistry stops at the imido-hydride stage, or reverts to nitride and H_2_, and does not proceed to the H-atom 1,1-migratory insertion stage to give an amide. When terminal M≡N triple bonds have been found to react with H_2_ it is with 4*d*^4^ Ru(IV)^26^ or 5*d*^4^ Os(IV)^27^ to give NH_3_ and 5*d*^6^ Ir(III)^28^ to give Ir–NH_2_, since these are the only nitrides that are low-coordinate and sufficiently activated and electron-rich enough to reduce the M≡N triple bond orders by populating anti-bonding interactions. This electron-rich activation is not applicable to **1** being only 5*f*^1^ and that *f*-electron is in principle nonbonding. However, the nitride is a very strong donor ligand that we have previously shown can modulate the m_J_ groundstate of uranium depending how strongly it can donate. Specifically, the nitride forms σ- and π-bonds with the *l* = 0 and *l* = 1 5*f*-orbitals, but also interacts with the *l* = 2 and *l* = 3 5*f*-orbitals where the 5*f*-electron must reside and thus the supposedly nonbonding 5*f*^1^ electron would seem to be not entirely innocent in this circumstance due to an inevitable anti-bonding interaction^38^. Nevertheless, that a metal-nitride of oxidation state as high as +5 and valence electron number as low as one is capable of activating H_2_ without utilising ancillary ligand reactivity and H-atom shuttling is unprecedented^1^. Since the reaction profile calculations do not support the notion of nitride radical character promoting the observed and unexpected reactivities, we suggest that this is due to a combination of the 5*f*-electron in **1** not being wholly nonbonding, and also that the uranium(V)-nitride bond is actually more polar than transition metal analogues.

The reaction profile calculations combined with experimental observations provide an internally consistent account of the reactivity reported here. It is clear from experiment that **1** does not bind BMes_3_, unlike BPh_3_, presumably on steric grounds presenting the potential for FLP chemistry that is intuitively invoked when considering the steric demands of Tren^TIPS^ and BMes_3_. When **1** is reacted with H_2_ in the presence of BMes_3_ the [U(Tren^TIPS^)(N)]^−^ and BMes_3_ components constitute the FLP that can form an encounter complex with H_2_ and the computed intermediate **2A** and TS **2B** are clear evidence for a FLP encounter complex, which facilitates the splitting of H_2_, confirming bona fide FLP reactivity and introducing actinide chemistry to the pantheon of FLP reactivity. Although the conversion of **2C** and (HBMes_3_)^−^ to **6** and BMes_3_ is thermodynamically favourable, it is kinetically the least feasible route to occur due to the inherent barrier of a hydride being a proton source, and instead it appears that a second FLP activation of H_2_ occurs along with oxidation of **6** to give **4**, which is thermodynamically little different to the previous outcome but kinetically more accessible. Within the error of the calculation the oxidation of **6** to **4** is essentially athermic and likely driven by the strongly reducing nature of the uranium(III) ion in **6** coupled to its electron-rich nature. The two-electron reduction on going from **1** to **6** is entirely consistent with the two-electron redox chemistry of H_2_, and indeed the one-electron oxidation of **6** to **4** is simply a sacrificial one-electron reduction of BMes_3_ to BMes_3_^−•^.

The importance of two FLP reaction steps in the conversion of **1** to **6** and then **4** underscores the importance of the facilitating role that FLP chemistry plays in the hydrogenation of **1**. However, more remarkable is that fact that the FLP component is actually not mandatory for hydrogenolysis of the U≡N triple bond to occur, though its absence does slow the reaction significantly demonstrating the facilitating role of the FLP mechanism since the main origin of this impediment is that formally a proton has to evolve from a hydride. In the absence of an FLP mechanism H_2_ undergoes a direct 1,2-addition across the U^V^≡N triple bond to give a H−U^V^=N−H unit that is reminiscent of the aforementioned reactivity of [Cs{U(OSi[OBu^t^]_3_)_3_}_2_(μ-N)]^47^ when their terminal vs bridging natures, respectively, are taken into account. The reactivity of **1** is also reminiscent of aspects of recently reported mechanistic studies of the reaction of uranium(III) with water^66^, and it is germane to note that concerted two-electron redox chemistry at uranium remains a relatively rare phenomena^29,36,67^ with one-electron processes dominating. The 1,2-addition at **1** is effectively H–H heterolysis to generate H^+^ and H^−^, consistent with the polarising nature of the U≡N triple bond. Interestingly, the production of the final U^III^-NH_2_ linkage in **6** from pentavalent **1** by H-atom 1,1-migratory insertion, consistent with the two-electron reducing nature of H_2_ since nucleophilic nitrides tend to react without changing metal oxidation state, is reminiscent of the reactivity of uranium(VI)-nitrides under photolytic conditions, where by a R_3_CH/U^VI^N combination, via a R_3_C•/U^V^=N–H intermediate converts to U^IV^-N(H)CR_3_, since both involve two-electron reductions at uranium overall^37,42,48^. The reactivity of **1** with H_2_ has parallels to the reactivity of the ruthenium(IV)-nitride complex [Ru{N(CH_2_CH_2_PBu^t^_2_)_2_}(N)] with H_2_ to give NH_3_^26^, but with some important differences. The Ru-complex initially reacts with H_2_ across the Ru-N_amide_ not Ru-N_nitride_ bond, so like many nitrides when reactivity occurs it is with the ancillary ligand not the metal-nitride linkage itself as is the case with **1**. However, the Ru-complex does at a later stage transfer a H-atom from Ru to an imido group to form a Ru-NH_2_ group like **C**/**2C**. In contrast, the iridium(III)-nitride complex [Ir{NC_5_H_3_-2,2′-(C[Me] = N-2,6-Pr^i^_2_C_6_H_3_)_2_}(N)] is reported to undergo concerted reactivity with H_2_ to directly afford an amide and no prior coordination of H_2_ to the Ir centre^28^. Looking more widely to sulfido chemistry, the complex [Ti(η^5^-C_5_Me_5_)_2_(S)(NC_5_H_5_)] reacts with H_2_ to give the hydrosulfide-hydride [Ti(η^5^-C_5_Me_5_)_2_(SH)(H)]^68,69^, providing a parallel to the 1,2-addition of H_2_ across the U≡N triple bond of **1**, but unlike **1** the titanocene reactivity halts at the hydrosulfide-hydride formulation and does not undergo a subsequent H-atom 1,1-migratory insertion since that would require formation of SH_2_ and formally the unfavourable reduction of titanium(IV) to titanium(II). So, the reactivity of **1** displays similar and divergent reactivity pathways to known transition metal-nitride reactivity, but combines 1,2-addition and 1,1-migratory insertion steps where transition metals tend to execute either 1,2-additions or 1,1-insertions at the M≡E bond, but are not capable of executing both together.

The reactivity of **4** with BPh_3_ and H_2_ is notable, though complex because it would seem products react to give reactants, because again it invokes the notion of FLP chemistry whereby weakly coordinated [U(Tren^TIPS^)]^+^ and [H_2_NBPh_3_]^−^ components are sufficiently activated to cleave H_2_ to give H_3_NBPh_3_. While this is currently of no practical use it demonstrates the potential for further FLP hydrogenolysis chemistry to convert the parent amide to ammonia. However, we have demonstrated a reaction cycle, where azide is converted to nitride, which undergoes hydrogenolysis to amide, and the amide can be quenched by acid to give ammonia. Thus, overall a nitride has been hydrogenated to ammonia, and the experimentally and computationally supported proposed reactivity mechanisms contribute to our wider understanding of the reactivity of uranium-nitrides toward H_2_ in heterogeneous Haber Bosch and ATF scenarios.

In summary, while the uranium(VI)-nitride **2** is apparently inert with respect to reacting to H_2_, the uranium(V)-nitride **1** is not, suggesting that the 5*f*-electron of the latter is not entirely nonbonding and that the nitride imposes a strong ligand field on uranium. The absence of reactivity for **2** is entirely in-line with the lack of reactivity for high oxidation state metal-nitrides generally, but the latter is not and is notable for being neither low-coordinate nor electron-rich, which are the two requirements previously common to all terminal metal-nitrides that react with H_2_, yet it is reactive. This study reveals two distinct H_2_-activation mechanisms. When the borane BMes_3_ (Mes = 2,4,6-trimethylphenyl) is present a FLP mechanism operates where two H_2_ heterolysis events and a borane reduction step sequentially combine to furnish a U^IV^-NH_2_ product, and this, to the best of our knowledge, is the first demonstration of the application of bona fide FLP reactivity to actinide chemistry. When the borane is absent, direct 1,2-addition of H_2_ across the U≡N triple bond to give a H−U^V^=N−H intermediate followed by H-atom migration produces a U^III^–NH_2_ product that is easily oxidised to U^IV^–NH_2_. The direct hydrogenolysis addition is slower than the FLP-mediated mechanism, demonstrating the facilitating role of FLPs. We find evidence that treating the U^IV^–NH_2_ product with BPh_3_ and H_2_ produces further FLP hydrogenolysis reactivity, since H_3_NBPh_3_ has been detected in reaction mixtures, but this is reversible and produces products that react to give the starting materials. We have demonstrated an azide to nitride to amide to ammonia reaction cycle, supported by overall hydrogenation involving hydrogenolysis and electrophilic quenching steps. Thus, overall a nitride has been converted to ammonia, and the experimentally and computationally supported proposed reactivity mechanisms inform our understanding of the reactivity of uranium-nitrides towards H_2_ in heterogeneous Haber Bosch and ATF scenarios.

## Methods

### General

Experiments were carried out under a dry, oxygen-free dinitrogen atmosphere using Schlenk-line and glove-box techniques. All solvents and reagents were rigorously dried and deoxygenated before use. Compounds were variously characterised by elemental analyses, NMR, FTIR, EPR, and UV/Vis/NIR electronic absorption spectroscopies, single crystal X-ray diffraction studies, Evans and SQUID magnetometry methods, and DFT computational methods.

### Preparation of [U(Tren^TIPS^)(NBPh_3_)][K(B15C5)_2_] (3)

Toluene (20 ml) was added to a stirring mixture of **1** (0.54 g, 0.37 mmol) and BPh_3_ (0.09 g, 0.37 mmol). The resulting mixture was stirred for 16 h to afford a brown precipitate. The mixture was briefly heated to reflux and filtered. Volatiles were removed in vacuo. The resulting brown solid subsequently identified as **3** was washed with pentane (3 × 5 ml) and dried in vacuo. Yield of **3**: 0.42 g, 66%. X-ray quality crystals were grown in benzene solution at room temperature. Anal. calcd for C_79_H_130_BKN_5_O_10_Si_3_U: C, 56.41; H, 7.79; N, 4.16%. Found: C, 56.48; H, 7.82; N, 3.95%. ^1^H NMR (C_6_D_6_, 298 K): *δ* 39.15 (s, 6 H, C*H*_2_), 23.53 (s, 6H, C*H*_2_), 10.69 (s, 6H, Ar-*H*), 9.27 (s, 3H, Ar-*H*), 7.26–6.96 (br m, 14H, Ar-*H*), 4.47–4.20 (m, 32H, OC*H*_2_), −7.92 (s, 54H, Pr^i^-C*H*_3_), −9.12 (s, 9H, Pr^i^-C*H*). ^11^B{^1^H} NMR (C_6_D_6_, 298 K): *δ* 112.8. FTIR: υ/cm^−1^: 2938 (w), 2914 (w), 2859 (m), 1592 (w), 1503 (m), 1454 (m), 1427 (w), 1405 (w), 1382 (w), 1360 (w), 1331 (w), 1288 (w), 1254 (m), 1217 (m), 1125 (s), 1097 (m), 1064 (m), 1050 (m), 1046 (m), 1009 (w), 993 (w), 934 (s), 881 (m), 852 (m), 836 (m), 795 (m), 781 (m), 721 (s), 703 (s), 664 (m), 640 (m), 609 (m), 566 (m), 550 (m), 511 (w), 501 (w), 465 (m), 448 (m). µ_eff_ (Evans method, C_6_D_6_, 298 K): 1.96 µ_B_.

### Attempted reaction of [U(Tren^TIPS^)(N)][K(B15C5)_2_] (1) with H_2_ and BPh_3_

A brown solution of **1** (0.040 g, 0.03 mmol) and BPh_3_ (0.007 g, 0.03 mmol) in C_6_D_6_ (0.5 ml) was degassed and exposed to an atmosphere of H_2_. The brown solution was analysed for 7 days by ^1^H NMR spectroscopy, after which only the formation of **3** was observed.

### Attempted reaction of [U(Tren^TIPS^)(N)][K(B15C5)_2_] (1) with BMes_3_

A colourless solution of BMes_3_ (0.01 g, 0.03 mmol) in C_6_D_6_ (0.5 ml) was added to **1** (0.04 g, 0.03 mmol). The brown solution was analysed ^1^H NMR spectroscopy, revealing resonances of free BMes_3_ and **1**. ^1^H NMR (C_6_D_6_, 298 K): *δ* 38.58 (s, 6H, C*H*_2_), 16.98 (s, 6H, C*H*_2_), 10.03–6.80 (br m, 20H, C*H*_2_, OC*H*_2_, Ar–*H*), 6.72 (s, 6H, Ar-*H*_BMes3_), 3.85 (s, 20H, OC*H*_2_), 2.16 (s, 12H, C*H*_3BMes3_), 2.14 (s, 9H, C*H*_3BMes3_), −5.61 (s, 9H, Pr^i^-C*H*), −6.30 (s, 54H, Pr^i^–C*H*_3_). ^11^B{^1^H} NMR (C_6_D_6_, 298 K): *δ* 76.76.

### Reaction of [U(Tren^TIPS^)(N)][K(B15C5)_2_] (1) with H_2_ and BMes_3_

A brown solution of **1** (0.40 g, 0.28 mmol) and BMes_3_ (0.10 g, 0.72 mmol) in toluene (20 ml) was degassed and exposed to H_2_ (1 atm.). The mixture was stirred for 2 days to ensure the complete consumption of the starting material and **5** started to precipitate as a dark blue solid after 1 day of stirring. The supernatant of **5** was removed by filtration. Dark blue **5** was washed with toluene (3 × 10 ml) and dried in vacuo. Yield of **5**: 0.18 g, 69%. The volatiles of the supernatant were removed in vacuo yielding an oily brown residue containing **4** as the main uranium product. Yield of **4**, based on ^1^H NMR spectroscopy: 67%. ^1^H NMR of **4** (C_6_D_6_, 298 K): *δ* 107 (s, 2H, N*H*_2_), 31.99 (s, 6H, C*H*_2_), 7.92 (s, 6H, C*H*_2_), −5.35 (s, 9H, Pr^i^–C*H*), −5.87 (s, 54H, Pr^i^–C*H*_3_). A similar reaction using D_2_ instead of H_2_ leads to the formation of **4**/**4**′/**4″** (**4**′, ^1^H δ 106 ppm, ^2^H δ 106.8 ppm, ^2^*J*_HD_ not resolved; **4″**, ^1^H δ no resonance in the 100–110 ppm region, ^2^H δ 107.5 ppm). Ammonia liberation after treatment of **4**/**4**′/**4″** with 1 equivalent of HCl led the formation of NH_3_DCl. [B(Mes)_3_][K(B15C5)_2_] (**5**): Anal. calcd for C_55_H_73_KO_10_: C, 69.97; H, 7.79; N, 0%. Found: C, 69.81; H, 7.88; N, 0%. FTIR ν/cm^−1^: 2906 (w), 2873 (w), 1582 (w), 1503 (m), 1452 (m), 1362 (w), 1331 (w), 1291 (w), 1252 (m), 1240 (m), 1219 (m), 1184 (w), 1123 (m), 1099 (m), 1074 (m), 1044 (m), 1005 (m), 936 (m), 852 (m), 840 (m), 813 (w), 777 (w), 748 (m), 734 (m), 695 (w), 675 (w), 603 (w), 573 (w), 562 (w), 542 (w), 509 (w), 467 (m), 454 (w), 411 (w).

### Reaction of [U(Tren^TIPS^)(N)][K(B15C5)_2_] (1) with H_2_ or D_2_

With *H*_*2*_: a brown solution of **1** (1.16 g, 0.81 mmol) in toluene (20 ml) was degassed and exposed to H_2_ (1 atm.). The mixture was stirred for 7 days at 15 °C to ensure the complete consumption of the starting material and formation of **6** as a grey solid. The supernatant of the grey solid was removed by filtration. The solid was washed with toluene (3 × 10 ml) and dried in vacuo. Yield of **6**: 0.53 g, 45%. The volatiles of the filtrate were removed in vacuo yielding an oily brown residue containing traces of **4**, B15C5, and Tren^TIPS^H_3_. With *D*_*2*_: a brown solution of **1** (1.03 g, 0.71 mmol) in toluene (20 ml) was degassed and exposed D_2_ (1 atm.). The mixture was stirred for 7 days at 15 °C to ensure complete consumption of the starting material and formation of **6**/**6**′/**6″** as a grey solid. The supernatant of the grey solid was removed by filtration. The solid was washed with toluene (3 × 10 ml) and dried in vacuo. Yield of **6**/**6**′/**6″**: 0.68 g, 66%. The volatiles of the filtrate were removed in vacuo yielding an oily brown residue containing traces of **4**/**4**′/**4″**, B15C5, and Tren^TIPS^H_3_. X-ray quality crystals of **6** were grown from a 0.069 g/ml solution of **1** in toluene exposed to an atmosphere of H_2_ for three weeks. Anal. calcd for C_61_H_117_KN_5_O_10_Si_3_U: C, 50.81; H, 8.18; N, 4.86%. Found: C, 50.64; H, 8.38; N, 4.95%. FTIR ν/cm^−1^: 2940 (m), 2916 (w), 2881 (w), 2851 (m), 2814 (w), 1596 (w), 1505 (m), 1456 (m), 1411 (w), 1364 (w), 1348 (w), 1333 (w), 1295 (w), 1272 (w), 1254 (m), 1219 (m), 1125 (s), 1107 (m), 1097 (m), 1078 (m), 1046 (m), 1007 (w), 983 (w), 936 (s), 883 (m), 854 (m), 799 (w), 775 (w), 746 (s), 671 (m, for H_2_ only), 664 (m), 654 (w), 622 (m), 603 (w), 591 (w), 560 (w), shoulder 544 (w, for D_2_ only), 536 (w), 530 (w), 505 (m), 467 (w), 458 (w), 440 (m), 424 (w). The insolubility of **6** once isolated precluded the determination of its ^1^H NMR spectrum, the solution magnetic moment by Evans method, and acquisition of a UV/Vis/NIR electronic absorption spectrum. Heating a suspension of **6** in C_6_D_6_ resulted in the observation of resonances that correspond to **4** as evidenced by ^1^H NMR spectroscopy.

### Reaction between [U(Tren^TIPS^)(NH_2_)][K(B15C5)_2_] (6) and BMes_3_

A colourless solution of BMes_3_ (0.01 g, 0.03 mmol) in 0.5 ml of C_6_D_6_ was added to **6** (0.04 g, 0.03 mmol) resulting to the formation of an intense blue solution characteristic of the formation of the radical anion BMes_3_^•−^. Rapidly, dark blue crystals of **5** formed and ^1^H NMR spectrum revealed the formation of **4** in 52% yield.

### Reaction of [UN(Tren^TIPS^)][K(B15C5)_2_] (1) with 9,10-dihydroanthracene

A J Youngs-valve NMR tube was charged with **1** (36 mg, 25 µmol) and 9,10-dihydroanthracene (4.5 mg, 25 µmol). C_6_D_6_ (0.8 ml) was added and the resulting brown mixture was left to stand. After 10 min a turbid red mixture was observed. After standing for 24 h the resulting brown mixture was analysed by ^1^H and ^2^H NMR spectroscopy with the only observable uranium containing product being **4**. During that time a small amount of red crystalline material deposited that was identified as [K(B15C5)_2_][C_14_H_11_] by a combination of X-ray diffraction and ^1^H NMR spectroscopy when redissolved. ^1^H NMR (C_4_D_8_O, 298 K): *δ* 3.59–3.64, 3.67–3.91, 3.88–3.92 (br, m, 32H, OC*H*_2_), 4.41 (s, C = C*H*), 5.62 (td, 2H, 1,9-Anth-C*H*, ^3^*J*_HH_ = 6.85 Hz, ^3^*J*_HH_ = 1.22 Hz), 5.89 (dd, 2H, 4,6-Anth-CH, ^3^*J*_HH_ = 8.31 Hz, ^3^*J*_HH_ = 1.22 Hz), 6.25 (t, ^3^*J*_HH_ = 6.60 Hz, 2,3,7,8-Anth-C*H*), 6.75–6.85 (br, m, 8H, OC*H*C*H*). Resonances for the CH_2_ group were not observed and are likely obscured by residual *d*_8_-THF or crown ether resonances between 3.55 and 3.92 ppm.

### Ammonia formation after addition of 1 equivalent of HCl to [U(Tren^TIPS^)(NHD)] (4′)

Complex **4**′ (0.05 g, 0.06 mmol), formed from the reaction of **1** with D_2_ in the presence of BMes_3_, was treated with 1.2 ml of a 0.05 M HCl solution in THF/Et_2_O (0.06 mmol) and stirred for 2 h at room temperature. All volatiles were then vacuum transferred onto a 2 M HCl solution in Et_2_O (2 ml). Volatiles were removed in vacuo and the resulting white solid was dissolved in 0.6 ml of *d*_6_-DMSO to quantify the amount of ammonia present using ^1^H NMR spectroscopy (quantification using sealed capillary insert of 2,5-dimethylfuran in *d*_6_-DMSO)^59^. Integration of the NH_3_D^+^ multiplet (7.30 ppm) revealed 40% NH_3_DCl. The ^2^H NMR spectrum revealed the presence of a broad resonance at 7.12 ppm. Addition of 10 µl of H_2_O gave complete proton/deuterium exchange, as the resonance at 7.12 ppm in the ^2^H NMR spectrum disappeared and a NH_4_^+^ 1:1:1 triplet (7.28 ppm, *J*_NH_ = 51 Hz) was formed, integration of the triplet revealed 52% NH_4_Cl.

### Ammonia formation after addition of 1 equivalent of HCl to [U(Tren^TIPS^)(NHD)][K(B15C5)_2_] (6′)

Complex **6**′ (0.03 g, 0.021 mmol) was treated with 2.1 ml of a 0.01 M HCl solution in THF/Et_2_O (0.02 mmol) and was stirred for 2 h at room temperature. All the volatiles were then vacuum transferred into a 2 M HCl solution in Et_2_O (2 ml). Volatiles were removed in vacuo and the resulting white solid was dissolved in 0.6 ml of *d*_6_-DMSO to quantify the amount of ammonia present using ^1^H NMR spectroscopy (quantification using sealed capillary insert of 2,5-dimethylfuran in *d*_6_-DMSO)^59^. Analysis of the brown solid residue after distillation of the volatiles revealed the presence of **7** in 1–5% yield with Tren^TIPS^H_3_ as main product. Integration of the NH_3_D^+^ multiplet (7.30 ppm) revealed 35% NH_3_DCl. The ^2^H NMR spectrum revealed the presence of a broad resonance at 7.12 ppm. Addition of 10 µl of H_2_O gave complete proton/deuterium exchange, as the resonance at 7.12 ppm in the ^2^H NMR spectrum disappeared and a NH_4_^+^ 1:1:1 triplet (7.28 ppm, *J*_NH_ = 51 Hz) was formed, integration of the triplet revealed 46% NH_4_Cl.

### Ammonia formation after addition of 1 equivalent of DCl to [U(Tren^TIPS^)(NHD)][K(B15C5)_2_] (6′)

Complex **6**′ (0.04 g, 0.03 mmol) was treated with 2.8 ml of a 0.01 M DCl solution in THF/Et_2_O (0.03 mmol) and stirred for 2 h at room temperature. All volatiles were then vacuum transferred into a 2 M HCl solution in Et_2_O (2 ml). Volatiles were removed in vacuo and the resulting white solid was dissolved in 0.6 ml of *d*_6_-DMSO to quantify the amount of ammonia present using ^1^H NMR spectroscopy (quantification using sealed capillary insert of 2,5-dimethylfuran in *d*_6_-DMSO)^59^. Integration of the NHD_3_^+^ multiplet (7.37 ppm) revealed 11% NHD_3_Cl. The ^2^H NMR spectrum revealed the presence of a broad triplet at 7.24 ppm. Addition of 10 µl of H_2_O gave complete proton/deuterium exchange, as the resonance at 7.24 ppm in the ^2^H NMR spectrum disappeared and a NH_4_^+^ 1:1:1 triplet (7.28 ppm, *J*_NH_ = 51 Hz) was formed, integration of the triplet revealed 48% NH_4_Cl.

### Synthesis of [U(Tren^TIPS^)(NH_2_BPh_3_)] (8)

Toluene (20 ml) was added to a stirring mixture of **4** (0.20 g, 0.23 mmol) and BPh_3_ (0.06 g, 0.23 mmol). The resulting mixture was stirred for a further 16 h to afford a brown precipitate. The mixture was filtered and volatiles were removed in vacuo. X-ray quality crystals grew in the brown oily residue overnight. Crystals were washed with pentane (2 × 5 ml) and dried in vacuo. Yield of **8**: 0.16 g, 62%. Anal. calcd for C_51_H_92_BN_5_Si_3_U: C, 55.26; H, 8.37; N, 6.32%. Found: C, 55.52; H, 8.16; N, 5.91%. NMR spectroscopy reveals that when isolated **8** is dissolved in solution it dissociates to **4** and free BPh_3_ and also trace H_3_NBPh_3_ and **9**, but this equilibrium can be manipulated by cooling samples favouring the formation of **8** so a variable-temperature NMR study was performed, see below. The presence of H_3_NBPh_3_ could not be unequivocally confirmed in the ^1^H NMR spectrum due to its low concentration level in a spectrum dominated by paragmagnetic species, but its presence is confirmed by ^11^B NMR spectroscopy. Trace resonances corresponding to reported data for **9** could be observed^65^. ^11^B{^1^H} NMR (C_6_D_6_, 298 K): *δ* 67.5 (*B*Ph_3_), −3.20 (H_3_N*B*Ph_3_), −55.2 (U-H_2_N*B*Ph_3_). FTIR: υ/cm^−1^: 3293 (w), 3228 (w), 3044 (w), 2938 (m), 2886 (m), 2861 (m), 1590 (w), 1502 (w), 1461 (m), 1428 (m), 1372 (m), 1339 (w), 1316 (w), 1270 (m), 1238 (m), 1166 (w), 1133 (w), 1116 (w), 1047 (m), 1010 (m), 988 (m), 925 (s), 880 (s), 816 (w), 731 (s), 701 (s), 670 (s), 632 (s), 596 (m), 565 (m), 554 (m), 514 (s). µ_eff_ (Evans method, C_6_D_6_, 298 K): 2.96 µ_B_.

### Variable-Temperature NMR study of 8

A brown solution of **4** (0.04 g, 0.05 mmol) in *d*_8_-toluene (0.3 ml) was added to BPh_3_ (0.01 g, 0.05 mmol) in *d*_8_-toluene (0.2 ml). The brownish black solution was analysed by ^1^H and ^11^B{^1^H} NMR spectroscopies at 293, 253, and 233 K. Integrations are listed relatively for functional units within a given species, but note at 293 K **8** is fully dissociated to **4**, at 253 K the ratio of **4**:**8** is ~2:1, and at 233 K that ratio is then ~2:3. ^1^H NMR (C_6_D_5_CD_3_, 293 K): *δ* 107 (s, 2H, N*H*_2_, **4**), 31.99 (s, 6H, C*H*_2_, **4**), 7.92 (s, 6H, C*H*_2_, **4**), 7.4–6.2 (m, br, 15H, B(C_6_*H*_5_)_3_), −5.35 (s, 9H, Pr^i^-C*H*, **4**), −5.87 (s, 54H, Pr^i^-C*H*_3_, **4**). ^11^B{^1^H} NMR (C_6_D_5_CD_3_, 298 K): *δ* 67.5 (*B*Ph_3_), −3.2 (H_3_N*B*Ph_3_). ^1^H NMR (C_6_D_5_CD_3_, 253 K): *δ* 148.1 (s, br, 2H, N*H*_2_, **4**), 43.5 (s, br, 6H, C*H*_2_, **4**), 11.5 (s, vbr, 63H, Pr^i^-C*H* and Pr^i^-C*H*_3_, **8**), 9.2 (s, br, 6H, C*H*_2_, **4**), 7.5–3.7 (s, vbr, 15H, B(C_6_*H*_5_)_3_), −7.5 (s, br, 9H, Pr^i^-C*H*, **4**), −8.5 (s, br, 54H, Pr^i^-C*H*_3_, **4**), −34.5 (s, vbr, 6H, C*H*_2_, **8**), −44.5 (s, vbr, 6H, C*H*_2_, **8**), −154.4 (s, vbr, 1H, N*H*_2_, **8**), −173.6 (s, vbr, 1H, N*H*_2_, **8**). ^11^B{^1^H} NMR (C_6_D_5_CD_3_, 253 K): *δ* 69.0 (*B*Ph_3_), −95.8 (U-H_2_N*B*Ph_3_, **8**). ^1^H NMR (C_6_D_5_CD_3_, 233 K): *δ* 171.9 (s, br, 2H, N*H*_2_, **4**), 50.3 (s, br, 6H, C*H*_2_, **4**), 16.7 (s, br, 9H, Pr^i^-C*H*, **8**), 15.8 (s, br, 6H, C*H*_2_, **4**), 10.04 (s, br, 54H, Pr^i^-C*H*_3_, **8**), 3.7–2.1 (m, br, 15H, H_2_NB(C_6_*H*_5_)_3_) −8.5 (s, br, 9H, Pr^i^-C*H*, **4**), −9.5 (s, br, 54H, Pr^i^-C*H*_3_, **4**), −38.4 (s, br, 6H, C*H*_2_, **8**), −51.2 (s, br, 6H, C*H*_2_, **8**), −159.6 (s, br, 1H, N*H*_2_, **8**), −196.1 (s, br, 1H, N*H*_2_, **8**). ^11^B{^1^H} NMR (C_6_D_5_CD_3_, 233 K): *δ* −107.9 (U-H_2_N*B*Ph_3_, **8**).

### Reaction between [U(Tren^TIPS^)(NH_2_)] (4) and Me_3_SiCl

Me_3_SiCl (6 µl, 0.05 mmol) was added to a brown solution of **4** (0.04 g, 0.05 mmol) in benzene (0.5 ml). The mixture was stirred at room temperature for 2 h. All volatiles containing N-silylated products were distilled under reduced pressure and stirred for 12 h into an aqueous solution of H_2_SO_4_ (0.5 M, 5 ml) to convert the N-silylated products into ammonium salts^70^. After the addition of an excess amount of base (aqueous 30% KOH, 5 ml), ammonia was distilled into HCl solution in Et_2_O (2 M, 2 ml) under reduced pressure. The amount of ammonia was determined by ^1^H NMR spectroscopy using sealed capillary insert of 2,5-dimethylfuran in *d*_6_-DMSO^59^. Yield NH_3_: 53%. To the residual solid fraction containing uranium complexes was added ferrocene as an internal standard in C_6_D_6_ (0.5 ml) to quantify the amount of **7** formed. Yield of **7**: 46%.

## Supplementary information

Supplementary Information

Peer Review File

## Data Availability

The X-ray crystallographic coordinates for the structures of **3**, **5**, **6**, and **8** reported in this study have been deposited at the Cambridge Crystallographic Data Centre (CCDC), under deposition numbers 1870831–1870834 and 1936479. These data can be obtained free of charge from The Cambridge Crystallographic Data Centre via www.ccdc.cam.ac.uk/data_request/cif. ^1^H NMR spectroscopic data for **3**, **4**, and **8** can be found in Supplementary Figs. 15–17. All other data can be obtained from the authors on request.
