# Supplementary material for: Terminal uranium(V)-nitride hydrogenations involving direct addition or Frustrated Lewis Pair mechanisms
Source: Nat Commun. 2020 Jan 17;11:337. doi: 10.1038/s41467-019-14221-y (PMC6969212; doi:10.1038/s41467-019-14221-y)
Supplement: Supplementary file 1 — Supplementary Information [file 41467_2019_14221_MOESM1_ESM.pdf]

Terminal Uranium(V)-Nitride Hydrogenations Involving Direct Addition or Frustrated Lewis Pair Mechanisms

Chatelain et al.

Supplementary Figures

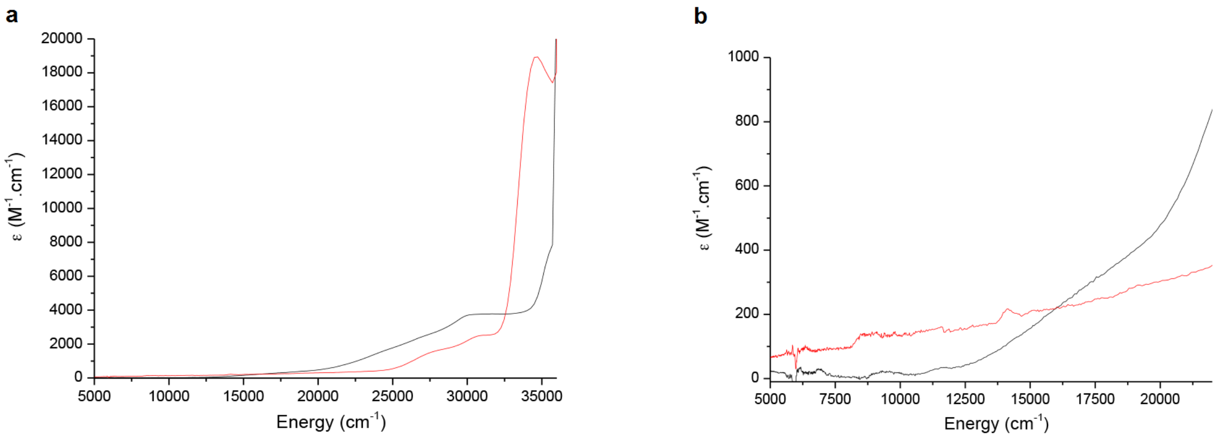

Supplementary Figure 1. a UV/Vis/NIR optical absorption spectra of toluene solutions of [U(NBPh$_3$)(Tren^{TIPS})][K(B15C5)$_2$] (**3**, black trace) and [U(NH$_2$BPh$_3$)(Tren^{TIPS})] (**8**, red trace). **b** zoom-in of NIR region for **3** (black trace) and **8** (red trace).

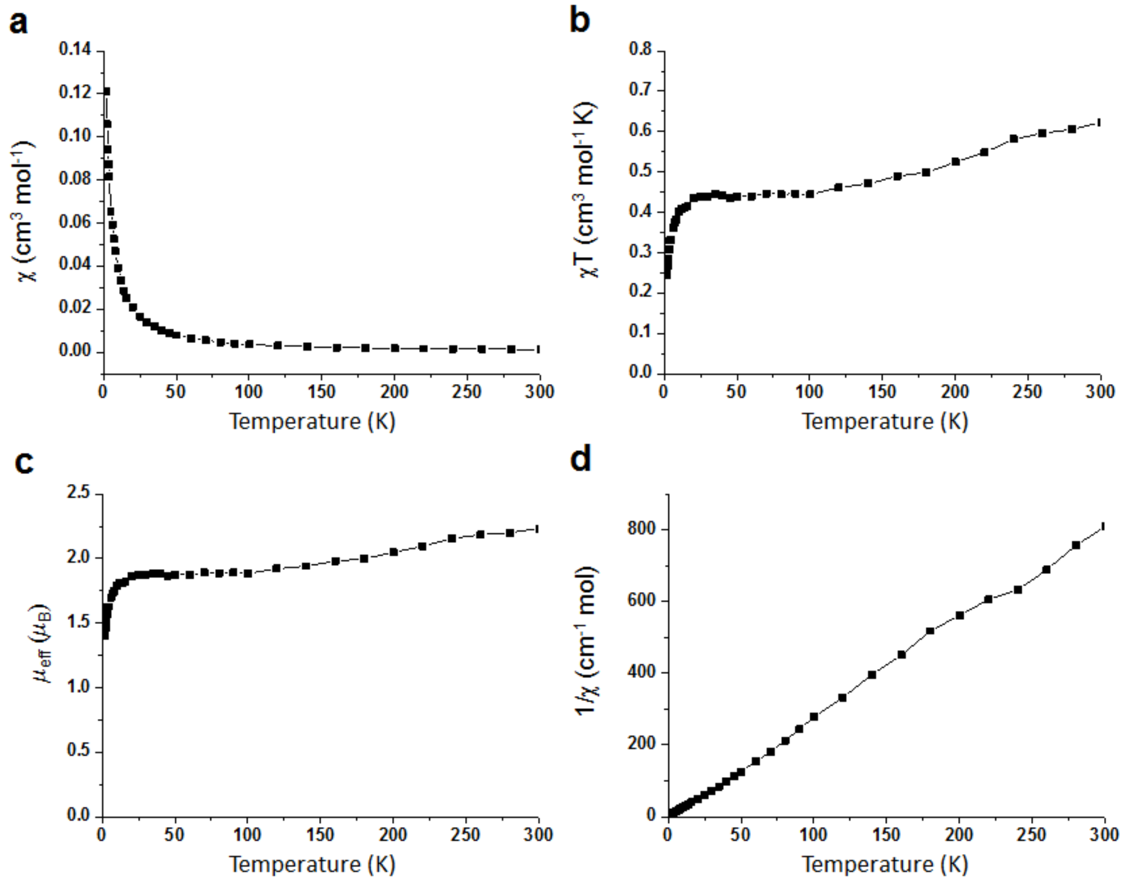

Supplementary Figure 2. Variable-temperature SQUID magnetic data for [U(NBPh$_3$)(Tren^{TIPS})][K(B15C5)$_2$] (**3**) measured at 0.5 T. **a** χ vs T. **b** χT vs T. **c** μ_{eff} vs T. **d** χ^{-1} vs T. Magnetic data are shown per uranium ion. The solid lines are a guide to the eye only.

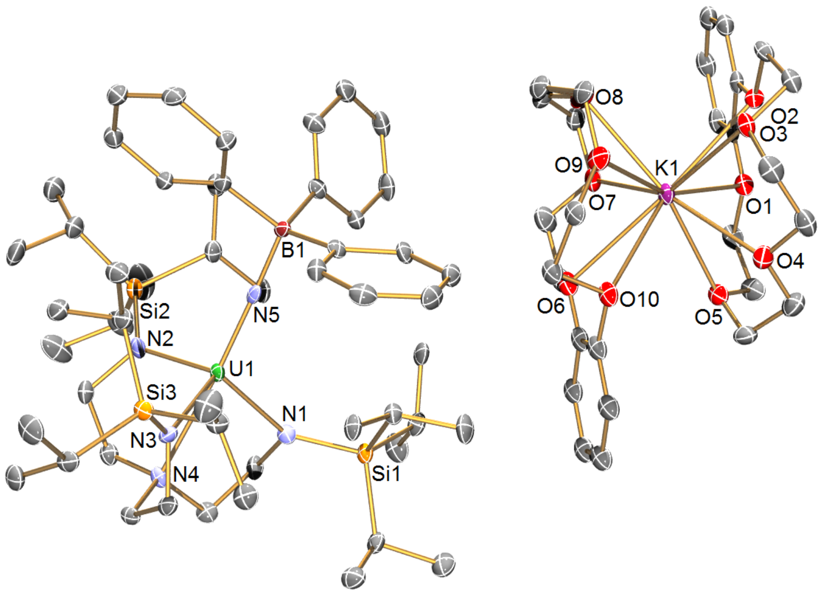

Supplementary Figure 3. Molecular structure of [U(NBPh$_3$)(Tren^{TIPS})][K(B15C5)$_2$] (**3**) at 150 K and displacement ellipsoids set to 40%. Hydrogen atoms, minor disorder components, and lattice solvent are omitted for clarity.

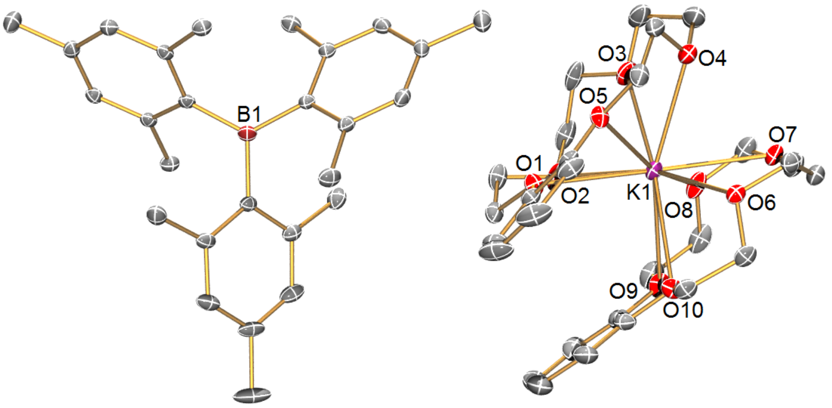

Supplementary Figure 4. Molecular structure of [K(B15C5)$_2$][BMes$_3$] (**5**) at 150 K and displacement ellipsoids set to 40%. Hydrogen atoms and lattice solvent are omitted for clarity.

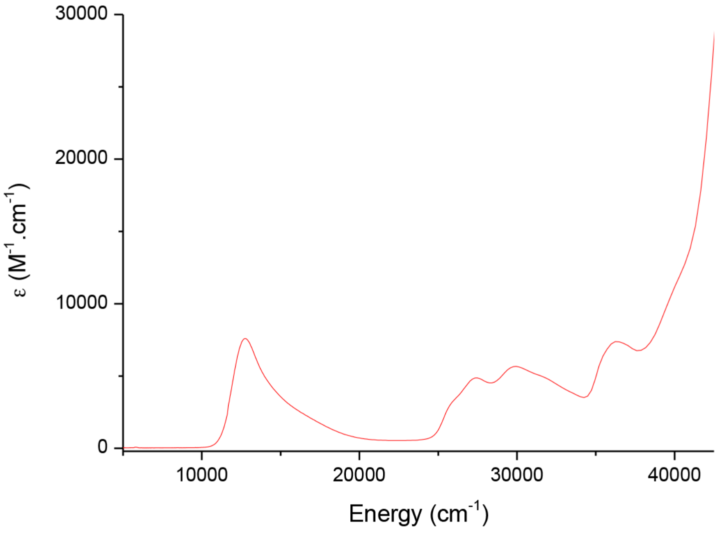

Supplementary Figure 5. UV/Vis/NIR optical absorption spectrum of [K(B15C5)$_2$][BMes$_3$] (**5**, red trace) in THF.

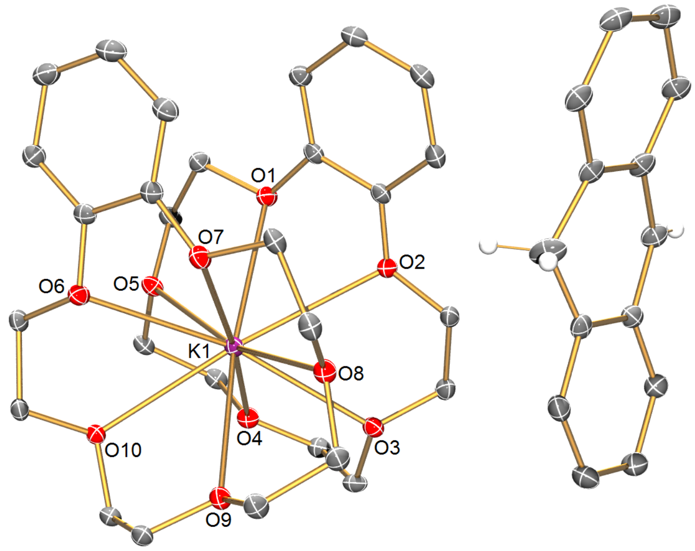

Supplementary Figure 6. Molecular structure of [K(B15C5)$_2$][C$_{14}$H$_{11}$] at 100 K and displacement ellipsoids set to 40%. Hydrogen atoms (apart from the 9,10-positions of the anthracenide anion which are disordered; only one component is shown) and lattice solvent are omitted for clarity.

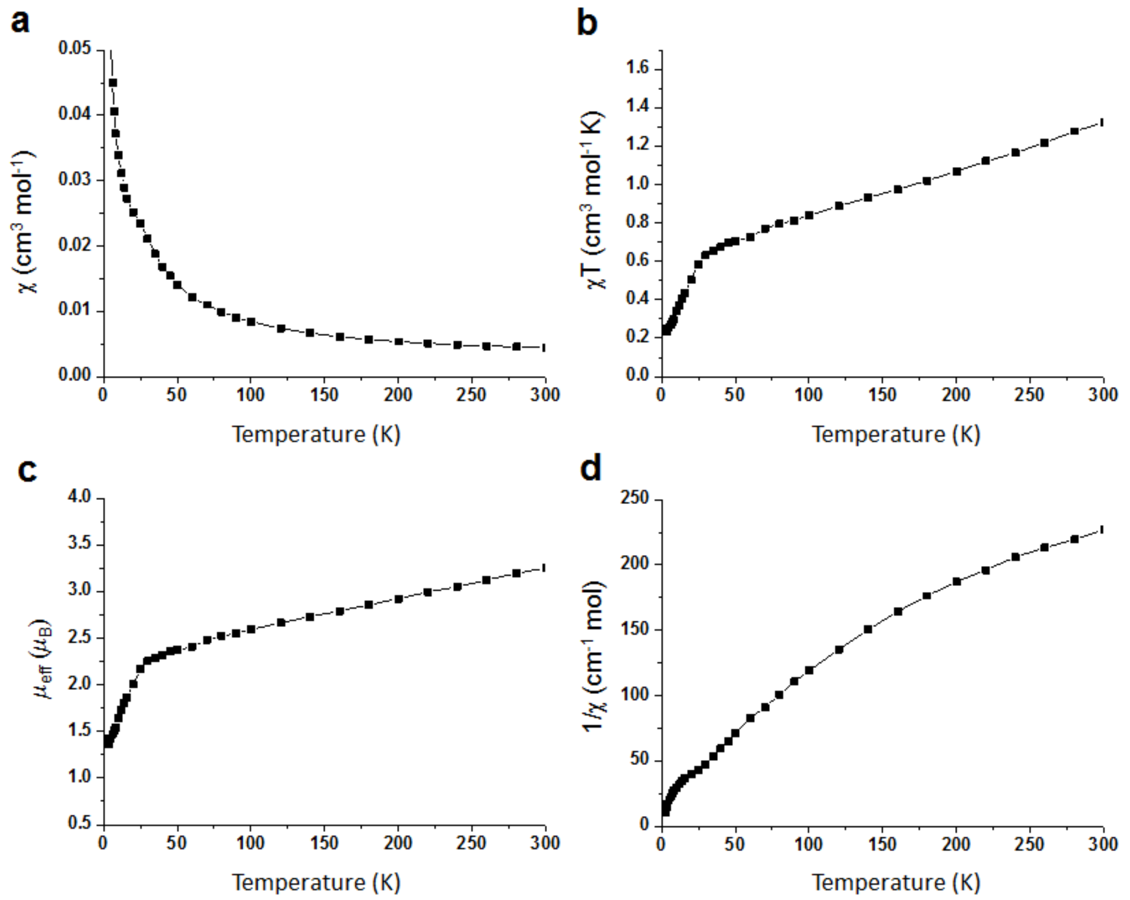

Supplementary Figure 7. Variable-temperature SQUID magnetic data for $[U(Tren^{TIPS})(NH_2)][K(B15C5)_2]$ (**6**) measured at 0.1 T. **a** χ vs T. **b** χT vs T. **c** μ_{eff} vs T. **d** χ^{-1} vs T. Magnetic data are shown per uranium ion. The solid lines are a guide to the eye only.

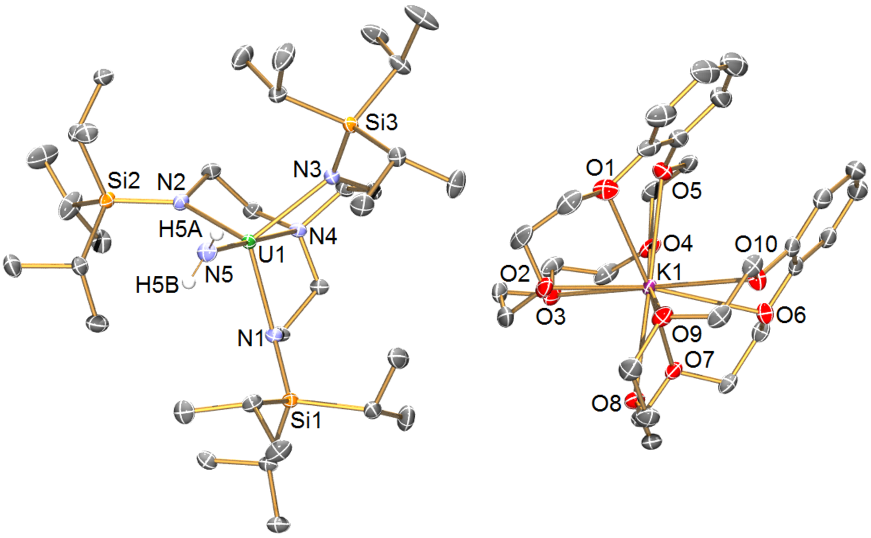

Supplementary Figure 8. Molecular structure of $[U(Tren^{TIPS})(NH_2)][K(B15C5)_2]$ (**6**) at 150 K and displacement ellipsoids set to 40%. Non-amide hydrogen atoms are omitted for clarity.

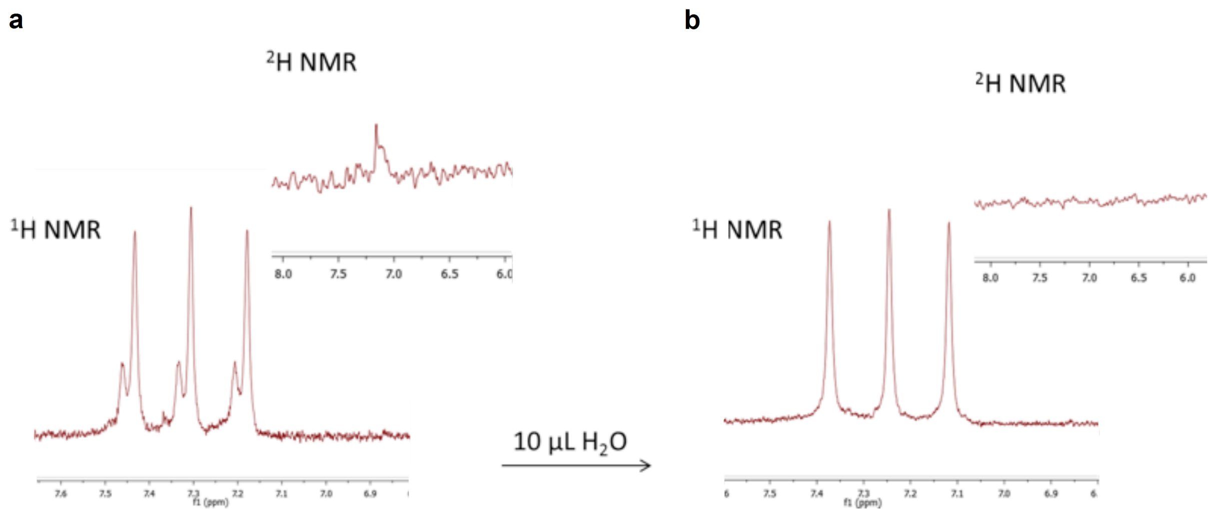

Supplementary Figure 9. NMR spectra of the ammonia quantifications from **6'** showing the D-incorporation when HCl was used. **a** ^{1}H and ^{2}H NMR spectra before treatment with H_2O. **b** ^{1}H and ^{2}H NMR spectra after treatment with H_2O.

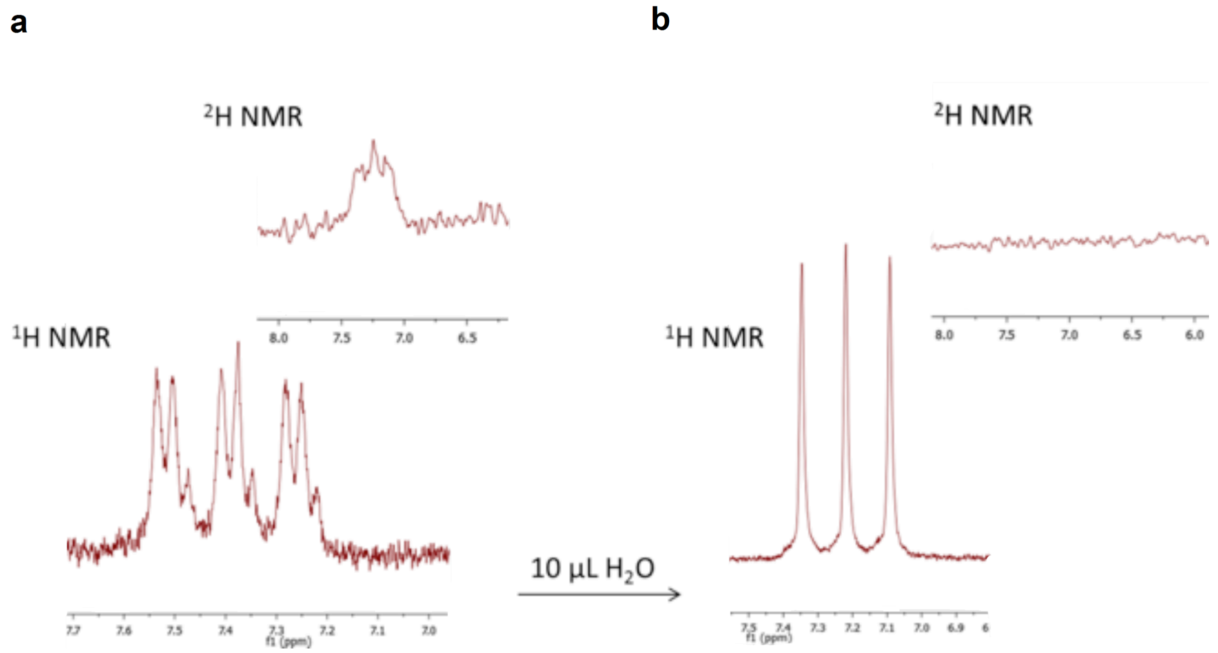

Supplementary Figure 10. ^{1}H and ^{2}H NMR spectra of the ammonia quantifications from **6'** showing the H-incorporation when DCl was used. **a** ^{1}H and ^{2}H NMR spectra before treatment with H_2O. **b** ^{1}H and ^{2}H NMR spectra after treatment with H_2O.

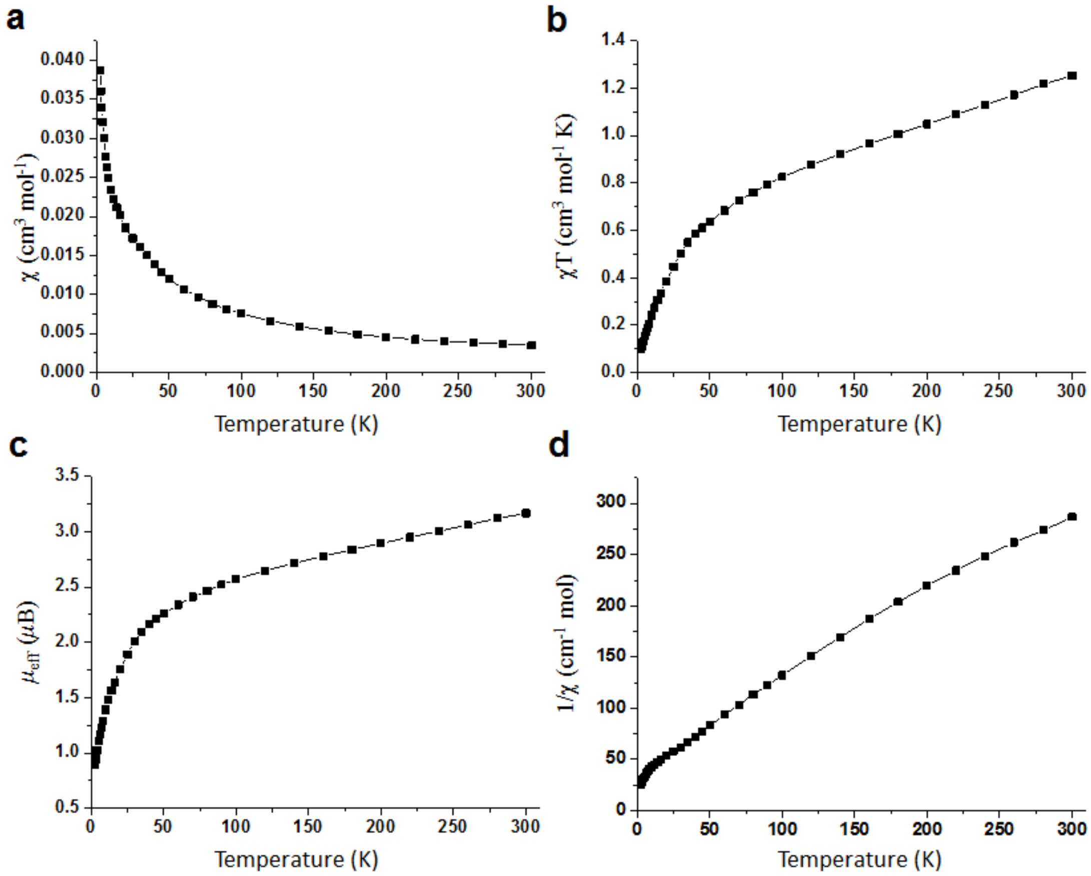

Supplementary Figure 11. Variable-temperature SQUID magnetic data for [U(Tren^{TIPS})(NH_2BPh_3)] (**8**) measured at 0.1 T. **a** χ vs T. **b** χT vs T. **c** μ_{eff} vs T. **d** χ^{-1} vs T. Magnetic data are shown per uranium ion. The solid lines are a guide to the eye only.

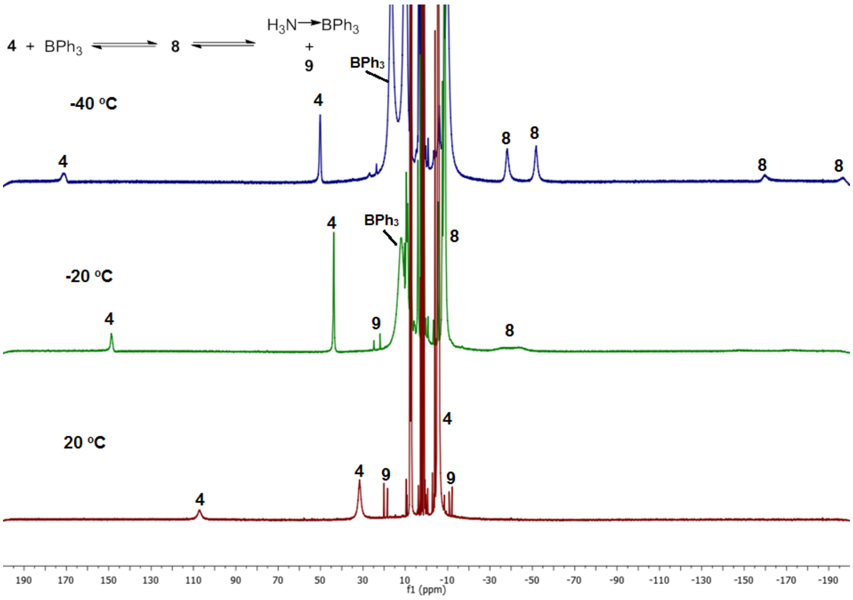

Supplementary Figure 12. Variable-temperature ^{1}H NMR spectra of BPh_3 added to an equimolar quantity of **4** dissolved in $C_6D_5CD_3$ and measured at 293, 253, and 233 K.

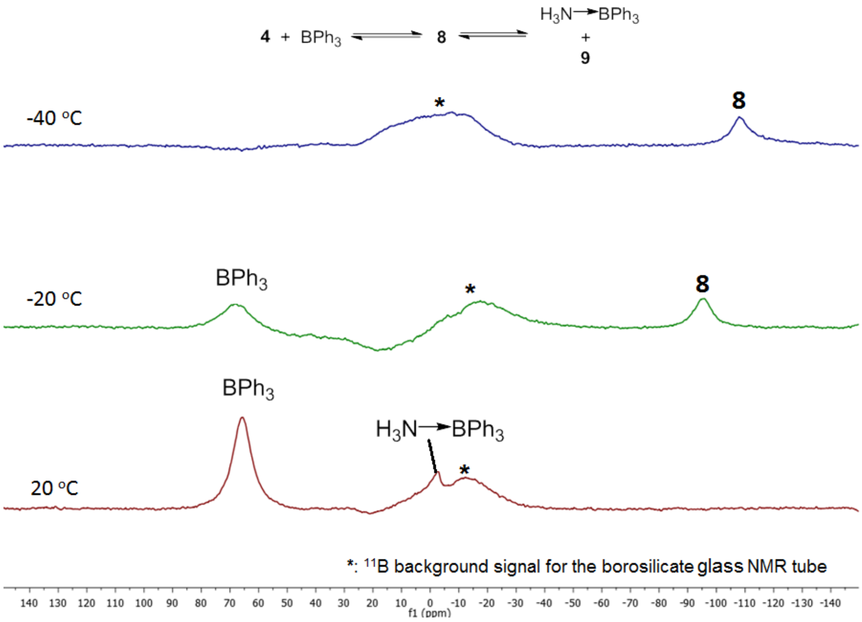

Supplementary Figure 13. Variable-temperature $^{11}B\{^{1}H\}$ NMR spectra of BPh_3 added to an equimolar quantity of **4** dissolved in $C_6D_5CD_3$ and measured at 293, 253, and 233 K.

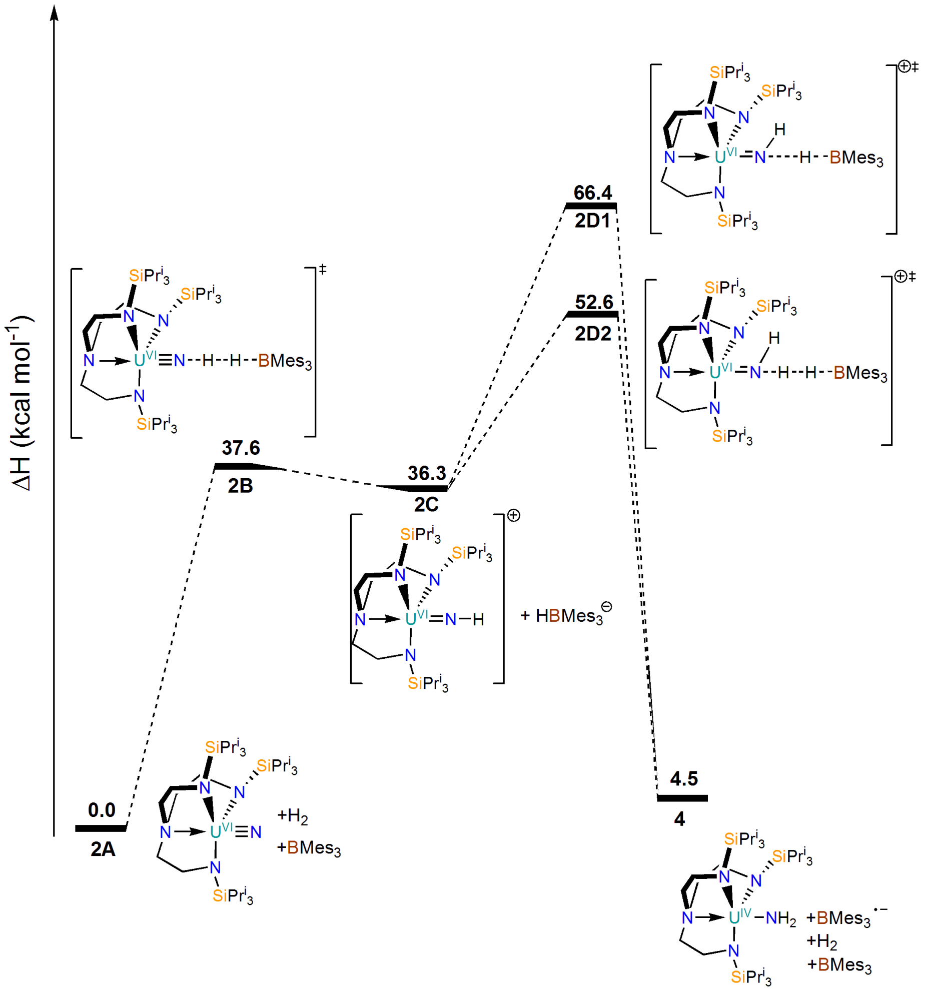

Supplementary Figure 14. Computed enthalpy profile at 298 K for the reaction of **2** with H_2.

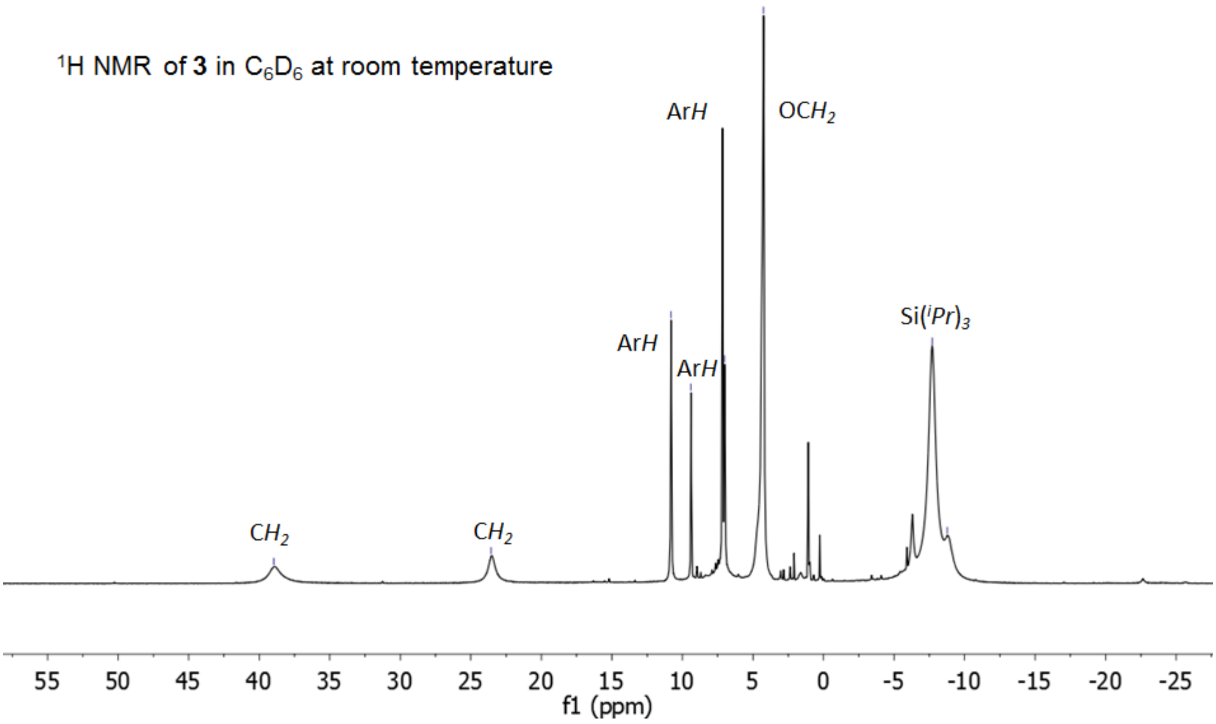

Supplementary Figure 15. ^{1}H NMR spectrum of complex **3** in C_6D_6 at 298 K.

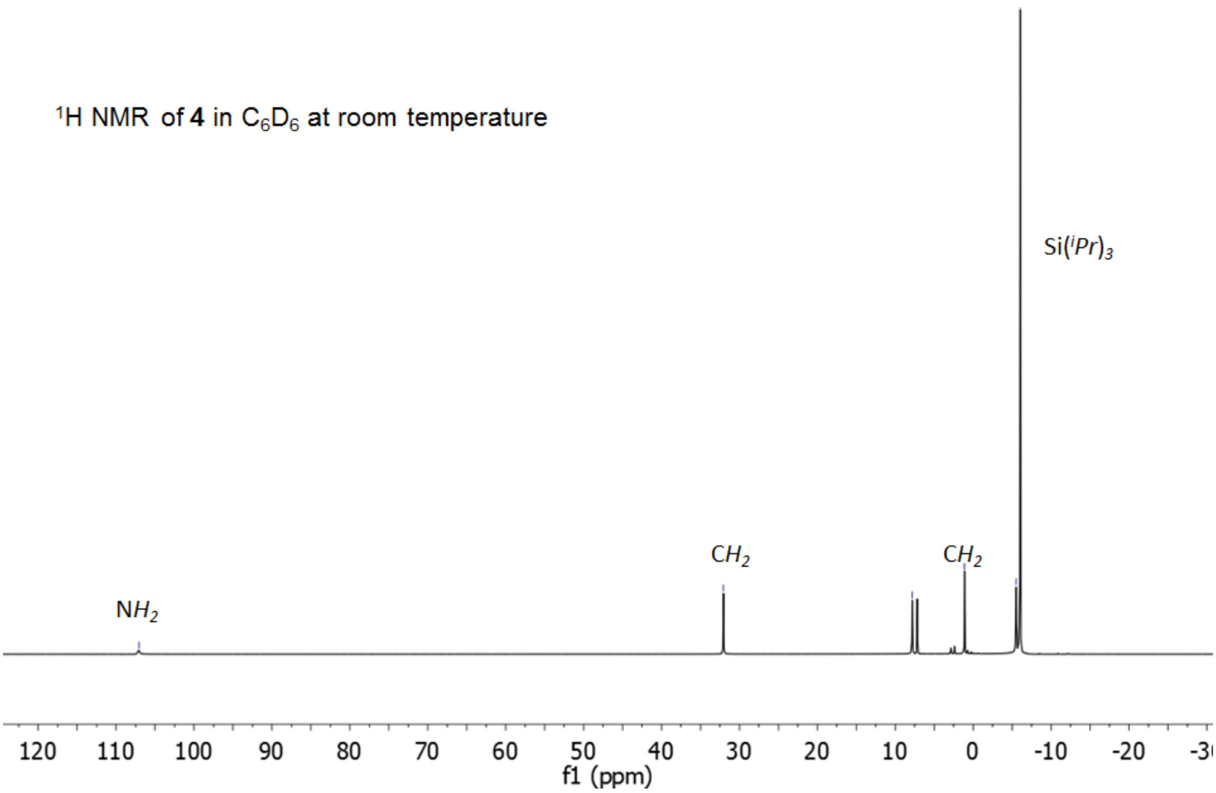

Supplementary Figure 16. ^{1}H NMR spectrum of complex **4** in C_6D_6 at 298 K.

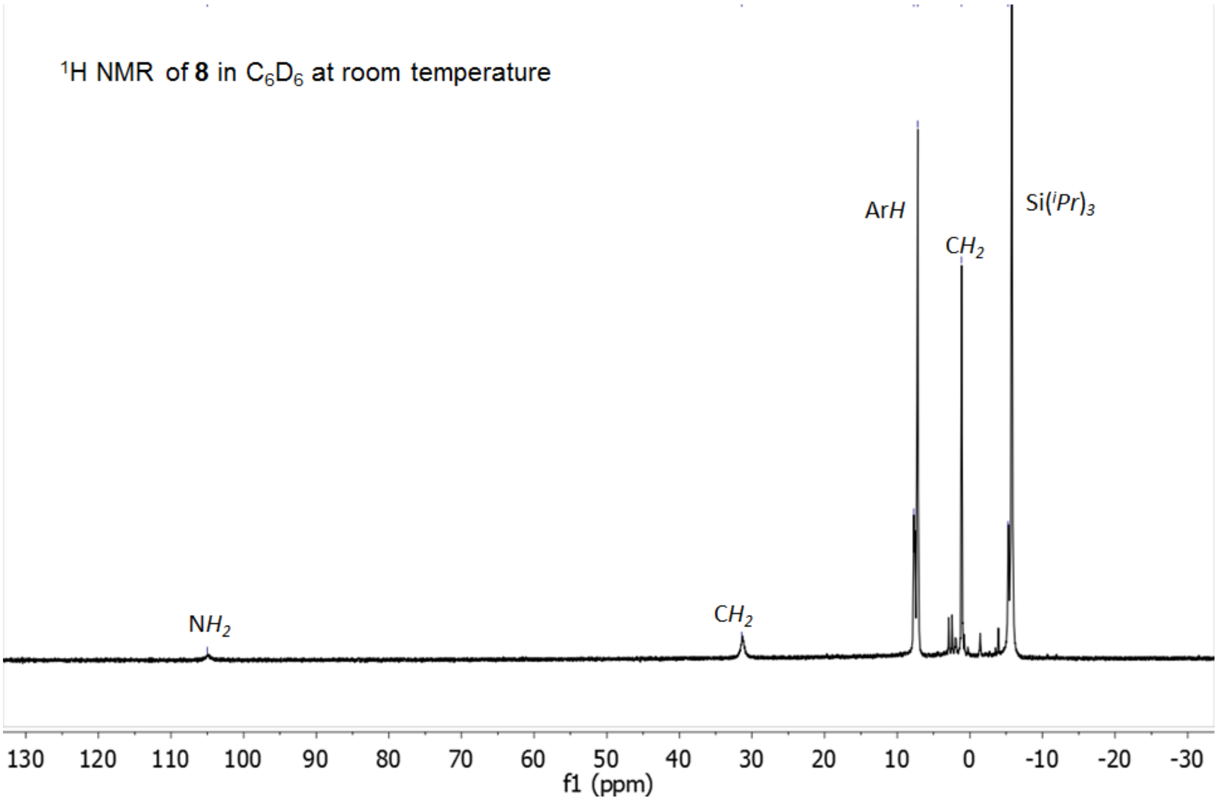

Supplementary Figure 17. ^{1}H NMR spectrum of complex **8** in C_6D_6 at 298 K.

Supplementary Tables

Supplementary Table 1. H_2 optimised geometry and energy

Energy: -1.177516 au

H	0.000000	0.000000	0.132239
H	0.000000	0.000000	0.875761

Supplementary Table 2. $BMes_3$ optimised geometry and energy

Energy: -1073.279840 au

B	0.094376	0.170075	-0.210917
C	0.847420	1.447058	-0.766331
C	0.647900	2.727864	-0.185702
C	1.313911	3.843403	-0.697753
C	2.210095	3.747623	-1.763021
C	2.418403	2.486745	-2.320605
C	1.748623	1.350416	-1.858995
C	0.088018	-0.132912	1.342552
C	1.287974	-0.102956	2.101784
C	1.261014	-0.381943	3.470074
C	0.074210	-0.667672	4.145443
C	-1.105502	-0.679442	3.402089
C	-1.118529	-0.437671	2.025913
C	-0.649877	-0.804663	-1.211962
C	-1.542902	-0.305332	-2.198569
C	-2.192194	-1.183117	-3.067591
C	-1.972666	-2.561687	-3.029575

C	-1.086215	-3.048145	-2.071000
C	-0.440030	-2.206321	-1.160239
C	2.633461	0.205352	1.485271
C	0.072321	-0.977189	5.618412
C	-2.458395	-0.488171	1.327439
C	-1.849211	1.167984	-2.342477
C	-2.668678	-3.482378	-3.995792
C	0.489133	-2.854893	-0.159263
C	-0.288302	2.955164	0.979387
C	2.904981	4.967298	-2.305896
C	2.049948	0.040744	-2.551212
H	2.196477	-0.368232	4.027331
H	-2.047327	-0.886566	3.907654
H	-2.636134	0.388248	0.696510
H	-2.541536	-1.363881	0.676687
H	-3.270008	-0.534864	2.059504
H	3.438245	-0.022715	2.190342
H	2.820873	-0.373903	0.575848
H	2.718613	1.260101	1.206931
H	-0.891029	-0.736397	6.077642
H	0.261466	-2.043129	5.797342
H	0.850807	-0.417972	6.146602
H	-2.886444	-0.776998	-3.801741
H	-0.892765	-4.118605	-2.024881
H	1.459073	-2.352204	-0.098314
H	0.067303	-2.842773	0.850447
H	0.676947	-3.897645	-0.431846
H	-2.696983	1.319076	-3.017215
H	-2.102220	1.638336	-1.387096
H	-0.994705	1.719725	-2.745437
H	-2.419364	-4.529278	-3.802069
H	-3.757726	-3.378567	-3.930673
H	-2.387408	-3.257874	-5.031366
H	1.131002	4.817100	-0.245758
H	3.122029	2.382450	-3.144914
H	-0.463513	4.025007	1.126413
H	-1.263155	2.480097	0.833385
H	0.120187	2.550535	1.910500
H	1.199848	-0.305864	-3.146470
H	2.907660	0.150553	-3.221353
H	2.285875	-0.762234	-1.846012
H	3.824720	4.702813	-2.835718
H	2.262002	5.503985	-3.014738
H	3.162251	5.670917	-1.507835

Supplementary Table 3. $HBMes_3^-$ optimised geometry and energy

Energy: -1073.921415 au

C	1.654593	3.318182	1.637881
C	2.911839	2.920562	2.177944
C	3.948979	3.886622	2.087494

C	3.732304	5.138181	1.492974
C	2.499380	5.510007	0.964826
C	1.467143	4.577671	1.058117
B	3.046036	1.388048	2.788816
C	4.555354	0.714684	2.706506
C	5.067978	0.371877	1.422094
C	6.316733	-0.240214	1.267286
C	7.124541	-0.559625	2.357625
C	6.639333	-0.226326	3.618996
C	5.395625	0.393697	3.805698
C	4.289858	0.664416	0.161594
C	8.452052	-1.251028	2.179248
C	5.022318	0.715750	5.235048
C	5.335420	3.646314	2.640437
C	2.293833	6.849089	0.304058
C	0.457873	2.398107	1.673697
C	2.201544	1.093893	4.180709
C	1.737744	-0.232771	4.416098
C	0.974872	-0.557053	5.543131
C	0.615626	0.398372	6.492992
C	1.067251	1.697568	6.280469
C	1.840354	2.051365	5.165074
C	2.055789	-1.357591	3.460137
C	-0.238005	0.042807	7.683362
C	2.277966	3.496426	5.091302
H	2.422346	0.726228	1.963159
H	0.481628	4.835858	0.667243
H	4.559071	5.849217	1.453415
H	5.308365	3.238667	3.654810
H	5.899031	2.924293	2.041317
H	5.905159	4.582904	2.668010
H	3.021000	7.587140	0.660660
H	2.405152	6.788431	-0.787698
H	1.290795	7.245759	0.500303
H	7.252250	-0.444767	4.494961
H	6.667614	-0.473310	0.260692
H	5.891817	0.603944	5.893917
H	4.646585	1.736869	5.341216
H	4.227637	0.062803	5.609676
H	8.953542	-0.928626	1.259290
H	9.126746	-1.044922	3.017765
H	8.340447	-2.342658	2.116729
H	0.820480	2.468116	7.012628
H	0.651993	-1.590266	5.680679
H	2.099586	4.001492	6.048215
H	3.340767	3.591270	4.852713
H	1.744293	4.049439	4.311930
H	3.130106	-1.430478	3.266077
H	1.706752	-2.318420	3.856707
H	1.583736	-1.192283	2.484681

H	0.018128	-0.945497	8.083207
H	-0.119118	0.771876	8.492579
H	-1.306499	0.015943	7.427492
H	4.880170	0.417090	-0.728694
H	3.356964	0.089696	0.131202
H	3.996702	1.717164	0.103984
H	-0.439649	2.909205	1.305570
H	0.626948	1.506769	1.058554
H	0.260317	2.032498	2.685902

Supplementary Table 4. $BMes_3^{-\bullet}$ optimised geometry and energy

Energy: -1073.300382 au

B	0.094774	0.168990	-0.211868
C	0.863726	1.447486	-0.763078
C	0.736767	2.734279	-0.152349
C	1.420061	3.846249	-0.648085
C	2.261434	3.770154	-1.761403
C	2.394259	2.521560	-2.368132
C	1.725711	1.385930	-1.899013
C	0.073239	-0.134160	1.348158
C	1.245727	-0.029900	2.161252
C	1.209031	-0.300161	3.530335
C	0.035667	-0.685069	4.184842
C	-1.115487	-0.791313	3.405074
C	-1.116961	-0.528473	2.031254
C	-0.648424	-0.804383	-1.225874
C	-1.469161	-0.313873	-2.289561
C	-2.108960	-1.182685	-3.175455
C	-1.988842	-2.571595	-3.077257
C	-1.193701	-3.064701	-2.043176
C	-0.536963	-2.224878	-1.137746
C	2.584920	0.335643	1.568162
C	0.021044	-0.974767	5.663202
C	-2.438827	-0.638879	1.311270
C	-1.714170	1.163924	-2.476178
C	-2.692929	-3.489775	-4.042129
C	0.328942	-2.883052	-0.091414
C	-0.175002	2.955300	1.030213
C	2.983588	4.985283	-2.282785
C	1.987469	0.085460	-2.618810
H	2.133665	-0.221176	4.104749
H	-2.053142	-1.075893	3.885321
H	-2.607617	0.229657	0.664533
H	-2.481668	-1.513732	0.653152
H	-3.267670	-0.709793	2.025912
H	3.395222	0.124810	2.276398
H	2.772297	-0.224397	0.645250
H	2.642214	1.394256	1.292058
H	-0.987816	-1.226809	6.007615
H	0.676127	-1.817537	5.923384

H	0.365365	-0.115088	6.253697
H	-2.735252	-0.759979	-3.962950
H	-1.066491	-4.144029	-1.944676
H	1.308292	-2.395095	-0.028651
H	-0.104777	-2.811478	0.912101
H	0.480818	-3.944533	-0.321383
H	-2.537365	1.336971	-3.179793
H	-1.962667	1.644405	-1.522958
H	-0.828671	1.687218	-2.852774
H	-2.439250	-4.537752	-3.848810
H	-3.785769	-3.399480	-3.972684
H	-2.425155	-3.272870	-5.084853
H	1.279267	4.809973	-0.155648
H	3.054048	2.422359	-3.231608
H	-0.321907	4.026141	1.215442
H	-1.153964	2.491942	0.862985
H	0.217367	2.502014	1.946868
H	2.188751	-0.724065	-1.907937
H	1.124872	-0.242944	-3.208874
H	2.845936	0.179062	-3.294883
H	3.650964	4.724586	-3.111423
H	2.287308	5.750925	-2.652059
H	3.594077	5.464215	-1.505784

Supplementary Table 5. U(V)-nitride (1) optimised geometry and energy

Energy: -2064.947781 au

H	-2.950727	-0.351591	-3.189171
H	-3.989671	0.900409	-2.504551
H	-3.733480	2.463641	-0.751780
H	-5.149514	3.487808	-0.468209
C	-3.739003	-0.163709	-2.446599
H	-4.623380	-0.726259	-2.771825
C	-4.618110	2.590198	-0.118561
H	-5.828067	1.178431	-1.181319
H	-4.255771	2.794608	0.894994
H	-2.397615	0.040853	-0.783005
H	-5.924425	-2.393359	-1.519056
C	-5.523246	1.353531	-0.137735
H	-7.327550	2.511883	0.283326
H	-7.018533	-1.037371	-1.235232
C	-3.265120	-0.581378	-1.047921
H	-1.926287	-2.126069	-1.797133
H	2.039326	3.808500	-0.388856
H	-0.334440	3.269451	-0.871891
C	-6.597501	-1.941728	-0.781051
C	-6.804499	1.621121	0.662436
C	-2.749110	-2.024864	-1.076941
H	-7.426734	-2.649082	-0.629898
H	-3.532073	-2.734792	-1.372228
H	-1.200122	-5.720386	1.065802

Si	-4.524650	-0.238989	0.370246
H	-7.511688	0.785681	0.612845
C	1.832433	3.986683	0.675943
H	-6.590620	1.806566	1.721013
H	-1.697211	-4.057261	1.396233
C	-0.588957	3.553397	0.158089
H	-0.129965	-4.369173	0.659165
N	-0.403388	-0.063831	0.771428
H	1.730339	5.071867	0.803436
H	-0.824815	4.625381	0.152501
H	0.764244	2.151867	0.990352
C	-0.839099	-4.738465	1.405900
H	-2.356204	-2.334392	-0.104825
H	-4.649840	-3.460199	0.518876
H	2.722424	3.674773	1.232181
C	-5.865322	-1.642573	0.532582
H	-1.500785	3.010422	0.422958
C	0.570403	3.227968	1.108921
C	-5.344101	-2.930148	1.179099
H	2.760603	1.965642	2.888248
H	-6.613714	-1.222467	1.223252
H	-1.592856	-6.423371	3.426289
N	-3.668923	-0.051791	1.894671
H	1.050207	-2.232707	1.123662
H	-6.165109	-3.624880	1.411306
H	2.027515	-4.557622	1.069467
H	-4.803013	-2.721364	2.107226
H	3.252649	-3.358723	0.644263
H	-2.194363	-4.803892	3.773024
U	-1.309385	-0.003359	2.295634
C	-0.238083	-4.821628	2.815259
H	2.038448	1.507804	4.424250
H	-2.426586	5.017255	1.777710
C	-1.288813	-5.420003	3.760330
C	2.581087	2.310415	3.911954
H	0.618767	-5.514830	2.783311
H	-5.564363	-0.223906	2.905135
C	2.594671	-3.703827	1.454386
C	-4.554248	0.209468	3.023932
H	3.559519	2.417597	4.403350
C	1.696846	-2.561153	1.948599
H	3.151577	-1.012099	1.467199
H	-0.149298	6.334693	1.727738
H	-4.732979	1.290751	3.175570
Si	0.110678	3.412985	2.971231
H	2.351498	4.379626	3.399268
H	-0.939659	-5.510905	4.792560
C	-2.348534	5.002758	2.869617
C	1.780286	3.616406	3.950182
H	-2.850222	4.092107	3.211036

Si	0.503665	-3.107408	3.360570
N	-0.725260	-1.891921	3.654917
N	-0.811484	2.013150	3.498509
H	3.249527	-4.081155	2.251835
C	2.565044	-1.356612	2.329728
H	-2.448167	-2.739883	4.603478
H	-2.915503	5.866835	3.247197
C	-0.189904	6.313708	2.822976
C	-0.888217	5.046456	3.331341
H	-3.995784	-1.475084	4.209770
H	0.840216	6.395898	3.188494
H	3.276568	-1.604715	3.128221
C	-4.013118	-0.387040	4.318921
H	1.962564	-0.507870	2.664360
C	-1.538265	-2.157189	4.835345
N	-2.635621	0.055091	4.598317
H	-0.723001	7.222597	3.140031
H	1.367478	-5.551486	5.222693
H	-1.018400	-2.756997	5.604762
H	-4.669038	-0.146450	5.176654
C	1.645748	4.112432	5.395461
H	-0.904946	5.114342	4.430650
H	2.632888	4.223613	5.868747
H	-3.341100	2.014858	4.523539
C	-1.239768	2.089007	4.890035
H	2.645244	-3.574701	4.413743
H	1.075961	3.408875	6.012905
C	1.674156	-3.398675	4.904854
C	-1.950526	-0.862266	5.525804
C	-2.605021	1.441743	5.094580
H	-0.514151	1.616741	5.578297
H	1.144937	5.084969	5.460134
C	1.420469	-4.620012	5.796133
H	0.483895	-4.528110	6.359887
H	-1.346551	3.122225	5.267932
H	-2.590918	-1.057788	6.406616
H	-1.042473	-0.362138	5.874501
H	2.059668	-1.253125	5.172459
H	-2.909220	1.469624	6.157944
H	2.223996	-4.739989	6.538969
C	1.839362	-2.141921	5.769413
H	0.927438	-1.926969	6.337852
H	2.652348	-2.266423	6.500549

Supplementary Table 6. Complex A optimised geometry and energy

Energy: -2066.113976 au

U	0.010137	-0.213981	-0.122809
C	-3.292862	-0.054748	0.470131
C	-2.719284	-0.622476	1.764859
N	-1.389304	-0.078445	2.087021

C	-0.722048	-0.883731	3.125251
C	-0.131188	-2.165955	2.547826
N	0.775523	-1.880060	1.442075
Si	2.172276	-2.936499	1.387984
C	3.281410	-2.809751	2.999811
C	3.207464	-3.941839	4.031721
N	0.471164	1.871943	0.943467
Si	1.412414	3.262548	0.412683
C	0.382469	4.900921	0.642340
C	-1.055216	4.824122	0.121750
N	-2.361400	-0.147499	-0.647679
Si	-3.190473	-0.102052	-2.200173
C	-1.913987	-0.055602	-3.652920
C	-1.467740	-1.432910	-4.162460
N	0.948935	-0.238404	-1.627522
C	-0.074357	2.024055	2.290323
C	-1.436204	1.349696	2.445906
C	-4.321171	1.475054	-2.390806
C	-3.559579	2.746760	-2.001891
C	-4.424220	-1.600842	-2.336255
C	-5.171320	-1.673053	-3.673990
C	-5.675874	1.431308	-1.669954
C	1.749025	-4.822206	1.121864
C	1.279174	-5.129958	-0.306147
C	3.346143	-2.450851	-0.061100
C	4.458757	-3.482398	-0.295847
C	0.744732	-5.432329	2.109699
C	1.982621	2.998293	-1.406556
C	3.245586	3.770000	-1.811187
C	3.011892	3.580418	1.480008
C	3.915652	2.345891	1.533546
C	0.870777	3.239324	-2.435674
C	2.761905	4.105831	2.899553
C	1.091219	6.148793	0.100136
C	-3.805924	-2.954572	-1.972474
C	-2.342184	0.804180	-4.851400
C	3.963309	-1.059716	0.110826
C	3.145036	-1.455035	3.704594
H	-1.551701	0.812531	-5.615012
H	-2.539779	1.846382	-4.581926
H	-2.622314	2.854955	-2.557180
H	-4.159358	3.650763	-2.184792
H	-3.247137	0.413902	-5.335703
H	-4.538472	1.534517	-3.468423
H	-3.292886	2.737952	-0.939798
H	-1.020700	0.401301	-3.203329
H	-4.491797	-1.900179	-4.503397
H	-6.260328	2.340317	-1.877818
H	-5.677171	-0.731976	-3.918779
H	-0.656037	-1.322656	-4.894287

H	3.529774	3.524880	-2.844469
H	1.188648	2.904648	-3.432142
H	-5.937934	-2.462411	-3.664730
H	-2.282957	-1.970983	-4.662666
H	1.174094	-6.213607	-0.464615
H	-6.288018	0.576545	-1.977545
H	-5.558941	1.373019	-0.582094
H	0.299935	-4.679984	-0.498565
H	1.960104	-4.748986	-1.072976
H	3.098891	4.856782	-1.771051
H	0.610707	4.302777	-2.515884
H	2.206108	1.922582	-1.450568
H	-1.085915	-2.066940	-3.360630
H	-3.083647	-3.284694	-2.726569
H	4.105315	3.533396	-1.176183
H	-0.038957	2.684665	-2.189766
H	4.185432	1.983176	0.536691
H	-5.180224	-1.382509	-1.565142
H	0.644154	-6.516346	1.948899
H	2.711009	-2.403420	-0.956636
H	-4.573263	-3.739108	-1.892837
H	4.077726	-4.486112	-0.510379
H	-3.273309	-2.901547	-1.017490
H	5.090493	-3.184769	-1.144612
H	-0.252014	-4.998845	1.973201
H	3.412314	1.518793	2.045122
H	-1.086889	4.758319	-0.970282
H	2.713458	-5.335123	1.274478
H	-4.235624	-0.612759	0.311772
H	4.850292	2.550559	2.076759
H	4.592692	-0.804671	-0.753145
H	1.169908	6.121422	-0.993043
H	-3.622526	0.984924	0.660312
H	3.555970	4.369422	0.937013
H	1.026164	-5.283281	3.155886
H	-1.574287	3.941670	0.506686
H	5.122620	-3.566392	0.575185
H	-0.959900	-2.837907	2.254460
H	-1.635454	5.713213	0.410978
H	-2.605007	-1.705164	1.646706
H	2.106697	6.258667	0.497437
H	4.598304	-1.001085	1.004263
H	3.194681	-0.286607	0.181396
H	0.539441	7.065856	0.355992
H	3.348943	-4.930523	3.582544
H	0.352461	-2.685867	3.395348
H	-3.414574	-0.452285	2.608676
H	0.315708	5.028002	1.734157
H	3.710866	4.314214	3.416201
H	-2.135559	1.838497	1.762546

H	4.297991	-2.846733	2.575669
H	2.223161	3.373517	3.511645
H	0.608292	1.628841	3.065473
H	2.178825	5.033080	2.908005
H	2.243002	-3.953383	4.554568
H	-0.241587	3.076917	2.580449
H	-1.428351	-1.095049	3.949576
H	0.099322	-0.287990	3.531608
H	3.220901	-0.616578	3.007927
H	-1.825645	1.470730	3.474099
H	3.981527	-3.821104	4.804826
H	2.174814	-1.365981	4.206835
H	3.920048	-1.326325	4.474917
H	-0.119856	-2.341232	-1.474095
H	-0.654757	-2.628756	-1.022423

Supplementary Table 7. Complex B (transition state) optimised geometry and energy

Energy: -2066.101855 au

U	-1.325850	-0.200293	2.410941
C	-4.591839	0.094242	3.022464
C	-4.059763	-0.516671	4.315949
N	-2.710012	-0.035898	4.649055
C	-2.065495	-0.887638	5.664249
C	-1.462942	-2.138169	5.034996
N	-0.511698	-1.793605	3.982116
Si	0.837581	-2.912283	3.864678
C	1.864389	-2.983404	5.533064
C	1.619172	-4.164746	6.478998
N	-0.852330	1.883016	3.454549
Si	0.049818	3.296620	2.911247
C	-1.021281	4.907449	3.141322
C	-2.458290	4.794407	2.625985
N	-3.662926	-0.051103	1.906395
Si	-4.501015	-0.130023	0.355239
C	-3.241670	-0.246314	-1.106822
C	-2.855485	-1.679854	-1.499388
N	-0.291754	-0.207701	0.883457
C	-1.322078	2.013444	4.833869
C	-2.696592	1.383645	5.040043
C	-5.598095	1.454340	0.048596
C	-4.797392	2.733098	0.316054
C	-5.771427	-1.603156	0.367815
C	-6.552411	-1.756337	-0.944153
C	-6.935195	1.504065	0.800434
C	0.338288	-4.736970	3.401030
C	-0.011167	-4.887634	1.913592
C	2.088212	-2.358502	2.510191
C	3.213631	-3.379504	2.291055
C	-0.794877	-5.344116	4.239411
C	0.623586	3.036134	1.092455

C	1.865052	3.840429	0.684378
C	1.641834	3.655684	3.977935
C	2.561646	2.434425	4.057642
C	-0.495888	3.242172	0.064075
C	1.388877	4.211094	5.385770
C	-0.346762	6.173729	2.598261
C	-5.180865	-2.943324	0.819560
C	-3.657276	0.526991	-2.366732
C	2.689528	-0.973224	2.758785
C	1.805828	-1.664618	6.312952
H	-2.876637	0.447743	-3.136364
H	-3.817619	1.592850	-2.178729
H	-3.861439	2.764105	-0.251450
H	-5.372308	3.634669	0.057106
H	-4.580155	0.128144	-2.808223
H	-5.839766	1.429713	-1.025223
H	-4.522819	2.808780	1.373092
H	-2.327682	0.220962	-0.711291
H	-5.898102	-2.064310	-1.767515
H	-7.506318	2.404333	0.527697
H	-7.040247	-0.823857	-1.250653
H	-2.030741	-1.666076	-2.225278
H	2.151834	3.603615	-0.350009
H	-0.168093	2.917973	-0.932578
H	-7.337576	-2.522310	-0.857566
H	-3.690150	-2.212097	-1.972462
H	-0.275860	-5.928556	1.674383
H	-7.571250	0.639529	0.581978
H	-6.792844	1.538217	1.886293
H	-0.865267	-4.255205	1.647059
H	0.813054	-4.599927	1.253843
H	1.691198	4.923064	0.726817
H	-0.789606	4.296803	-0.015136
H	0.880489	1.968155	1.052341
H	-2.521582	-2.264276	-0.638544
H	-4.527981	-3.374230	0.053906
H	2.732192	3.623868	1.316559
H	-1.387395	2.659115	0.310488
H	2.835486	2.054628	3.068304
H	-6.503872	-1.305094	1.134806
H	-0.930184	-6.409979	4.002371
H	1.497578	-2.285576	1.586948
H	-5.971219	-3.680881	1.024239
H	2.843533	-4.383036	2.057218
H	-4.575631	-2.830014	1.724308
H	3.863371	-3.068772	1.460799
H	-1.747061	-4.848644	4.022319
H	2.071502	1.611073	4.588323
H	-2.493931	4.738484	1.533455
H	1.247479	-5.329943	3.597930

H	-5.564363	-0.403336	2.855382
H	3.494195	2.664544	4.594251
H	3.344080	-0.676796	1.927260
H	-0.272257	6.148913	1.504720
H	-4.855990	1.151930	3.212145
H	2.176136	4.439216	3.417579
H	-0.622331	-5.268473	5.317197
H	-2.948216	3.893178	3.005557
H	3.856831	-3.468189	3.176667
H	-2.285858	-2.779527	4.668515
H	-3.062293	5.663497	2.926732
H	-3.995077	-1.602425	4.185469
H	0.667190	6.309873	2.991404
H	3.296368	-0.949771	3.673160
H	1.914914	-0.207872	2.842845
H	-0.921236	7.075845	2.856958
H	1.690695	-5.130547	5.968054
H	-1.014034	-2.715277	5.863404
H	-4.753733	-0.324194	5.156051
H	-1.088719	5.030339	4.233764
H	2.337335	4.444632	5.892310
H	-3.411262	1.914100	4.405990
H	2.896926	-3.092907	5.164043
H	0.863249	3.486512	6.017921
H	-0.614292	1.567589	5.556676
H	0.793424	5.130353	5.374705
H	0.627484	-4.114061	6.945722
H	-1.431474	3.062200	5.162379
H	-2.788761	-1.139070	6.461573
H	-1.253395	-0.312654	6.117292
H	2.007040	-0.797233	5.678641
H	-3.031651	1.491769	6.088534
H	2.352154	-4.173476	7.300138
H	0.814922	-1.511104	6.755063
H	2.533602	-1.655606	7.138349
H	-0.870118	-1.427512	0.834135
H	-1.518450	-2.081591	1.280655

Supplementary Table 8. Complex C optimised geometry and energy

Energy: -2066.124874 au

U	-0.007170	-0.261884	-0.101435
C	-3.247219	0.185072	0.556181
C	-2.749429	-0.436077	1.858280
N	-1.382478	-0.012407	2.182756
C	-0.763933	-0.883264	3.194009
C	-0.170535	-2.127383	2.546632
N	0.792441	-1.766733	1.508996
Si	2.102424	-2.928990	1.339717
C	3.151260	-3.048674	2.989992
C	2.893658	-4.244004	3.915031

N	0.524033	1.839644	0.899513
Si	1.394653	3.282143	0.377832
C	0.292921	4.867236	0.648793
C	-1.135831	4.740996	0.112867
N	-2.306985	-0.006710	-0.542533
Si	-3.118137	-0.172969	-2.104166
C	-1.840207	-0.286494	-3.551337
C	-1.346404	-1.709925	-3.846598
N	1.118641	-0.050842	-1.651649
C	0.105334	1.960817	2.300148
C	-1.293201	1.406071	2.550920
C	-4.248492	1.373927	-2.477397
C	-3.471642	2.679922	-2.279892
C	-4.347726	-1.676359	-2.064343
C	-5.107235	-1.875830	-3.382648
C	-5.583420	1.435673	-1.723499
C	1.552945	-4.729403	0.848943
C	1.185073	-4.838039	-0.637987
C	3.342021	-2.372392	-0.025493
C	4.440522	-3.413764	-0.281571
C	0.412085	-5.322314	1.687077
C	1.976932	3.106706	-1.448643
C	3.209851	3.937254	-1.826829
C	2.990533	3.644467	1.441117
C	3.924973	2.432595	1.491624
C	0.858245	3.328632	-2.473716
C	2.751894	4.177743	2.859811
C	0.948801	6.160910	0.149354
C	-3.727364	-2.990732	-1.578957
C	-2.305674	0.377509	-4.855780
C	3.980625	-1.006388	0.238865
C	3.127921	-1.745077	3.797375
H	-1.521611	0.299761	-5.622421
H	-2.536677	1.440569	-4.736744
H	-2.541282	2.701391	-2.857227
H	-4.066512	3.556574	-2.576872
H	-3.199406	-0.109223	-5.267483
H	-4.494343	1.289251	-3.547165
H	-3.189766	2.811753	-1.230392
H	-0.966646	0.271701	-3.182019
H	-4.433092	-2.187437	-4.188820
H	-6.180819	2.299806	-2.051341
H	-5.612098	-0.961832	-3.716298
H	-0.537975	-1.684684	-4.591698
H	3.495405	3.748147	-2.871959
H	1.195368	3.053512	-3.483388
H	-5.875192	-2.658207	-3.289346
H	-2.143492	-2.338820	-4.263398
H	0.856930	-5.858342	-0.887733
H	-6.195200	0.540842	-1.881564

H	-5.437210	1.545441	-0.643364
H	0.374012	-4.143066	-0.885684
H	2.023455	-4.594129	-1.298131
H	3.028838	5.015863	-1.738559
H	0.545624	4.379613	-2.516738
H	2.240124	2.041652	-1.504943
H	-0.951989	-2.188058	-2.944629
H	-3.054486	-3.418431	-2.328488
H	4.081144	3.701370	-1.207506
H	-0.022447	2.720714	-2.250554
H	4.189320	2.069466	0.493222
H	-5.095307	-1.379455	-1.311519
H	0.254492	-6.381923	1.436584
H	2.731573	-2.275225	-0.934240
H	-4.502463	-3.742623	-1.367110
H	4.044023	-4.404685	-0.524348
H	-3.128796	-2.843767	-0.675369
H	5.083702	-3.104320	-1.117694
H	-0.529570	-4.804468	1.478583
H	3.450298	1.596520	2.016310
H	-1.157706	4.736236	-0.981545
H	2.448686	-5.348474	1.028439
H	-4.234201	-0.277333	0.377277
H	4.862300	2.665112	2.018883
H	4.637316	-0.713572	-0.593147
H	1.041441	6.165007	-0.943055
H	-3.470338	1.254289	0.730631
H	3.509338	4.442353	0.886449
H	0.591357	-5.264804	2.765223
H	-1.604090	3.809039	0.442357
H	5.095978	-3.529796	0.591688
H	-0.995359	-2.747174	2.151298
H	-1.766260	5.578695	0.446446
H	-2.732007	-1.524468	1.734712
H	1.953331	6.307980	0.562421
H	4.598577	-1.015666	1.145920
H	3.229276	-0.220823	0.346069
H	0.351563	7.044312	0.420904
H	2.938913	-5.200485	3.384016
H	0.267999	-2.731185	3.361230
H	-3.439460	-0.206809	2.693501
H	0.211244	4.957633	1.743307
H	3.703932	4.434645	3.348155
H	-1.995482	1.965571	1.928057
H	4.177010	-3.170565	2.606004
H	2.262427	3.431490	3.495453
H	0.814331	1.460733	2.984439
H	2.129036	5.078770	2.871256
H	1.908609	-4.182472	4.394176
H	0.067924	3.006063	2.653325

H	-1.497390	-1.141884	3.979738
H	0.049696	-0.327221	3.667552
H	3.328769	-0.867786	3.176704
H	-1.592896	1.549092	3.606564
H	3.635735	-4.284225	4.727029
H	2.147982	-1.585842	4.261545
H	3.871596	-1.764120	4.608247
H	1.685929	-0.069032	-2.496520
H	-0.421799	-2.091242	-0.913973

Supplementary Table 9. Complex D (transition state) optimised geometry and energy

Energy: -2066.070730 au

H	0.042627	0.116071	0.093383
N	-0.013860	0.039881	2.032242
U	1.919143	0.117174	1.210168
N	2.436077	-1.936022	0.182758
C	3.680501	-1.871642	-0.595396
C	4.862494	-1.442583	0.269993
N	4.685250	-0.094691	0.834366
C	5.047933	1.004350	-0.068511
C	4.176285	2.238673	0.177711
N	2.749123	1.990360	-0.053223
Si	2.054250	2.987734	-1.317059
C	0.148622	2.759451	-1.250778
C	-0.497786	3.286019	0.038564
Si	1.434690	-3.340439	-0.164197
C	-0.041551	-3.330963	1.066276
C	0.321590	-3.954048	2.424271
N	3.084419	0.224813	3.300022
Si	2.458426	0.605901	4.895174
C	3.971378	0.988729	6.065941
C	3.652525	1.313191	7.533998
C	0.665890	-3.335878	-1.954916
C	1.548513	-3.892624	-3.081001
C	0.161619	-1.936732	-2.318595
C	2.494339	-4.969865	-0.079194
C	3.481067	-5.029055	1.091817
C	1.631776	-6.236749	-0.125831
C	5.266897	0.054367	2.174966
C	4.348595	-0.513406	3.249240
C	1.541892	-0.852458	5.791651
C	2.297942	-2.164320	5.531705
C	1.214263	2.069265	4.740942
C	0.762237	2.649853	6.084494
C	0.067693	-1.013014	5.375597
C	2.619604	2.538049	-3.123563
C	4.126708	2.707067	-3.356928
C	2.608571	4.836321	-1.070358
C	2.187149	5.418291	0.284505
C	2.186428	1.128777	-3.523371

C	-1.323970	-3.973301	0.515404
C	1.649048	3.203008	3.803765
C	4.910955	2.050471	5.463462
C	2.219968	5.781505	-2.216955
C	-0.622553	3.239704	-2.485077
H	-0.247046	-2.267074	1.249714
H	-0.592703	0.883021	2.119268
H	4.565453	1.586492	8.084042
H	5.963889	1.866524	5.718056
H	2.956103	2.153402	7.633552
H	4.661623	3.045234	5.853724
H	3.208340	0.460413	8.056782
H	4.830836	2.105766	4.370959
H	4.520898	0.035259	6.087842
H	1.515679	3.330471	6.503176
H	2.350707	3.884227	4.300381
H	3.377651	-2.071054	5.710710
H	0.572789	1.869895	6.828424
H	-0.162872	3.233551	5.973689
H	1.573855	-0.652957	6.873018
H	4.924291	-0.456845	4.184301
H	5.399032	1.120800	2.380842
H	2.602199	6.426606	0.432817
H	0.788836	3.808331	3.483599
H	6.268878	-0.415584	2.214555
H	1.932604	-2.975948	6.177676
H	2.145501	2.836910	2.900020
H	2.516046	4.791749	1.120068
H	2.169991	-2.481916	4.492610
H	4.366363	2.602043	1.203799
H	1.099014	5.505088	0.359159
H	6.124868	1.242885	0.031547
H	4.206684	-1.595975	3.074060
H	0.344555	1.567718	4.288569
H	4.591046	3.032946	-0.465650
H	-0.583881	-0.325141	5.929909
H	2.685967	6.770333	-2.089078
H	3.708987	4.793766	-1.070436
H	-0.290967	-2.031951	5.582650
H	4.925445	-2.144242	1.105761
H	-0.077715	-0.822043	4.302712
H	5.816261	-1.511559	-0.287719
H	0.095823	3.046791	0.926102
H	4.879876	0.668097	-1.096901
H	1.136077	5.946951	-2.246264
H	-0.617960	4.377245	0.012033
H	1.339306	-3.706219	2.737688
H	2.519837	5.406223	-3.201881
H	4.478351	3.716108	-3.114108
H	-0.350444	-3.584969	3.210473

H	4.076730	-4.115432	1.169849
H	2.962310	-5.156052	2.050035
H	-1.496524	2.853968	0.181010
H	4.703655	2.001268	-2.750074
H	0.236742	-5.048766	2.411282
H	4.172383	-5.878042	0.988591
H	3.605897	-1.181649	-1.455822
H	3.970926	-2.840295	-1.036488
H	0.050989	1.659693	-1.208864
H	4.394175	2.514763	-4.406949
H	-0.461927	4.306688	-2.683669
H	2.114955	3.260837	-3.781881
H	2.646869	0.381380	-2.868486
H	-1.704239	3.099375	-2.349810
H	3.095231	-4.951071	-1.000576
H	-2.102763	-3.991194	1.291854
H	0.923530	-6.277625	0.710372
H	-0.336399	2.692873	-3.389400
H	2.247377	-7.147536	-0.076299
H	1.102837	0.992662	-3.458381
H	-1.176934	-5.012904	0.194836
H	2.489538	0.888274	-4.553600
H	0.959042	-1.194595	-2.237139
H	-1.733402	-3.415510	-0.332443
H	-0.636275	-1.609996	-1.645295
H	2.328516	-3.176209	-3.364259
H	1.042858	-6.292834	-1.047560
H	2.052190	-4.825902	-2.809987
H	-0.211630	-3.995114	-1.878620
H	-0.231047	-1.903561	-3.346124
H	0.955503	-4.094426	-3.984931

Supplementary Table 10. U(III)-amide (6) optimised geometry and energy

Energy: -2066.166396 au

C	-0.076172	2.437361	-6.160086
C	-0.212054	0.966025	-5.750124
Si	0.882609	0.476984	-4.212041
N	0.647993	-1.196150	-3.772037
U	0.699285	-2.232424	-1.603064
N	1.858359	-4.348048	-1.703838
Si	3.365965	-4.834066	-0.968409
C	3.158330	-5.655194	0.781183
C	2.470987	-7.025314	0.745696
C	-1.689795	0.578763	-5.630364
C	2.413087	-4.716082	1.735208
C	0.560305	-2.162847	-4.864398
C	-0.612255	-3.126985	-4.674503
N	-0.507209	-3.878944	-3.417312
C	0.364409	-5.053001	-3.549880
C	1.030471	-5.431598	-2.227718

C	-1.823177	-4.214029	-2.857791
C	-2.423760	-3.035687	-2.089817
N	-1.582682	-2.625531	-0.963958
Si	-2.388867	-2.543797	0.577218
C	-2.823968	-4.285895	1.335371
C	-1.577995	-5.167684	1.459719
C	-3.929084	-5.048804	0.593884
C	0.522476	1.574428	-2.667130
C	-0.967608	1.667022	-2.315960
C	1.141318	2.978002	-2.702071
C	2.702445	0.879432	-4.773949
C	3.707505	0.537544	-3.670117
C	3.101825	0.179839	-6.078508
C	-4.108782	-1.641908	0.431098
C	-4.823064	-1.443371	1.773255
C	-4.073248	-0.336931	-0.371006
C	-1.212038	-1.674964	1.842696
C	-1.470937	-2.014939	3.317151
C	-1.156780	-0.152263	1.661877
C	4.457747	-3.266145	-0.704474
C	5.537610	-3.387486	0.379223
C	5.080229	-2.744372	-2.004932
C	4.268456	-6.186758	-2.043563
C	5.642818	-6.592961	-1.496321
C	4.361817	-5.856963	-3.537766
N	1.804473	-0.797456	-0.164443
H	-0.609603	2.643395	-7.100344
H	-2.221424	0.724290	-6.582831
H	-0.503138	3.103780	-5.401251
H	-2.204536	1.185468	-4.878112
H	0.967876	2.735180	-6.306097
H	-1.812508	-0.466586	-5.334029
H	0.206353	0.368479	-6.575377
H	0.709818	3.598286	-3.497455
H	-1.518165	2.282161	-3.039232
H	2.461870	0.467172	-6.920506
H	2.224070	2.955000	-2.859679
H	0.961575	3.506219	-1.754445
H	2.747993	1.965852	-4.949826
H	0.414291	-1.699093	-5.856456
H	-1.530253	-2.533009	-4.633663
H	-5.088854	0.035990	-0.571399
H	-1.110998	2.130531	-1.329629
H	-0.703520	-3.817772	-5.535373
H	4.138152	0.418532	-6.361163
H	-1.441070	0.680878	-2.285113
H	-3.567327	-0.470163	-1.331205
H	3.041741	-0.910119	-5.976897
H	-2.582244	-2.204388	-2.800246
H	-3.540151	0.455232	0.164513

H	-2.508995	-4.563383	-3.654800
H	1.486122	-2.758461	-4.969623
H	1.003751	1.017786	-1.845519
H	-3.441772	-3.346765	-1.793871
H	3.501462	1.074919	-2.737847
H	-5.843751	-1.056672	1.634321
H	-4.719810	-2.348990	-0.152664
H	4.738778	0.777210	-3.969564
H	1.158736	-4.798913	-4.259517
H	3.673944	-0.531950	-3.437386
H	-0.196590	-5.910256	-3.971314
H	-0.940971	0.132595	0.628137
H	-1.689723	-5.041727	-2.153656
H	-4.293611	-0.719483	2.404345
H	-2.106322	0.314371	1.950712
H	4.342702	-2.672317	-2.810667
H	-4.902013	-2.376124	2.343377
H	-4.864992	-4.482219	0.536276
H	5.519271	-1.746424	-1.865423
H	3.393144	-5.560668	-3.950903
H	5.054778	-5.029807	-3.724690
H	-0.377330	0.293978	2.296650
H	-3.628599	-5.292086	-0.432129
H	5.887517	-3.400885	-2.353582
H	4.726195	-6.719823	-4.115525
H	0.241031	-5.739043	-1.516266
H	1.594442	-6.359576	-2.431650
H	-0.216293	-2.073231	1.587956
H	-4.156206	-6.003427	1.091827
H	3.730115	-2.513326	-0.358706
H	-2.460808	-1.674997	3.644990
H	-3.195761	-4.081763	2.352291
H	-1.119816	-5.336187	0.478312
H	-0.733322	-1.520769	3.965656
H	3.620841	-7.073280	-1.954569
H	6.077765	-2.436808	0.497670
H	6.365862	-5.772617	-1.579981
H	-1.412541	-3.088846	3.519539
H	6.062117	-7.443488	-2.054734
H	-0.808040	-4.713624	2.091456
H	6.286623	-4.148968	0.129840
H	-1.817335	-6.151317	1.890446
H	1.410566	-4.499349	1.351264
H	5.122588	-3.646848	1.358146
H	2.918341	-3.751689	1.852269
H	1.456085	-6.949401	0.338665
H	5.601720	-6.884779	-0.440933
H	3.017231	-7.751523	0.132782
H	4.176501	-5.803106	1.175014
H	2.300243	-5.158816	2.736247

H	2.379182	-7.453848	1.754944
H	1.548491	-0.471729	0.764186
H	2.690005	-0.343151	-0.385069

Supplementary Table 11. U(IV)-amide (4) optimised geometry and energy

Energy: -2066.144153 au

C	-0.017389	2.432434	-6.029182
C	-0.150849	0.952845	-5.640673
Si	0.874308	0.493955	-4.061172
N	0.665471	-1.230610	-3.662078
U	0.655912	-2.303951	-1.652867
N	1.747494	-4.316433	-1.686720
Si	3.332417	-4.768917	-0.998779
C	3.162057	-5.286952	0.853542
C	2.414287	-6.611785	1.050507
C	-1.627883	0.543824	-5.604884
C	2.512453	-4.178199	1.687850
C	0.656612	-2.162018	-4.803738
C	-0.531704	-3.115627	-4.723128
N	-0.507641	-3.876984	-3.458672
C	0.334135	-5.084584	-3.556789
C	0.938773	-5.432663	-2.202180
C	-1.866048	-4.185892	-2.972513
C	-2.430446	-3.001312	-2.198636
N	-1.536868	-2.633371	-1.087901
Si	-2.341200	-2.576224	0.495784
C	-2.748325	-4.337324	1.190137
C	-1.502304	-5.222036	1.291839
C	-3.864733	-5.085384	0.449040
C	0.390664	1.521610	-2.507013
C	-1.112511	1.508486	-2.203421
C	0.913326	2.965450	-2.517317
C	2.716450	0.901241	-4.476018
C	3.639162	0.626939	-3.283501
C	3.239347	0.180642	-5.725312
C	-4.043917	-1.664156	0.371748
C	-4.716092	-1.478417	1.739523
C	-4.033810	-0.349637	-0.415399
C	-1.120814	-1.738742	1.735620
C	-1.360030	-2.106255	3.208212
C	-1.058980	-0.212088	1.584825
C	4.489034	-3.230449	-1.077498
C	5.640032	-3.249119	-0.061069
C	5.034418	-2.946310	-2.483173
C	4.070380	-6.305852	-1.918273
C	5.440363	-6.710032	-1.353052
C	4.135643	-6.211507	-3.447864
N	1.792178	-0.948046	-0.305054
H	-0.484426	2.624594	-7.004441
H	-2.100941	0.689059	-6.585406

H	-0.517647	3.084304	-5.304210
H	-2.194494	1.141192	-4.883720
H	1.026129	2.756037	-6.099247
H	-1.760166	-0.505268	-5.326185
H	0.323418	0.375321	-6.449300
H	0.442929	3.563328	-3.305598
H	-1.676075	2.084893	-2.945637
H	2.649141	0.408273	-6.619465
H	1.995665	3.023339	-2.668017
H	0.690060	3.463360	-1.564371
H	2.744628	1.982403	-4.679618
H	0.595899	-1.647236	-5.773168
H	-1.449295	-2.521591	-4.739482
H	-5.054214	0.036067	-0.543760
H	-1.319579	1.962046	-1.225178
H	-0.562906	-3.800646	-5.586985
H	4.277412	0.467769	-5.940537
H	-1.527406	0.495222	-2.189138
H	-3.600132	-0.473425	-1.411688
H	3.231556	-0.907195	-5.590950
H	-2.572821	-2.153329	-2.888016
H	-3.458286	0.428719	0.094738
H	-2.520082	-4.480715	-3.810816
H	1.587887	-2.751594	-4.854047
H	0.892648	0.997218	-1.678519
H	-3.443028	-3.277210	-1.867973
H	3.364426	1.214440	-2.400567
H	-5.738914	-1.095120	1.626024
H	-4.675959	-2.364690	-0.196115
H	4.684191	0.866403	-3.522154
H	1.153887	-4.868376	-4.249110
H	3.608727	-0.432223	-3.003013
H	-0.243606	-5.926216	-3.975163
H	-0.872554	0.102896	0.554304
H	-1.794902	-5.039808	-2.291737
H	-4.173621	-0.755945	2.359814
H	-1.997888	0.249377	1.909443
H	4.248697	-2.942753	-3.246695
H	-4.780311	-2.414481	2.304670
H	-4.799401	-4.516698	0.410487
H	5.537466	-1.971457	-2.521795
H	3.173687	-5.941705	-3.893963
H	4.862923	-5.462828	-3.775106
H	-0.263455	0.215298	2.210624
H	-3.579018	-5.321988	-0.582784
H	5.776462	-3.695924	-2.780014
H	4.442742	-7.171076	-3.885465
H	0.130392	-5.706540	-1.505107
H	1.519792	-6.355178	-2.336140
H	-0.129782	-2.144648	1.468407

H	-4.087806	-6.041936	0.940687
H	3.826750	-2.397784	-0.794589
H	-2.341825	-1.764679	3.554141
H	-3.109349	-4.150331	2.213423
H	-1.070632	-5.403513	0.300949
H	-0.610221	-1.628328	3.852354
H	3.378787	-7.126724	-1.673408
H	6.222811	-2.320081	-0.120338
H	6.207998	-5.964834	-1.589397
H	-1.305819	-3.183351	3.390211
H	5.776867	-7.661819	-1.785416
H	-0.715382	-4.774234	1.907056
H	6.336756	-4.073719	-0.247597
H	-1.739930	-6.200956	1.729407
H	1.484369	-3.996477	1.351679
H	5.287843	-3.345642	0.970221
H	3.048518	-3.226284	1.614218
H	1.391037	-6.555918	0.661456
H	5.425203	-6.833596	-0.265229
H	2.912058	-7.450828	0.552672
H	4.189380	-5.426315	1.222525
H	2.461063	-4.452615	2.750246
H	2.337030	-6.867273	2.115995
H	1.626067	-0.765793	0.681267
H	2.587731	-0.378690	-0.586483

Supplementary Table 12. Complex 2A optimised geometry and energy

Energy: -3139.409053 au

C	3.485580	4.155959	6.932255
C	3.806073	3.026314	6.135906
C	3.137239	1.804908	6.419229
C	2.185736	1.747862	7.440248
C	1.878154	2.855159	8.231102
C	2.551507	4.046550	7.965999
B	4.866809	3.124722	4.963839
C	6.028180	2.055117	4.856007
C	6.303957	1.393908	3.628545
C	7.316295	0.434347	3.562099
C	8.106369	0.111293	4.665939
C	7.848093	0.776167	5.863695
C	6.822128	1.717905	5.982511
C	5.520963	1.664046	2.364392
C	9.180945	-0.938047	4.566970
C	6.622642	2.356989	7.338460
C	3.390823	0.537491	5.635807
C	0.835115	2.768789	9.312947
C	4.135285	5.505618	6.726341
C	4.766056	4.293909	3.901404
C	3.539019	4.589653	3.248410
C	3.479600	5.609565	2.296431

C	4.591153	6.387724	1.972274
C	5.788718	6.111059	2.630242
C	5.899968	5.077909	3.564781
C	2.267367	3.818747	3.518423
C	7.256536	4.859283	4.196350
C	4.502748	7.466914	0.926891
C	-2.658003	-1.524991	0.756270
C	-2.499632	-3.049889	0.794230
Si	-3.304789	-3.942959	-0.714022
C	-3.582477	-5.798445	-0.181547
C	-4.431264	-6.646129	-1.138108
C	-2.942800	-3.583053	2.164224
C	-5.085205	-3.270449	-1.117773
C	-5.090032	-1.899102	-1.802674
N	-2.270474	-3.782371	-2.121335
C	-2.871881	-4.250394	-3.368466
C	-1.921748	-5.174883	-4.121661
N	-0.646999	-4.496982	-4.423249
C	-0.770710	-3.646682	-5.621753
C	0.192407	-2.465005	-5.577540
N	-0.004790	-1.679204	-4.364665
Si	0.014929	0.061980	-4.573062
C	1.765014	0.750497	-5.057128
C	2.212903	0.417167	-6.485908
C	-6.046770	-3.289953	0.076748
U	-0.052615	-2.915789	-2.321891
N	0.449664	-1.921156	-0.936141
N	1.466314	-4.770612	-2.428996
C	0.990057	-5.909172	-3.205321
C	0.459496	-5.461021	-4.562959
Si	2.898814	-5.047129	-1.449928
C	2.527302	-5.982137	0.209906
C	1.474504	-5.235828	1.035995
C	3.673269	-3.349895	-0.975383
C	4.423995	-2.682515	-2.133260
C	4.157220	-6.194843	-2.398451
C	4.469984	-5.759596	-3.835398
C	4.550785	-3.358535	0.283338
C	-0.464844	0.886209	-2.901322
C	0.041116	2.323934	-2.716707
C	-1.149366	0.591375	-6.041275
C	-1.227620	2.111175	-6.234508
C	-1.965223	0.816452	-2.587630
C	-2.553723	-0.019403	-5.991713
C	2.823736	0.284557	-4.052747
C	-2.250681	-6.503362	0.093052
C	5.456788	-6.444490	-1.622206
C	2.109839	-7.444797	0.018458
H	-1.792195	2.373573	-7.141721
H	-3.111911	0.180724	-6.918469

H	-1.737803	2.593365	-5.392295
H	-3.141730	0.392302	-5.165023
H	-0.237401	2.572343	-6.322193
H	-2.518408	-1.103087	-5.847152
H	-0.654582	0.181901	-6.936297
H	-0.394522	3.014647	-3.449648
H	-2.550015	1.467814	-3.249818
H	1.525458	0.809128	-7.244257
H	1.129736	2.400448	-2.802046
H	-0.231797	2.700945	-1.720974
H	1.686345	1.846715	-4.984306
H	0.020004	-1.897530	-6.509262
H	-1.788674	-3.248089	-5.643219
H	-6.091910	-1.643885	-2.178612
H	-2.162237	1.145249	-1.558228
H	-0.631009	-4.256286	-6.533478
H	3.206782	0.838116	-6.699007
H	-2.362985	-0.199026	-2.685778
H	-4.395009	-1.868164	-2.646719
H	2.287874	-0.665880	-6.639136
H	-3.153984	-3.412615	-4.031642
H	-4.791679	-1.102856	-1.113332
H	-2.373935	-5.560784	-5.054262
H	1.227653	-2.843307	-5.668973
H	0.044526	0.257721	-2.153596
H	-3.808112	-4.816664	-3.226769
H	2.567084	0.546542	-3.020950
H	-7.066611	-3.005549	-0.222411
H	-5.475540	-3.990773	-1.854348
H	3.810551	0.718030	-4.272798
H	1.273819	-4.956068	-5.092275
H	2.928177	-0.804879	-4.080791
H	0.136251	-6.320346	-5.179826
H	-1.704619	-6.032186	-3.477620
H	-5.733222	-2.581068	0.851985
H	3.840143	-2.694412	-3.058820
H	-6.108769	-4.278780	0.545620
H	-5.411207	-6.200466	-1.341693
H	4.652929	-1.633109	-1.902585
H	3.560812	-5.558381	-4.410354
H	5.068446	-4.843066	-3.856119
H	-3.928667	-6.789843	-2.101970
H	5.380005	-3.182343	-2.336647
H	5.042826	-6.531448	-4.371100
H	0.193959	-6.475473	-2.686114
H	1.769159	-6.661068	-3.418071
H	-1.422709	-3.249093	0.686984
H	-4.609721	-7.650014	-0.724545
H	2.788089	-2.731134	-0.760610
H	-4.016609	-3.434469	2.333742

H	-4.134757	-5.737929	0.769615
H	-1.626070	-6.520696	-0.807699
H	-2.416576	-3.052379	2.969999
H	3.641494	-7.165994	-2.464558
H	4.895901	-2.341946	0.519974
H	6.052401	-5.528160	-1.534003
H	-2.737285	-4.650240	2.292109
H	6.089942	-7.188218	-2.129071
H	-1.666495	-6.002506	0.871280
H	5.447715	-3.978345	0.158202
H	-2.400266	-7.544824	0.414266
H	0.520923	-5.200323	0.498844
H	4.016743	-3.728887	1.163822
H	1.754054	-4.197596	1.239820
H	1.189223	-7.521141	-0.572066
H	5.271862	-6.812009	-0.606673
H	2.878116	-8.036994	-0.491747
H	3.471332	-5.976219	0.776935
H	1.293211	-5.732672	2.000792
H	1.909678	-7.932917	0.983843
H	1.162882	-0.604546	1.050240
H	1.392271	-0.218317	1.651779
H	2.533749	5.801462	1.792597
H	6.668712	6.711552	2.404197
H	7.203275	4.763575	5.285275
H	7.729538	3.944609	3.825849
H	7.922833	5.697792	3.971330
H	5.301814	8.205432	1.042592
H	4.589776	7.044153	-0.081739
H	3.543331	7.992169	0.973003
H	8.457777	0.550115	6.737438
H	7.492417	-0.075707	2.616586
H	7.466852	2.129238	7.996701
H	6.534278	3.446341	7.281450
H	5.710676	1.995159	7.823569
H	9.884808	-0.872908	5.402069
H	8.748253	-1.946049	4.577528
H	9.748106	-0.843620	3.635081
H	2.338886	4.922807	8.576764
H	1.671205	0.806010	7.624039
H	3.918704	6.167573	7.570511
H	5.223478	5.438482	6.631177
H	3.772249	5.991530	5.815465
H	3.343591	0.694790	4.554164
H	4.380067	0.121615	5.849632
H	2.644853	-0.222739	5.883985
H	0.875240	1.805862	9.832400
H	0.959923	3.562081	10.056154
H	-0.174566	2.866909	8.894954
H	5.765604	2.642363	1.939309

H	5.738323	0.904814	1.608297
H	4.439582	1.653888	2.528531
H	-2.082308	-1.057864	1.566209
H	-2.287043	-1.101578	-0.179696
H	-3.705872	-1.226199	0.884623
H	1.863888	4.039177	4.511392
H	1.501978	4.071195	2.779351
H	2.414950	2.735685	3.470968

Supplementary Table 13. Complex 2B (transition state) optimised geometry and energy

Energy: -3139.385500 au

C	1.813751	1.916983	5.104301
C	2.338111	0.997451	4.150674
C	2.030617	-0.375533	4.359146
C	1.215922	-0.775045	5.426238
C	0.679927	0.132068	6.335218
C	1.009826	1.473923	6.159632
B	3.289919	1.471035	2.930608
C	4.770407	0.837445	2.788490
C	5.387885	0.689991	1.515655
C	6.699961	0.212309	1.407096
C	7.460545	-0.136581	2.519932
C	6.866894	0.023743	3.769450
C	5.558397	0.494158	3.923433
C	4.692956	1.035264	0.223253
C	8.859597	-0.675974	2.379522
C	5.084071	0.649881	5.351686
C	2.553697	-1.478164	3.474056
C	-0.218742	-0.313471	7.458391
C	2.105665	3.401931	5.088390
C	3.045448	2.931956	2.273704
C	1.785524	3.298628	1.721992
C	1.582748	4.566940	1.169056
C	2.585226	5.534274	1.140523
C	3.806463	5.196077	1.714121
C	4.054031	3.935372	2.271898
C	0.607242	2.361395	1.709122
C	5.422702	3.760934	2.894773
C	2.348786	6.888615	0.525624
C	-1.436234	-0.546623	2.177983
C	-1.579371	-2.067907	2.054628
Si	-2.592428	-2.589798	0.505393
C	-3.253907	-4.388367	0.857701
C	-4.312317	-4.919418	-0.117784
C	-2.080300	-2.652587	3.383210
C	-4.209292	-1.535311	0.271353
C	-3.974881	-0.121063	-0.270485
N	-1.569799	-2.448151	-0.921883
C	-2.288411	-2.655901	-2.182385
C	-1.747882	-3.877284	-2.915298

N	-0.326627	-3.682719	-3.242710
C	-0.146978	-2.964853	-4.515973
C	1.114752	-2.114341	-4.453384
N	0.998284	-1.124919	-3.381314
Si	1.086012	0.553092	-3.904693
C	2.859348	1.061407	-4.507249
C	3.373251	0.232375	-5.691118
C	-5.110110	-1.500017	1.513445
U	0.746857	-2.076182	-1.237894
N	1.488175	-0.996082	0.023503
N	1.752442	-4.209301	-1.315043
C	0.915996	-5.287711	-1.845850
C	0.419813	-4.949979	-3.243684
Si	3.284407	-4.771022	-0.649964
C	3.045585	-5.870281	0.932383
C	2.171404	-5.154549	1.967542
C	4.337590	-3.240149	-0.163056
C	5.005841	-2.571746	-1.372007
C	4.207370	-5.929214	-1.916215
C	4.349822	-5.373559	-3.337848
C	5.375302	-3.498022	0.938184
C	0.645509	1.673917	-2.409618
C	0.902213	3.166599	-2.657740
C	-0.067975	0.840333	-5.452039
C	0.222449	2.122732	-6.244909
C	-0.767540	1.471937	-1.848967
C	-1.568908	0.746719	-5.151590
C	3.883249	1.037820	-3.369173
C	-2.124539	-5.410291	1.015241
C	5.565577	-6.421000	-1.396777
C	2.507442	-7.284311	0.680088
H	-0.428046	2.195555	-7.129002
H	-2.156761	0.657862	-6.076757
H	0.041427	3.020595	-5.642471
H	-1.925564	1.644052	-4.634440
H	1.257186	2.171619	-6.598013
H	-1.814281	-0.110077	-4.516082
H	0.174043	-0.002961	-6.118095
H	0.199821	3.578222	-3.393347
H	-1.533732	1.852627	-2.533083
H	2.680554	0.230429	-6.540967
H	1.915119	3.366074	-3.022441
H	0.774920	3.741489	-1.731587
H	2.762048	2.103446	-4.850920
H	1.267165	-1.674680	-5.451897
H	-0.999599	-2.295529	-4.660875
H	-4.927257	0.368629	-0.521532
H	-0.887773	2.007476	-0.899468
H	-0.134501	-3.678761	-5.359583
H	4.336602	0.617188	-6.056337

H	-1.003320	0.419838	-1.653012
H	-3.354821	-0.129979	-1.170935
H	3.538645	-0.810685	-5.399083
H	-2.194657	-1.779125	-2.846232
H	-3.467861	0.515206	0.461411
H	-2.325437	-4.107654	-3.829157
H	1.984058	-2.776425	-4.299409
H	1.346793	1.336565	-1.631086
H	-3.374004	-2.798865	-2.054576
H	3.606823	1.706652	-2.548884
H	-6.062837	-0.994141	1.299369
H	-4.768976	-2.078465	-0.506606
H	4.879823	1.341781	-3.721169
H	1.291377	-4.826592	-3.892279
H	3.978500	0.032357	-2.945742
H	-0.197881	-5.761057	-3.669965
H	-1.828766	-4.737878	-2.244657
H	-4.637216	-0.951690	2.336209
H	4.296751	-2.378498	-2.184369
H	-5.350874	-2.502703	1.884678
H	-5.180476	-4.256174	-0.195128
H	5.451433	-1.610788	-1.087816
H	3.405438	-4.970394	-3.714422
H	5.082536	-4.561645	-3.383052
H	-3.907795	-5.047996	-1.128773
H	5.812814	-3.194025	-1.777839
H	4.687656	-6.151476	-4.038082
H	0.046020	-5.487271	-1.198083
H	1.439706	-6.255233	-1.922209
H	-0.570115	-2.470602	1.875186
H	-4.684046	-5.904787	0.199836
H	3.603846	-2.523082	0.235785
H	-3.101642	-2.324024	3.610274
H	-3.746860	-4.289838	1.838018
H	-1.598359	-5.560625	0.066529
H	-1.443869	-2.314716	4.211372
H	3.558184	-6.815828	-1.987817
H	5.905649	-2.571206	1.189171
H	6.286141	-5.597859	-1.325716
H	-2.079547	-3.747027	3.394500
H	6.003640	-7.171837	-2.070689
H	-1.373593	-5.095774	1.747250
H	6.128406	-4.232711	0.627251
H	-2.506776	-6.390695	1.334670
H	1.151358	-5.023281	1.589337
H	4.918967	-3.867458	1.861620
H	2.550322	-4.158269	2.213589
H	1.494085	-7.260206	0.263224
H	5.493483	-6.876913	-0.403225
H	3.134968	-7.858846	-0.010217

H	4.054414	-5.977044	1.360414
H	2.105243	-5.726004	2.904751
H	2.449519	-7.855942	1.617915
H	2.193229	0.033440	1.158369
H	2.547022	0.538541	1.725449
H	0.601668	4.805625	0.759694
H	4.600949	5.941336	1.738912
H	5.380082	3.287618	3.878995
H	6.081067	3.137370	2.283047
H	5.903165	4.737595	3.017026
H	3.185569	7.566851	0.719783
H	2.225942	6.816782	-0.562095
H	1.438562	7.355893	0.919540
H	7.444411	-0.213783	4.662524
H	7.142063	0.124273	0.415486
H	5.930817	0.556333	6.039880
H	4.610634	1.617900	5.535586
H	4.344764	-0.108469	5.624955
H	9.499828	-0.353104	3.207585
H	8.863646	-1.773601	2.375909
H	9.323145	-0.347679	1.443719
H	0.637097	2.207780	6.873784
H	1.007448	-1.836896	5.551009
H	1.834991	3.845846	6.052395
H	3.159920	3.621416	4.904108
H	1.542978	3.925156	4.309565
H	3.598385	-1.322849	3.197168
H	2.474360	-2.441075	3.988286
H	1.986919	-1.550079	2.538793
H	-0.108311	-1.383951	7.658127
H	-0.003174	0.228688	8.385881
H	-1.274470	-0.134021	7.217903
H	5.418156	1.096101	-0.593671
H	3.947541	0.279729	-0.048384
H	4.165386	1.989428	0.282944
H	-0.745973	-0.288513	2.989716
H	-1.042950	-0.099796	1.261864
H	-2.399809	-0.075630	2.406629
H	-0.316015	2.909415	1.496241
H	0.718706	1.581439	0.949002
H	0.486866	1.846797	2.664241

Supplementary Table 14. Complex 2C optimised geometry and energy

Energy: -2065.511861 au

C	-1.082711	-0.849393	1.802816
C	-1.347889	-2.356604	1.684952
C	-1.720349	-2.922940	3.064329
Si	-2.584317	-2.819726	0.279514
N	-1.706532	-2.777504	-1.276277
U	0.519134	-2.638405	-1.731555

N	1.338543	-4.742299	-1.951387
Si	2.880063	-5.362912	-1.291822
C	3.520598	-6.853862	-2.347316
C	4.861720	-7.393951	-1.825930
C	-3.291074	-4.573757	0.693950
C	-2.209568	-5.658113	0.714348
C	-4.125183	-1.651632	0.197992
C	-4.863968	-1.559100	1.541114
C	-4.477269	-5.028473	-0.166510
C	-3.862727	-0.256582	-0.378414
C	-2.573642	-2.883054	-2.462005
C	-2.171479	-4.081258	-3.312346
N	-0.759544	-3.963545	-3.724692
C	-0.106482	-5.279925	-3.856338
C	0.425699	-5.748538	-2.509286
C	-0.596476	-3.155444	-4.949726
C	0.733195	-2.413853	-4.899955
N	0.778044	-1.557519	-3.705905
Si	1.095915	0.174669	-4.003510
C	-0.001408	0.789576	-5.478720
C	-1.510466	0.765206	-5.203142
C	2.933018	0.482701	-4.512793
C	3.911842	0.183886	-3.371799
C	0.801725	1.115402	-2.355412
C	-0.617698	1.002571	-1.790117
C	3.349661	-0.269227	-5.783263
N	1.474748	-1.700902	-0.345297
C	2.643866	-6.037370	0.501779
C	1.857667	-7.352579	0.569161
C	4.125867	-3.895142	-1.234476
C	5.208106	-4.029599	-0.152643
C	2.000209	-4.988800	1.414511
C	4.777938	-3.601324	-2.592216
C	3.588883	-6.629784	-3.862883
C	1.232290	2.587890	-2.408073
C	0.414096	2.157514	-6.039324
H	-0.182302	2.412919	-6.925620
H	-2.084292	0.880596	-6.132316
H	0.258538	2.960352	-5.310029
H	-1.808484	1.586898	-4.543244
H	1.466460	2.184911	-6.337671
H	-1.833126	-0.166181	-4.726273
H	0.189071	0.056007	-6.277288
H	0.575645	3.176849	-3.058312
H	-1.340456	1.558464	-2.396016
H	2.692883	-0.056365	-6.634363
H	2.256234	2.715621	-2.772808
H	1.181693	3.043536	-1.410277
H	2.993737	1.560360	-4.730626
H	0.846266	-1.854884	-5.839415

H	-1.402051	-2.416713	-4.983877
H	-4.801564	0.303749	-0.485128
H	-0.667682	1.414943	-0.774393
H	-0.686904	-3.790757	-5.846728
H	4.370275	0.001603	-6.084894
H	-0.980524	-0.031602	-1.734642
H	-3.392940	-0.302750	-1.364949
H	3.342070	-1.353055	-5.622192
H	-2.518599	-1.969573	-3.075899
H	-3.206403	0.335486	0.266683
H	-2.823875	-4.198774	-4.193857
H	1.557850	-3.144399	-4.898474
H	1.469461	0.598339	-1.649583
H	-3.637943	-2.995536	-2.208607
H	3.722151	0.799712	-2.486566
H	-5.802363	-0.998948	1.433755
H	-4.809098	-2.157827	-0.500913
H	4.949258	0.368349	-3.682444
H	0.741241	-5.165717	-4.538060
H	3.847020	-0.863977	-3.060931
H	-0.798314	-6.012578	-4.304349
H	-2.272178	-4.984661	-2.703169
H	-4.266223	-1.035789	2.295867
H	4.042957	-3.495354	-3.397122
H	-5.118679	-2.543590	1.948126
H	-5.308738	-4.316820	-0.146601
H	5.361671	-2.672925	-2.553349
H	2.651656	-6.237459	-4.267996
H	4.377432	-5.921496	-4.132610
H	-4.189914	-5.170920	-1.215051
H	5.471097	-4.399316	-2.881079
H	3.807590	-7.569835	-4.386982
H	-0.420649	-5.947748	-1.833935
H	0.906251	-6.724762	-2.665098
H	-0.395615	-2.823277	1.386491
H	-4.868443	-5.992737	0.185215
H	3.507794	-3.023997	-0.962206
H	-2.673198	-2.518992	3.425016
H	-3.665780	-4.461429	1.723241
H	-1.789363	-5.809141	-0.285937
H	-0.958265	-2.651324	3.806580
H	2.777744	-7.647365	-2.172977
H	5.864946	-3.149595	-0.153507
H	5.671962	-6.670816	-1.972038
H	-1.803811	-4.013522	3.071424
H	5.149677	-8.307835	-2.362475
H	-1.374565	-5.410980	1.377549
H	5.846856	-4.904204	-0.320403
H	-2.616416	-6.623002	1.045322
H	0.976385	-4.771729	1.088090

H	4.786967	-4.119779	0.852852
H	2.543389	-4.038119	1.415038
H	0.845801	-7.236156	0.163268
H	4.829938	-7.640693	-0.759318
H	2.345503	-8.161820	0.016015
H	3.658979	-6.234276	0.879251
H	1.942004	-5.342679	2.452639
H	1.746727	-7.691741	1.607914
H	1.929680	-1.208580	0.421213
H	-0.280690	-0.654801	2.527597
H	-0.777285	-0.406574	0.851720
H	-1.969721	-0.313353	2.159208

Supplementary Table 15. Complex 2D$_1$ (transition state) optimised geometry and energy

Energy: -3139.367779 au

U	0.753146	-2.028535	-1.187166
N	1.588435	-0.539797	0.057233
B	2.949058	0.982688	2.365189
C	2.079699	0.608083	3.701378
C	1.647158	1.564340	4.665293
C	0.917771	1.173405	5.796384
C	0.592332	-0.154300	6.058169
C	1.049228	-1.103334	5.146082
C	1.768360	-0.746318	3.999273
C	2.671377	2.480300	1.745908
C	3.661578	3.497938	1.601118
C	3.355723	4.731093	1.009217
C	2.080149	5.053534	0.556362
C	1.085163	4.101858	0.767139
C	1.352283	2.859456	1.355087
C	5.075602	3.381183	2.128911
C	0.157116	1.990366	1.647016
C	1.788724	6.370929	-0.112767
C	4.506971	0.449871	2.347487
C	5.221752	0.355629	1.116749
C	6.561179	-0.047320	1.068688
C	7.275547	-0.396776	2.212380
C	6.598642	-0.295050	3.422328
C	5.262590	0.123492	3.514456
C	4.597320	0.729919	-0.200607
C	8.705542	-0.863900	2.139939
C	4.747880	0.263929	4.931035
C	1.992830	3.036176	4.596438
C	-0.216760	-0.546147	7.266737
C	2.242070	-1.872646	3.120368
N	-1.533324	-2.417083	-0.879866
C	-2.215652	-2.506455	-2.182434
C	-1.754101	-3.737735	-2.949633
N	-0.314602	-3.634750	-3.222306
C	-0.025688	-2.950680	-4.488926

C	1.244702	-2.127726	-4.333892
N	1.058590	-1.086956	-3.308591
Si	1.109505	0.533237	-4.022127
C	0.663827	1.840146	-2.689144
C	0.735694	3.287406	-3.197011
N	1.704220	-4.147204	-1.199117
C	0.799094	-5.197088	-1.696095
C	0.372868	-4.928305	-3.130401
Si	-2.652012	-2.686329	0.468773
C	-4.205786	-1.535741	0.264907
C	-3.890278	-0.079178	-0.094157
C	-1.714079	-2.387556	2.118649
C	-2.227641	-3.180913	3.328328
C	-1.656480	-0.895051	2.466047
C	-3.435184	-4.468206	0.573316
C	-4.514860	-4.809606	-0.461783
Si	3.245624	-4.827540	-0.651028
C	4.377533	-3.390159	-0.068689
C	5.119426	-2.714143	-1.230454
C	4.071614	-5.905191	-2.046605
C	4.275113	-5.230400	-3.408337
C	3.007491	-6.066458	0.826935
C	2.173311	-5.435941	1.944995
C	5.383904	-6.556409	-1.584475
C	-2.411103	-5.605468	0.650338
C	-5.183349	-1.609073	1.446762
C	-0.083751	0.625563	-5.567001
C	0.214596	1.772832	-6.543548
C	2.855178	1.007952	-4.716529
C	3.386781	0.106788	-5.840199
C	-1.576317	0.629285	-5.214664
C	3.907113	1.129586	-3.611637
C	-0.676512	1.605481	-1.981429
C	2.460892	-7.460125	0.488554
C	5.378450	-3.775267	1.030901
H	-0.460851	1.734122	-7.410696
H	-2.196691	0.446221	-6.103806
H	0.071480	2.751934	-6.073366
H	-1.884296	1.599145	-4.808501
H	1.238110	1.744279	-6.928881
H	-1.832658	-0.129610	-4.468526
H	0.111842	-0.309946	-6.114244
H	-0.066733	3.503218	-3.912847
H	-1.523282	1.755618	-2.658577
H	2.668573	-0.030952	-6.657042
H	1.687996	3.514734	-3.687268
H	0.624168	3.988568	-2.360858
H	2.705406	2.010075	-5.148291
H	1.511169	-1.727136	-5.322835
H	-0.849493	-2.266841	-4.713259

H	-4.807833	0.469265	-0.351685
H	-0.796201	2.305057	-1.146831
H	0.041324	-3.675801	-5.319976
H	4.302240	0.528358	-6.279401
H	-0.775168	0.595835	-1.565509
H	-3.209331	-0.010431	-0.947466
H	3.648684	-0.887344	-5.460992
H	-2.011599	-1.615935	-2.799207
H	-3.418921	0.451932	0.737897
H	-2.321713	-3.883244	-3.886077
H	2.075964	-2.802226	-4.071554
H	1.456144	1.737353	-1.932598
H	-3.312720	-2.545227	-2.095945
H	3.633553	1.868209	-2.852649
H	-6.092504	-1.025239	1.243716
H	-4.735676	-1.961520	-0.601567
H	4.883090	1.427196	-4.020698
H	1.271241	-4.885760	-3.752239
H	4.046171	0.174150	-3.095547
H	-0.262875	-5.740234	-3.527175
H	-1.924703	-4.616127	-2.320669
H	-4.741399	-1.200860	2.362307
H	4.447199	-2.397916	-2.035775
H	-5.499499	-2.635164	1.666119
H	-5.335452	-4.085037	-0.466827
H	5.650540	-1.825959	-0.874403
H	3.380364	-4.699703	-3.745692
H	5.087487	-4.498154	-3.379972
H	-4.108885	-4.850873	-1.479309
H	5.866323	-3.386577	-1.668155
H	4.535496	-5.969662	-4.179536
H	-0.098728	-5.272614	-1.065441
H	1.249386	-6.201495	-1.678619
H	-0.681129	-2.719461	1.926417
H	-4.955850	-5.796482	-0.258970
H	3.699185	-2.638235	0.363703
H	-3.270810	-2.932630	3.559537
H	-3.940140	-4.425526	1.551748
H	-1.962482	-5.799682	-0.329788
H	-1.635453	-2.935079	4.218871
H	3.350041	-6.722348	-2.204895
H	5.963288	-2.898354	1.332357
H	6.162132	-5.804767	-1.409167
H	-2.172175	-4.264435	3.185542
H	5.773677	-7.247946	-2.345425
H	-1.588229	-5.392267	1.340609
H	6.084117	-4.543118	0.688798
H	-2.880016	-6.545166	0.975537
H	1.154222	-5.223379	1.601370
H	4.885993	-4.158920	1.929417

H	2.598687	-4.489723	2.288416
H	1.410993	-7.417953	0.177912
H	5.267855	-7.125711	-0.656338
H	3.021978	-7.958069	-0.309737
H	4.028085	-6.209103	1.214516
H	2.097145	-6.101198	2.816792
H	2.499577	-8.116358	1.369968
H	0.056907	4.338856	0.492599
H	4.148606	5.474312	0.920193
H	5.114982	2.880664	3.098482
H	5.731457	2.807493	1.466784
H	5.511321	4.380195	2.243734
H	2.461065	7.158887	0.244025
H	1.915231	6.307881	-1.201879
H	0.759265	6.696532	0.072993
H	7.134275	-0.525640	4.343448
H	7.064381	-0.071342	0.101921
H	5.579717	0.174831	5.638616
H	4.267926	1.230259	5.100990
H	4.005191	-0.495116	5.192102
H	9.241805	-0.659416	3.072916
H	8.768674	-1.945592	1.960799
H	9.247582	-0.372081	1.324569
H	0.612682	1.939606	6.509621
H	0.851683	-2.158998	5.333349
H	1.790797	3.514241	5.561588
H	3.046797	3.199256	4.356297
H	1.421549	3.571259	3.831894
H	3.330493	-1.854530	3.000481
H	1.956378	-2.836805	3.551033
H	1.820368	-1.793083	2.114077
H	-1.286008	-0.624111	7.028650
H	0.095471	-1.519628	7.661087
H	-0.117619	0.190740	8.071052
H	5.338690	0.687019	-1.004458
H	3.772096	0.056112	-0.448524
H	4.178963	1.740169	-0.179109
H	-0.957895	-0.708438	3.288186
H	-1.327996	-0.292618	1.617436
H	-2.640610	-0.525767	2.778095
H	-0.070185	1.984873	2.718485
H	-0.731772	2.357408	1.123311
H	0.329438	0.951162	1.364491
H	2.311866	0.113619	1.306038
H	1.545689	0.407952	-0.338134

Supplementary Table 16. Complex 2D$_2$ (transition state) optimised geometry and energy

Energy: -3139.915412 au

U	0.852959	-2.118783	-1.148573
N	1.317322	-0.759900	0.267195

H	2.093538	-0.127658	1.299459
B	3.151912	1.246195	3.062138
C	4.703090	0.875859	2.730561
C	5.193955	0.943582	1.397491
C	6.524875	0.633210	1.095618
C	7.438468	0.251541	2.075318
C	6.976180	0.218747	3.388185
C	5.652492	0.522126	3.731374
N	-1.293226	-2.613720	-0.767115
Si	-2.279660	-3.022976	0.683971
C	-3.055633	-4.800217	0.617171
C	-2.044273	-5.937344	0.434538
N	1.043745	-0.937396	-3.045184
Si	1.124579	0.806229	-3.524224
C	2.772566	1.193194	-4.464284
C	4.005919	0.708263	-3.696374
N	1.815881	-4.138228	-1.333370
Si	3.420316	-4.627112	-0.685479
C	3.233398	-5.880442	0.767333
C	2.859017	-7.314212	0.369159
C	2.464035	0.584118	4.377949
C	2.012492	1.362154	5.478966
C	1.405768	0.753124	6.584777
C	1.225464	-0.623395	6.679332
C	1.716733	-1.396779	5.628796
C	2.324285	-0.823688	4.506366
C	2.595749	2.712210	2.617362
C	3.429326	3.861447	2.509503
C	2.901189	5.094677	2.109454
C	1.553865	5.272736	1.805316
C	0.722401	4.165732	1.960527
C	1.210618	2.920051	2.372706
C	2.196594	2.860733	5.581051
C	0.541114	-1.253704	7.862909
C	2.836974	-1.780956	3.459331
C	4.900886	3.869761	2.859794
C	1.021838	6.600532	1.334691
C	0.172462	1.856266	2.627075
C	4.333260	1.384525	0.239671
C	8.858100	-0.113122	1.731642
C	5.345014	0.499797	5.212449
C	-2.103961	-2.670621	-1.999826
C	-1.596412	-3.788773	-2.893152
N	-0.179532	-3.536204	-3.215452
C	0.593812	-4.782468	-3.351678
C	1.093102	-5.237188	-1.990598
C	-0.037130	-2.680949	-4.408952
C	1.179723	-1.788578	-4.248547
C	1.074226	1.900119	-1.949683
C	-0.332654	2.060394	-1.356031

C	-0.299535	1.202595	-4.776378
C	-1.725067	0.874492	-4.317913
C	1.725661	3.282105	-2.114204
C	-3.792898	-1.820785	0.751324
C	-4.672690	-2.033159	1.992544
C	-1.153710	-2.939516	2.240876
C	-1.065517	-1.521495	2.815635
C	-3.455121	-0.338679	0.555870
C	-1.532250	-3.917229	3.364558
C	-4.254452	-5.010355	-0.318168
C	4.225351	-3.018519	-0.024891
C	5.356895	-3.251963	0.987800
C	4.447451	-5.519337	-2.059071
C	5.842571	-5.935523	-1.567018
C	4.706671	-2.097666	-1.154501
C	4.551540	-4.798707	-3.408551
C	2.272710	-5.356358	1.839174
C	-0.234878	2.654686	-5.276761
C	2.876541	0.784621	-5.940927
H	-0.960351	2.823096	-6.083604
H	-2.432999	0.949732	-5.153957
H	-0.479056	3.361613	-4.476210
H	-2.070238	1.565885	-3.543742
H	0.751729	2.927183	-5.664521
H	-1.808052	-0.135479	-3.905759
H	-0.084504	0.556247	-5.641897
H	1.223741	3.887962	-2.877452
H	-0.954155	2.715981	-1.975919
H	2.007697	1.089051	-6.533456
H	2.783275	3.215321	-2.385921
H	1.670482	3.835138	-1.169128
H	2.785131	2.293727	-4.443018
H	1.288953	-1.201200	-5.169022
H	-0.922338	-2.045054	-4.488942
H	-4.368942	0.256809	0.430466
H	-0.277239	2.519829	-0.362223
H	0.013701	-3.295550	-5.322089
H	3.760053	1.244317	-6.403218
H	-0.871476	1.109859	-1.259362
H	-2.832397	-0.178877	-0.330117
H	2.995944	-0.298548	-6.057559
H	-2.061714	-1.715946	-2.544183
H	-2.917182	0.073674	1.414158
H	-2.191758	-3.897962	-3.814082
H	2.090160	-2.406452	-4.195794
H	1.680288	1.348737	-1.218646
H	-3.170938	-2.834350	-1.797580
H	3.983146	1.000386	-2.642946
H	-5.584438	-1.424374	1.933052
H	-4.402486	-2.118564	-0.116194

H	4.929911	1.109868	-4.132754
H	1.459388	-4.575262	-3.986563
H	4.082037	-0.384145	-3.727868
H	-0.003992	-5.560433	-3.853562
H	-1.663119	-4.728285	-2.339438
H	-4.155659	-1.741512	2.912899
H	3.931178	-1.885882	-1.903650
H	-4.987443	-3.075809	2.109529
H	-5.037182	-4.258083	-0.179947
H	5.046250	-1.138105	-0.753901
H	3.579011	-4.451474	-3.770209
H	5.204835	-3.923012	-3.351396
H	-3.959737	-4.991142	-1.373853
H	5.550652	-2.543974	-1.690782
H	4.972601	-5.462869	-4.174627
H	0.249888	-5.577865	-1.374356
H	1.720213	-6.126082	-2.149392
H	-0.146116	-3.218641	1.890883
H	-4.715134	-5.991585	-0.141881
H	3.430515	-2.483725	0.523579
H	-2.545372	-3.727116	3.737930
H	-3.443536	-4.883281	1.644847
H	-1.708058	-6.005708	-0.605963
H	-0.851714	-3.790112	4.215415
H	3.887033	-6.450361	-2.237048
H	5.755432	-2.293997	1.340055
H	6.488487	-5.063909	-1.414226
H	-1.483782	-4.967406	3.062503
H	6.341328	-6.576118	-2.306011
H	-1.151080	-5.817862	1.055694
H	6.188883	-3.809135	0.542930
H	-2.491040	-6.908666	0.684433
H	1.251323	-5.286762	1.447282
H	5.017982	-3.808536	1.866434
H	2.552326	-4.362860	2.202704
H	1.851219	-7.367428	-0.058581
H	5.810871	-6.491301	-0.623921
H	3.552513	-7.746824	-0.359243
H	4.240320	-5.916863	1.210673
H	2.242343	-6.025861	2.708737
H	2.862701	-7.971861	1.248343
H	-0.347439	4.276639	1.784541
H	3.571516	5.950927	2.046270
H	5.118784	3.297513	3.763823
H	5.522167	3.438916	2.068647
H	5.238766	4.897942	3.023928
H	-0.025009	6.738385	1.623458
H	1.600113	7.432546	1.748470
H	1.070234	6.686004	0.241347
H	7.673521	-0.035632	4.185436

H	6.861172	0.715433	0.062537
H	6.275826	0.449963	5.786223
H	4.801460	1.389749	5.537931
H	4.729301	-0.357490	5.498799
H	9.156567	0.305571	0.765783
H	9.560725	0.247176	2.489988
H	8.985978	-1.201721	1.668315
H	1.077511	1.383401	7.410149
H	1.642335	-2.482709	5.687349
H	2.053975	3.184389	6.616924
H	3.194022	3.179965	5.269711
H	1.488209	3.415158	4.958436
H	3.800642	-1.464370	3.054510
H	2.956110	-2.780848	3.888799
H	2.140272	-1.859285	2.615625
H	1.058768	-2.161917	8.190122
H	0.495867	-0.566081	8.712511
H	-0.489587	-1.541994	7.619681
H	4.954849	1.577116	-0.640095
H	3.593519	0.623452	-0.030801
H	3.774739	2.294623	0.467412
H	-0.313847	-1.463905	3.610671
H	-0.792387	-0.785956	2.058039
H	-2.021624	-1.212745	3.251202
H	-0.092008	1.816930	3.690061
H	-0.747164	2.063643	2.069261
H	0.522187	0.857424	2.372772
H	0.582681	-0.035044	0.246343
H	2.506167	0.380119	1.967066

Supplementary Table 17. Complex 2A in U(VI) profile optimised geometry and energy

Energy: -2063.783166 a.u

U -0.271873 -3.311613 -3.002768
Si -3.474868 -3.670906 -1.189924
Si 0.264108 -0.549446 -5.336494
Si 2.301505 -5.739668 -2.052863
N 0.349581 -2.429736 -1.642186
N -2.477234 -3.721300 -2.680523
N -0.017102 -2.293842 -5.017411
N -1.189223 -4.842396 -4.946755
N 0.830289 -5.296720 -2.990485
C 4.726615 -7.274934 -2.436135
C 4.218293 -4.314292 -0.382405
C -2.240949 -1.535252 0.350053
C -5.923554 -2.414349 -0.287643
C -2.542424 -0.238142 -6.133595
C -1.081915 0.587185 -3.029204
C 3.126632 -0.871153 -5.457299
C 1.078232 1.697073 -3.674750
C 0.305681 0.369320 -3.647458

C 2.027320 -0.613004 -7.705220
C 1.956267 -0.239994 -6.218375
C -3.032176 -6.463171 -0.567217
C -4.726174 -1.204077 -2.156428
C -5.027219 -2.568866 -1.525176
C -5.273505 -5.993564 -1.607588
C -4.150872 -5.428324 -0.727090
C -2.900275 -3.571843 1.658536
C -2.409290 -3.059956 0.294452
C 3.897190 -3.415727 -2.712328
C 3.222716 -4.135330 -1.538026
C -2.565211 -5.247828 -4.583506
C -3.260637 -4.104778 -3.863781
C -0.965012 1.602438 -6.833600
C -1.103618 0.101561 -6.538632
C -0.020216 -3.105571 -6.238842
C -1.193305 -4.069639 -6.207516
C -0.289347 -6.011069 -5.048338
C 0.199231 -6.432167 -3.672714
C 3.639106 -6.477186 -4.563795
C 3.394768 -6.933166 -3.120203
C 0.954880 -5.863197 0.483631
C 1.855385 -6.694714 -0.436733
C 1.259248 -8.091793 -0.649103
H -3.190111 -1.037584 0.578548
H -1.852381 -1.125354 -0.584695
H -1.531163 -1.258429 1.139897
H 1.102612 -8.599525 0.312132
H 0.781183 -6.375979 1.439279
H 2.822304 -6.826965 0.073339
H 1.905381 -8.737502 -1.253068
H 4.592994 -7.634377 -1.410311
H 0.281701 -8.044702 -1.142871
H 1.374780 -4.877738 0.708612
H 3.748104 -4.723822 0.516688
H -0.024314 -5.697775 0.021601
H -3.424486 -7.425106 -0.210954
H 5.051330 -4.974162 -0.648076
H -2.263552 -6.141386 0.142141
H 5.260121 -8.059481 -2.989360
H -2.912221 -4.663596 1.727444
H 5.391653 -6.405225 -2.396994
H 4.654216 -3.345574 -0.104878
H 2.822272 -7.872184 -3.170117
H -2.244249 -3.204178 2.458138
H -2.528377 -6.651039 -1.522592
H -4.587045 -5.261363 0.269860
H -3.911508 -3.216257 1.887886
H 2.421302 -3.476998 -1.176381
H -5.700217 -6.900733 -1.158738

H -1.409049 -3.483179 0.121475
H 0.884266 -7.279323 -3.820834
H -0.641453 -6.840966 -3.089612
H 4.165374 -7.252388 -5.136972
H 4.779009 -3.960708 -3.068920
H -4.908497 -6.281585 -2.600435
H 4.254995 -5.573318 -4.603706
H 2.706639 -6.253235 -5.090570
H 4.237383 -2.415684 -2.414212
H -6.096612 -5.285752 -1.749603
H -6.214728 -3.378400 0.143700
H 3.223582 -3.289363 -3.566552
H -5.424569 -1.840002 0.500567
H -2.501816 -6.099439 -3.900369
H -0.797051 -6.833198 -5.578544
H 3.081543 -1.964521 -5.509818
H 0.577512 -5.712434 -5.644569
H 4.090929 -0.565734 -5.884700
H -5.607678 -3.138265 -2.267793
H -6.848492 -1.877333 -0.537262
H 3.136147 -0.598145 -4.396978
H -4.279806 -4.434998 -3.621273
H 0.852316 -0.308655 -2.975780
H 0.924180 -3.661130 -6.357221
H -3.111126 -5.574375 -5.483496
H -4.203093 -0.540275 -1.461361
H -3.393715 -3.255073 -4.552339
H 1.978810 -1.697400 -7.853544
H -4.102567 -1.295258 -3.051116
H -1.687924 -0.325665 -2.999417
H 2.977255 -0.278258 -8.142899
H -1.195511 -4.755022 -7.070302
H -0.992857 0.947731 -1.997113
H -5.655211 -0.696738 -2.449480
H -2.118014 -3.487863 -6.242940
H -0.118343 -2.506308 -7.156059
H 2.070869 0.853538 -6.157136
H 1.081893 2.154797 -2.677112
H 2.122786 1.568631 -3.974513
H 1.222888 -0.160194 -8.294220
H -1.655994 1.336458 -3.586214
H 0.625770 2.424081 -4.358766
H -0.904857 -0.427089 -7.484141
H -2.658246 -1.298675 -5.888992
H 0.038281 1.872697 -7.180036
H -2.860828 0.331325 -5.255021
H -1.179464 2.206428 -5.944681
H -3.246260 -0.002221 -6.942876
H -1.673658 1.917361 -7.611129

Supplementary Table 18. Complex 2B in U(VI) profile optimised geometry and energy

Energy: -3137.610809 a.u

U 0.739767 -1.964585 -1.157237
Si 3.179885 -4.664260 -0.488603
Si 1.243889 0.509298 -3.875787
Si -2.505494 -2.523096 0.629004
N 1.603646 -4.013680 -1.101984
N 1.525230 -0.916288 0.101311
N 1.123792 -1.169577 -3.215117
N -0.364798 -3.557583 -3.091078
N -1.447493 -2.275828 -0.819946
C 2.372595 -7.225520 0.775408
C 5.322151 -6.383811 -1.331591
C -2.501060 -5.467141 0.671181
C 4.076814 0.952503 -3.483283
C -1.460023 0.734504 -5.031021
C -0.470026 1.483299 -1.755389
C 0.375122 1.881771 -6.295648
C 0.032369 0.691233 -5.384738
C 1.038176 3.185326 -2.821002
C 0.885591 1.706310 -2.433813
C 5.302360 -3.522519 1.126184
C 4.178295 -5.197981 -3.233001
C 3.992954 -5.795945 -1.833462
C 4.993640 -2.530990 -1.166217
C 4.282952 -3.185777 0.026540
C 1.990506 -5.115806 2.092658
C 2.873654 -5.800080 1.044601
C 0.271704 -4.881979 -2.967709
C 0.710251 -5.106342 -1.532914
C -4.815129 -1.246666 1.816186
C 3.475207 -0.010434 -5.724220
C 3.002090 0.884825 -4.569952
C 1.255002 -2.187070 -4.272477
C -0.059033 -2.935840 -4.392657
C -1.815510 -3.605108 -2.827637
C -2.215616 -2.346980 -2.075963
C -3.490854 0.217667 0.238373
C -3.926353 -1.215505 0.562340
C -1.951595 -3.161459 3.415102
C -4.580514 -4.438036 -0.299168
C -3.413110 -4.235221 0.676395
C -1.404236 -2.405903 2.193775
C -1.108755 -0.950886 2.574837
C 1.818109 6.708769 0.271164
C 5.212924 3.762320 2.413802
C 0.378715 2.152252 1.741645
C 3.791402 3.858481 1.904292
C 3.440193 5.095272 1.348117
C 2.157246 5.377359 0.887386

C 1.203221 4.372840 1.032132
C 1.517353 3.124864 1.582437
C 2.834544 2.809254 2.013659
C 2.091282 3.342932 4.866732
C 0.244789 -0.361647 7.639588
C 2.647555 -1.567794 3.429330
C 5.130628 0.774933 5.093730
C 8.866230 -0.522301 2.066860
C 4.581729 0.977159 -0.019831
C 5.550173 0.558406 3.656385
C 6.873795 0.134186 3.480081
C 7.445970 -0.047278 2.224206
C 6.643286 0.237417 1.121898
C 5.314100 0.655864 1.257750
C 4.710494 0.812859 2.534568
C 1.212421 1.416513 6.123016
C 0.986669 0.072107 6.403333
C 1.479396 -0.852361 5.485353
C 2.142696 -0.458458 4.317398
C 2.349261 0.912436 4.004998
C 1.876893 1.848701 4.967851
B 3.162843 1.335152 2.649874
H 0.331296 1.751631 2.757077
H 0.487046 1.293423 1.072531
H -0.579145 2.633847 1.519204
H -2.016497 -0.442092 2.916481
H -0.701315 -0.379743 1.738609
H -0.379926 -0.896001 3.390252
H 4.050976 1.930207 0.046651
H 3.835159 0.215403 -0.270067
H 5.285598 1.033566 -0.854759
H -0.830036 -0.468587 7.443698
H 0.354303 0.367031 8.448961
H 0.603930 -1.329332 8.005122
H 2.014155 -1.692554 2.543178
H 2.648185 -2.517102 3.973917
H 3.662187 -1.375901 3.072725
H 1.441732 3.809502 4.120209
H 3.115147 3.598634 4.584159
H 1.883120 3.816396 5.831630
H 1.360912 -1.916668 5.688267
H 0.875311 2.165593 6.838557
H 9.501704 -0.162128 2.882321
H 9.301274 -0.179569 1.122591
H 8.925264 -1.618372 2.072515
H 4.448085 -0.000602 5.452626
H 4.614725 1.727086 5.237541
H 6.011420 0.771831 5.743835
H 7.069620 0.157834 0.122005
H 7.484793 -0.039778 4.365097

H 2.371450 7.523972 0.748749
H 0.749576 6.930163 0.357702
H 2.070134 6.731993 -0.796970
H 5.648420 4.763584 2.493984
H 5.855502 3.172145 1.753506
H 5.272208 3.292940 3.398103
H 4.201022 5.873100 1.292405
H 0.173798 4.571482 0.734163
H 2.521749 0.476379 1.674159
H 2.083690 -0.140901 0.981455
H 2.391760 -7.817765 1.699682
H 1.944672 -5.703386 3.018875
H 3.882434 -5.894279 1.475280
H 2.979494 -7.760602 0.037802
H 5.227080 -6.872863 -0.356872
H 1.334957 -7.234186 0.423229
H 2.351670 -4.117697 2.354178
H 4.822720 -3.866255 2.047009
H 0.962129 -5.004578 1.729015
H -3.041058 -6.358778 1.015360
H 6.009293 -4.298347 0.809219
H -1.620840 -5.351090 1.311825
H 5.708290 -7.132442 -2.035746
H -2.070354 -4.235082 3.241263
H 6.090906 -5.609344 -1.235671
H 5.885277 -2.629735 1.380182
H 3.293189 -6.639697 -1.936312
H -1.270995 -3.039703 4.266220
H -2.142694 -5.692524 -0.339358
H -3.857109 -4.178147 1.683370
H -2.925297 -2.767581 3.727924
H 3.592297 -2.441144 0.448055
H -5.118605 -5.367809 -0.071311
H -0.448021 -2.881968 1.920075
H 1.193600 -6.090697 -1.481361
H -0.168423 -5.173741 -0.878817
H 4.490756 -5.970762 -3.947783
H 5.749535 -3.198366 -1.593976
H -4.241224 -4.521749 -1.338321
H 4.947290 -4.420391 -3.245444
H 3.259198 -4.747302 -3.619174
H 5.509712 -1.620230 -0.846086
H -5.312193 -3.625593 -0.255487
H -5.216633 -2.244126 2.025301
H 4.304463 -2.255648 -1.973334
H -4.270774 -0.916295 2.707259
H -2.025263 -4.468753 -2.191649
H -0.407127 -5.672904 -3.322848
H 4.205359 -0.014348 -2.985926
H 1.157393 -4.898944 -3.607128

H 5.049159 1.229232 -3.911748
H -4.558847 -1.546521 -0.275981
H -5.672071 -0.571135 1.697154
H 3.841094 1.688378 -2.710390
H -3.301470 -2.353920 -1.911509
H 1.662998 1.481031 -1.688938
H 2.069365 -2.889430 -4.045792
H -2.376356 -3.735245 -3.765998
H -2.877609 0.650587 1.033543
H -2.025229 -1.465365 -2.707019
H 3.710716 -1.021812 -5.374430
H -2.910165 0.268698 -0.686815
H -0.615670 0.456607 -1.386147
H 4.395788 0.390123 -6.168116
H -0.046719 -3.694622 -5.190998
H -0.576754 2.134376 -0.882588
H -4.367678 0.866940 0.114857
H -0.846806 -2.215489 -4.629250
H 1.506678 -1.756824 -5.249554
H 2.883202 1.901139 -4.977531
H 0.977774 3.817981 -1.928030
H 1.998012 3.396589 -3.302866
H 2.740601 -0.099562 -6.532814
H -1.306139 1.692742 -2.428569
H 0.244685 3.506320 -3.506020
H 0.195145 -0.217862 -5.984522
H -1.757906 -0.060895 -4.339972
H 1.388432 1.825051 -6.702640
H -1.730499 1.687643 -4.564561
H 0.286081 2.836663 -5.766613
H -2.078657 0.636395 -5.932833
H -0.314923 1.923654 -7.148864

Supplementary Table 19. Complex 2C in U(VI) profile optimised geometry and energy
Energy: -3137.612842 a.u
U -0.851018 -1.093306 -1.354912
Si -0.355230 1.459182 -3.900508
Si -3.960460 -1.601695 0.459569
Si 1.709792 -3.624807 -0.588184
N -0.011415 -0.011859 -0.086406
N -3.000723 -1.482308 -1.086169
N -1.846368 -2.723008 -3.302281
N -0.465536 -0.282192 -3.367165
N 0.101014 -3.071275 -1.271012
C 0.873702 2.053744 4.126274
C 0.374463 2.947580 5.115346
C -0.381921 2.483068 6.199980
C -0.675354 1.135321 6.391038
C -0.157007 0.244495 5.454401
C 0.591616 0.677932 4.352606

C 1.490036 4.039933 2.247713
C 2.474436 5.067281 2.251585
C 2.200866 6.336386 1.723146
C 0.963161 6.676351 1.183539
C -0.025064 5.694659 1.216104
C 0.219541 4.416227 1.731247
C 3.850595 4.894476 2.854629
C -0.944923 3.461140 1.757606
C 0.706402 8.039246 0.595919
C 3.282376 1.956325 2.686051
C 3.893047 1.841834 1.405573
C 5.200162 1.362507 1.250743
C 5.977537 0.970831 2.339481
C 5.402665 1.106171 3.600569
C 4.100659 1.589388 3.790178
C 3.183997 2.280429 0.148019
C 7.373615 0.432427 2.163047
C 3.659975 1.734611 5.229263
C 0.651311 4.434205 5.103239
C -1.509183 0.666550 7.554512
C 1.118222 -0.401368 3.437843
C -3.814140 -1.694437 -2.294283
C -3.284303 -2.913030 -3.030150
C -1.607429 -2.047146 -4.591292
C -0.331622 -1.230796 -4.485829
C -0.707246 2.514333 -2.350829
C -0.560802 4.025284 -2.590303
C -0.678973 -4.224052 -1.766033
C -1.094606 -3.988549 -3.206252
C -5.483072 -0.427171 0.316768
C -5.197706 0.986448 -0.199919
C -2.750423 -1.189067 1.887310
C -3.098898 -1.874783 3.218906
C -2.592858 0.321154 2.115100
C -4.701480 -3.362393 0.759023
C -5.926182 -3.752171 -0.080127
C 2.691328 -2.095117 -0.009964
C 3.263982 -1.270553 -1.170117
C 2.608340 -4.688353 -1.931826
C 2.843275 -4.015152 -3.289718
C 1.404048 -4.771170 0.929958
C 0.562093 -4.078783 2.006736
C 3.927515 -5.271022 -1.398537
C -3.678353 -4.502710 0.752840
C -6.287772 -0.381553 1.626395
C -1.598141 1.721090 -5.364252
C -1.309040 2.996762 -6.172825
C 1.393174 1.887971 -4.576983
C 1.852219 1.070982 -5.792879
C -3.081989 1.681982 -4.975662

C 2.480739 1.900844 -3.499172
C -2.056709 2.215751 -1.690618
C 0.868686 -6.181224 0.649348
C 3.796477 -2.409201 1.011221
B 1.718510 2.486574 2.770446
H -2.014413 3.088951 -7.008880
H -3.718573 1.629181 -5.868291
H -1.418171 3.898896 -5.561209
H -3.374790 2.585826 -4.431481
H -0.301383 3.006837 -6.597344
H -3.332821 0.825441 -4.340894
H -1.418333 0.871986 -6.041798
H -1.351443 4.406837 -3.246726
H -2.890457 2.599894 -2.285532
H 1.108157 1.041070 -6.597089
H 0.402095 4.283811 -3.041940
H -0.623692 4.570349 -1.641755
H 1.257816 2.926926 -4.918228
H -0.153138 -0.730755 -5.445184
H -2.440529 -1.365574 -4.784490
H -6.134802 1.542095 -0.334272
H -2.118504 2.669966 -0.697599
H -1.573315 -2.778674 -5.414045
H 2.768994 1.498635 -6.218061
H -2.271731 1.140743 -1.556608
H -4.684093 0.974223 -1.165702
H 2.088021 0.037424 -5.515705
H -3.781830 -0.813603 -2.952187
H -4.579381 1.560644 0.495613
H -3.834278 -3.113920 -3.962325
H 0.532972 -1.891819 -4.336042
H 0.080920 2.222387 -1.641453
H -4.876623 -1.848275 -2.065108
H 2.240080 2.566594 -2.666445
H -7.210397 0.196540 1.490186
H -6.126880 -0.914680 -0.431515
H 3.437062 2.237939 -3.918870
H -0.194646 -3.902723 -3.819931
H 2.645985 0.902728 -3.082222
H -1.689389 -4.824530 -3.606127
H -3.399945 -3.785689 -2.381830
H -5.724451 0.101768 2.431357
H 2.520284 -1.056553 -1.948150
H -6.578888 -1.376964 1.978516
H -6.729688 -3.011800 -0.025894
H 3.644736 -0.313222 -0.801995
H 1.935521 -3.555412 -3.693108
H 3.599349 -3.227597 -3.224772
H -5.673203 -3.889998 -1.138314
H 4.100290 -1.787826 -1.653286

H 3.199874 -4.744359 -4.028720
H -1.563775 -4.389286 -1.139279
H -0.110909 -5.161279 -1.727486
H -1.763759 -1.580827 1.578554
H -6.343189 -4.706061 0.267961
H 1.952023 -1.466196 0.505095
H -4.087850 -1.568376 3.579240
H -5.051906 -3.253428 1.797924
H -3.398830 -4.771912 -0.272006
H -2.375121 -1.582005 3.987459
H 1.937085 -5.542877 -2.107475
H 4.293851 -1.480969 1.313845
H 4.674377 -4.487492 -1.233023
H -3.093349 -2.966977 3.155089
H 4.358324 -5.976454 -2.120765
H -2.757666 -4.257012 1.291359
H 4.563493 -3.073453 0.597216
H -4.094106 -5.408559 1.211891
H -0.466875 -3.923671 1.661728
H 3.405341 -2.876329 1.919533
H 0.962218 -3.100612 2.287360
H -0.181200 -6.162746 0.336359
H 3.800448 -5.809753 -0.454322
H 1.438648 -6.708661 -0.123145
H 2.421838 -4.889380 1.333994
H 0.509021 -4.686073 2.919254
H 0.912170 -6.794978 1.558208
H -1.024520 5.934305 0.850583
H 2.985809 7.092126 1.752794
H 3.814868 4.381048 3.818535
H 4.515688 4.301934 2.218831
H 4.319898 5.871753 3.011563
H -0.349834 8.318026 0.674189
H 1.295785 8.811257 1.101683
H 0.973157 8.077945 -0.468797
H 5.994467 0.846408 4.478461
H 5.630274 1.316256 0.249541
H 4.524736 1.671292 5.898608
H 3.161348 2.689417 5.412006
H 2.948215 0.959022 5.528655
H 8.029240 0.742214 2.983827
H 7.385822 -0.665496 2.140768
H 7.821545 0.779945 1.226187
H -0.736961 3.207045 6.933466
H -0.318113 -0.825343 5.593934
H 0.411539 4.871314 6.078608
H 1.698417 4.655230 4.883989
H 0.063342 4.962526 4.346388
H 2.156183 -0.219215 3.149885
H 1.058972 -1.379168 3.928532

H 0.537513 -0.462042 2.508080
H -2.583379 0.738258 7.337529
H -1.298467 -0.378819 7.803582
H -1.323621 1.268852 8.450229
H 3.860177 2.234884 -0.712370
H 2.313574 1.650081 -0.070189
H 2.804116 3.301954 0.236003
H -1.791137 0.524081 2.833014
H -2.354178 0.866442 1.198280
H -3.510653 0.753663 2.527498
H -1.117742 3.066705 2.763344
H -1.864294 3.951898 1.417748
H -0.756810 2.588500 1.122300
H 1.151369 1.789227 1.863955
H 0.414756 0.629834 0.626156

Supplementary Table 20. Complex 2D1 in U(VI) profile optimised geometry and energy

Energy: : -3137.56497 a.u
U 0.812798 -1.981103 -1.168571
Si 3.115305 -4.808258 -0.546504
Si -2.579639 -2.707475 0.477830
Si 1.272454 0.480802 -4.023783
N 1.556987 -4.071733 -1.085669
N 1.218831 -1.136230 -3.216575
N -0.321761 -3.536154 -3.159935
N -1.384281 -2.317301 -0.817963
N 1.487914 -0.536853 0.023611
C 5.237135 -3.735755 1.122133
C 2.358497 -7.448685 0.594738
C -0.493059 1.613420 -2.014775
C 4.094598 1.036598 -3.687927
C -1.433583 0.536046 -5.207834
C 3.518298 -0.038035 -5.876481
C 3.011550 0.882019 -4.756070
C 0.369250 1.619052 -6.559717
C 0.062121 0.501776 -5.547963
C -4.876900 -1.425964 1.705041
C -2.878989 -5.624917 0.079144
C 5.213415 -6.534513 -1.468511
C 1.899575 -5.416926 1.997258
C 2.816944 -6.022384 0.931128
C 4.099556 -5.267557 -3.327807
C 3.896196 -5.897842 -1.945192
C 5.004741 -2.728850 -1.172296
C 4.250319 -3.363206 0.005026
C -4.737069 -4.237020 -0.901316
C -3.648718 -4.301999 0.178984
C -1.177847 -1.676402 2.756503
C -2.379540 -3.838714 3.158316
C -1.630187 -2.992364 2.116691

C -3.291407 0.133460 0.512747
C -3.880760 -1.279606 0.544110
C 0.214455 -4.894201 -2.977937
C 0.597660 -5.107760 -1.523888
C 0.957111 3.248053 -3.250607
C 0.857094 1.812040 -2.714530
C 1.409694 -2.207718 -4.219562
C 0.100012 -2.942532 -4.438732
C -1.776744 -3.446338 -2.949294
C -2.057384 -2.197841 -2.126903
C 2.473668 -1.856390 3.185324
C 0.206935 -0.562903 7.448656
C 1.895488 3.077785 4.498799
C 4.856740 0.592657 4.835257
C 8.714639 -0.629275 1.952766
C 4.476310 0.703781 -0.306530
C 5.305106 0.353255 3.409619
C 6.648198 -0.027764 3.283401
C 7.275269 -0.200964 2.053692
C 6.505057 0.052514 0.921438
C 5.157771 0.420566 1.004109
C 4.490326 0.564922 2.253364
C 1.190990 6.231287 -0.175940
C -0.004355 1.791847 1.753071
C 4.852804 3.507202 1.796158
C 1.119199 2.711772 1.341365
C 0.722357 3.920058 0.749948
C 1.626374 4.933847 0.451840
C 2.957505 4.708226 0.800055
C 3.396766 3.516801 1.385487
C 2.486609 2.442001 1.640653
C 1.972497 -0.737290 4.059022
C 1.371321 -1.104735 5.267816
C 0.889444 -0.158734 6.169381
C 1.070231 1.180685 5.832304
C 1.680713 1.585846 4.639343
C 2.134080 0.623574 3.689287
B 2.916763 1.018085 2.318591
H 1.396580 0.455026 -0.232575
H 2.284261 0.065987 1.298631
H 0.243872 0.738536 1.632827
H -0.911129 2.006675 1.179684
H -0.246678 1.929773 2.812755
H -2.034070 -1.083390 3.096066
H -0.597377 -1.055162 2.070370
H -0.548829 -1.856117 3.633459
H 3.925911 1.647817 -0.284799
H 3.750411 -0.073875 -0.560400
H 5.213259 0.757695 -1.111554
H 0.361771 0.179640 8.237904

H 0.575851 -1.526890 7.813815
H -0.877190 -0.663354 7.308138
H 1.883887 -1.935155 2.264849
H 2.411027 -2.810009 3.717721
H 3.511793 -1.698061 2.882387
H 1.223006 3.540215 3.770215
H 2.910605 3.319928 4.173890
H 1.728834 3.570324 5.462124
H 1.288673 -2.163177 5.514375
H 0.748107 1.946169 6.537444
H 9.192425 -0.221532 1.056023
H 8.803924 -1.721951 1.898165
H 9.292016 -0.301761 2.823048
H 4.187994 -0.186961 5.209504
H 4.321111 1.537459 4.946357
H 5.729688 0.627554 5.494813
H 6.971361 -0.012448 -0.061411
H 7.232400 -0.168195 4.191937
H 1.612480 6.354384 -1.181228
H 1.524015 7.092041 0.415160
H 0.101835 6.287739 -0.263753
H 5.236686 4.532112 1.820675
H 5.485352 2.934367 1.111731
H 5.000180 3.068150 2.784900
H 3.684630 5.502479 0.634144
H -0.337587 4.082931 0.555705
H 2.384062 -8.077814 1.494199
H 1.868390 -6.044582 2.897433
H 3.821924 -6.109597 1.372313
H 2.985645 -7.936952 -0.157274
H 5.115442 -7.050224 -0.508200
H 1.324857 -7.470935 0.231642
H 2.219470 -4.417942 2.302910
H 4.729027 -4.079478 2.027690
H 0.871422 -5.330906 1.626908
H -3.548497 -6.480904 0.236279
H 5.928511 -4.526741 0.807749
H -2.070273 -5.704842 0.812899
H 5.576765 -7.270295 -2.197862
H -2.635724 -4.840515 2.801228
H 6.001929 -5.782469 -1.355409
H 5.838470 -2.862289 1.398839
H 3.173423 -6.718619 -2.073985
H -1.761175 -3.961325 4.056275
H -2.432020 -5.760669 -0.912131
H -4.178833 -4.327508 1.144738
H -3.309106 -3.354930 3.478368
H 3.582833 -2.592036 0.422784
H -5.389769 -5.118528 -0.846531
H -0.726942 -3.554509 1.830086

H 1.005733 -6.122978 -1.428539
H -0.302013 -5.086473 -0.897918
H 4.404594 -6.029291 -4.057636
H 5.731688 -3.427399 -1.599352
H -4.318639 -4.221650 -1.914015
H 4.881383 -4.502999 -3.318616
H 3.191053 -4.795625 -3.712157
H 5.559329 -1.848487 -0.834392
H -5.377871 -3.356319 -0.797370
H -5.376657 -2.400550 1.715178
H 4.340184 -2.410791 -1.984916
H -4.385711 -1.296697 2.675682
H -2.104434 -4.317994 -2.379138
H -0.504436 -5.652597 -3.326839
H 4.266305 0.091608 -3.163133
H 1.113948 -4.993644 -3.590525
H 5.049668 1.339651 -4.137195
H -4.460877 -1.401493 -0.383843
H -5.660825 -0.660205 1.640361
H 3.836893 1.786576 -2.935363
H -3.140542 -2.035743 -2.036167
H 1.646218 1.706578 -1.954087
H 2.178885 -2.920062 -3.888288
H -2.315681 -3.453331 -3.909283
H -2.743580 0.363978 1.430399
H -1.686399 -1.320677 -2.682016
H 3.824351 -1.013673 -5.482553
H -2.597421 0.267497 -0.321587
H -0.633389 0.601117 -1.612580
H 4.404598 0.396264 -6.357213
H 0.173085 -3.710403 -5.225397
H -0.589284 2.303115 -1.170008
H -4.086749 0.883722 0.411321
H -0.654311 -2.217619 -4.755773
H 1.771248 -1.828876 -5.183339
H 2.846236 1.874059 -5.204994
H 0.836579 3.964955 -2.430748
H 1.922559 3.453191 -3.723728
H 2.777876 -0.213453 -6.664935
H -1.331881 1.800562 -2.691568
H 0.173400 3.458772 -3.987134
H 0.254684 -0.449908 -6.067506
H -1.721406 -0.206490 -4.457282
H 1.388120 1.571063 -6.952924
H -1.729693 1.517278 -4.822620
H 0.233417 2.612837 -6.120031
H -2.042053 0.351394 -6.103138
H -0.312587 1.554710 -7.418122

Supplementary Table 21. Complex 2D2 in U(VI) profile optimised geometry and energy

Energy: -3137.988399 a.u

U 0.933565 -2.140700 -1.116458
Si 3.410360 -4.602939 -0.607337
Si 1.158586 0.775506 -3.491889
Si -2.198257 -3.012839 0.688177
N -0.109087 -3.533746 -3.193866
N 1.767323 -4.130994 -1.272791
N 1.120342 -1.002572 -2.964979
N -1.171859 -2.572862 -0.776418
N 1.250753 -0.726815 0.303913
C 2.923836 0.761344 -5.903423
C -0.192561 2.573550 -5.240368
C 2.239458 -5.446189 1.881160
C 4.647272 -4.518801 -3.277841
C 4.289766 -1.871432 -0.925829
C 5.810359 -5.905130 -1.520148
C 4.454838 -5.379312 -2.023572
C 5.325713 -3.136910 0.982166
C 4.061223 -2.965088 0.122420
C -4.206146 -4.925763 -0.382412
C -1.420199 -4.026437 3.306477
C -3.329142 -0.297281 0.603087
C -1.041424 -1.589109 2.898046
C -1.067958 -2.974064 2.242034
C -4.588835 -2.028020 1.959914
C -3.681112 -1.782288 0.742610
C 1.729138 3.255076 -2.098595
C -1.691406 0.766932 -4.352698
C -0.255239 1.110434 -4.763623
C -0.348865 2.039314 -1.375809
C 1.072450 1.875291 -1.930466
C 1.313565 -1.837144 -4.178201
C 0.086842 -2.697452 -4.395136
C 1.069300 -5.250310 -1.928329
C 0.625230 -4.807192 -3.308429
C -1.541026 -3.735124 -2.891676
C -2.005586 -2.598275 -2.002502
C 5.299052 0.256955 5.105207
C 8.825323 -0.014741 1.606503
C 4.319177 1.600427 0.214094
C 0.127647 1.823889 2.552877
C 1.001068 6.579119 1.332956
C 4.848913 3.843259 2.911853
C 2.780511 -1.818625 3.433173
C 0.532614 -1.242541 7.854002
C 2.141581 2.848526 5.520108
C 1.176226 2.880623 2.307126
C 0.693374 4.127570 1.891294
C 1.525129 5.237001 1.765207

C 2.869091 5.061704 2.103855
C 3.392143 3.830147 2.502867
C 2.556362 2.672645 2.575039
C 2.280406 -0.849192 4.473705
C 1.681016 -1.414512 5.603950
C 1.194722 -0.633295 6.649137
C 1.366011 0.747125 6.537690
C 1.959746 1.349579 5.425527
C 2.420851 0.557188 4.328845
C 2.868047 -7.330703 0.327579
C 3.207969 -5.906068 0.787443
C 4.041984 0.574022 -3.655112
C 2.824192 1.113210 -4.411406
C -2.019755 -5.938531 0.331072
C -2.990918 -4.771402 0.543397
C 5.600824 0.401563 3.630854
C 6.922576 0.121388 3.263863
C 7.390134 0.263621 1.959359
C 6.479090 0.724917 1.008994
C 5.153316 1.026800 1.333157
C 4.654922 0.850786 2.658979
B 3.109506 1.211574 3.013616
H 2.487701 0.357424 1.937133
H 0.447716 -0.093625 0.421198
H 0.531320 0.813822 2.529872
H -0.698417 1.907635 1.837006
H -0.307724 1.946900 3.552004
H -2.010878 -1.348070 3.344300
H -0.802946 -0.793889 2.191031
H -0.297321 -1.546500 3.700029
H 4.056527 2.644784 0.399317
H 3.372550 1.069768 0.079941
H 4.875056 1.562020 -0.727224
H -0.549438 -1.062462 7.844464
H 0.918769 -0.810370 8.782932
H 0.687428 -2.324419 7.894531
H 2.031759 -1.968762 2.643771
H 2.978305 -2.794272 3.888039
H 3.698332 -1.469622 2.957430
H 1.430356 3.401249 4.899490
H 3.138957 3.165285 5.207140
H 2.001924 3.175029 6.554741
H 1.611070 -2.499286 5.676724
H 1.042723 1.382237 7.360775
H 8.941904 -0.251490 0.544720
H 9.228546 -0.847972 2.190154
H 9.455757 0.858508 1.815288
H 4.733254 -0.651496 5.331481
H 4.709488 1.090034 5.492879
H 6.233039 0.214512 5.672866

H 6.818642 0.878624 -0.014474
H 7.616484 -0.206419 4.036079
H -0.064674 6.536759 1.092010
H 1.532668 6.950220 0.449355
H 1.135400 7.328312 2.121596
H 5.151972 4.862357 3.169643
H 5.515174 3.495562 2.115617
H 5.045990 3.206530 3.776412
H 3.534073 5.923536 2.072953
H -0.372514 4.240514 1.695205
H 2.933992 -8.027231 1.171881
H 2.222402 -6.159963 2.713548
H 4.216823 -5.938064 1.228245
H 3.543496 -7.703104 -0.448906
H 5.714782 -6.569993 -0.656294
H 1.844221 -7.400573 -0.056053
H 2.505845 -4.470426 2.297394
H 5.172571 -3.842623 1.803738
H 1.215623 -5.376694 1.496021
H -2.483209 -6.889335 0.620543
H 6.169424 -3.497328 0.384776
H -1.092469 -5.840407 0.903959
H 6.315845 -6.472261 -2.310852
H -1.344321 -5.055183 2.945237
H 6.480496 -5.087273 -1.236464
H 5.625499 -2.179462 1.420758
H 3.868621 -6.261613 -2.324454
H -0.743795 -3.929132 4.163423
H -1.747183 -6.039070 -0.725349
H -3.371530 -4.865502 1.573138
H -2.436972 -3.882432 3.689146
H 3.274510 -2.614810 0.813648
H -4.685445 -5.899444 -0.222159
H -0.046997 -3.198223 1.887735
H 1.709343 -6.134281 -2.036260
H 0.211071 -5.566411 -1.325628
H 5.058293 -5.118624 -4.098639
H 5.106726 -2.133470 -1.604023
H -3.926562 -4.889385 -1.441685
H 5.350980 -3.698992 -3.103555
H 3.709958 -4.076889 -3.631586
H 4.547267 -0.924690 -0.445723
H -4.971146 -4.162366 -0.214340
H -4.924448 -3.067539 2.033310
H 3.431671 -1.662506 -1.595907
H -4.091257 -1.775043 2.901433
H -1.652640 -4.673885 -2.344590
H 0.013150 -5.570165 -3.811395
H 4.095964 -0.518610 -3.723356
H 1.511690 -4.638253 -3.925623

H 4.973578 0.968536 -4.078843
H -4.276739 -2.047782 -0.144055
H -5.488105 -1.403369 1.897315
H 4.024277 0.838841 -2.594389
H -3.070142 -2.724592 -1.774316
H 1.661690 1.331918 -1.180766
H 2.209179 -2.467406 -4.079961
H -2.129290 -3.814125 -3.817712
H -2.811109 0.085476 1.486516
H -1.934674 -1.643013 -2.537605
H 3.039765 -0.316049 -6.068017
H -2.692217 -0.106704 -0.267106
H -0.879019 1.086338 -1.251943
H 3.811586 1.233726 -6.341287
H 0.164975 -3.321111 -5.297380
H -0.318110 2.538367 -0.401071
H -4.238542 0.303128 0.478775
H -0.781303 -2.046503 -4.518367
H 1.487706 -1.230326 -5.073060
H 2.863805 2.211316 -4.348494
H 1.653171 3.816111 -1.160431
H 2.792325 3.184500 -2.345661
H 2.061466 1.098159 -6.486221
H -0.964515 2.661519 -2.033106
H 1.243782 3.853390 -2.877241
H -0.005574 0.480422 -5.630884
H -1.800504 -0.271670 -4.027447
H 0.801496 2.870955 -5.587732
H -2.047655 1.401624 -3.536909
H -0.483912 3.264916 -4.442753
H -2.376442 0.914776 -5.196642
H -0.888287 2.734315 -6.072728
H 1.997269 -0.226478 1.162119

Supplementary Table 22. Complex 4 in U(VI) profile optimised geometry and energy
Energy: -2063.775991 a.u
U -0.936267 -1.292592 -1.754449
Si 1.845440 -3.706676 -1.368435
Si -0.767803 1.650151 -3.977750
Si -4.008591 -2.036366 0.274602
N -1.969914 -2.725838 -3.768758
N 0.250490 -3.225999 -2.004280
N -0.795161 -0.111086 -3.706589
N -3.106101 -1.808752 -1.241517
N -0.067592 -0.179681 -0.039052
C 1.138040 1.757930 -6.198429
C -2.100539 3.593267 -5.704213
C 0.870321 -4.330878 1.255466
C 2.969653 -3.809349 -4.062866
C 3.018316 -1.066087 -1.724373

C 4.221226 -5.118250 -2.308031
C 2.845495 -4.607329 -2.759207
C 3.957359 -2.417429 0.179075
C 2.710989 -2.137087 -0.671589
C -5.594266 -4.452035 -0.310389
C -3.196755 -2.169352 3.077942
C -5.583926 0.374111 -0.293081
C -2.869257 0.043944 1.951401
C -2.883944 -1.477734 1.741784
C -6.475351 -1.148881 1.517558
C -5.685448 -1.073199 0.202933
C -0.583871 3.998324 -2.267356
C -3.593001 1.799041 -4.768710
C -2.168471 2.134850 -5.227695
C -2.269340 2.275437 -1.558978
C -0.923583 2.501346 -2.259108
C -0.798040 -0.911086 -4.942426
C -1.990104 -1.859946 -4.962517
C -0.481314 -4.333662 -2.640915
C -1.094045 -3.895733 -3.963362
C -3.328060 -3.110654 -3.338072
C -3.929889 -2.030215 -2.446035
C 1.155898 -6.329989 -0.252431
C 1.690143 -4.938399 0.111380
C 2.108559 1.986509 -3.893883
C 0.884030 2.264279 -4.772119
C -3.272908 -4.820669 0.578411
C -4.487609 -3.886776 0.590128
H -0.479939 0.060505 0.857896
H -3.830290 0.395750 2.341755
H -2.670029 0.599933 1.030707
H -2.107647 0.331550 2.689971
H 1.191410 -7.001750 0.615892
H 0.793946 -5.023337 2.104595
H 2.721371 -5.072112 0.472483
H 1.733562 -6.805440 -1.052767
H 4.170270 -5.718736 -1.393620
H 0.110337 -6.289851 -0.577674
H 1.305639 -3.398571 1.630054
H 3.753928 -3.103749 1.006897
H -0.149474 -4.103335 0.924355
H -3.550932 -5.842604 0.869263
H 4.770989 -2.849011 -0.413592
H -2.481708 -4.491556 1.259643
H 4.679343 -5.745775 -3.084354
H -3.100738 -3.257426 3.026667
H 4.915233 -4.291476 -2.120703
H 4.341884 -1.486853 0.617791
H 2.241280 -5.499625 -2.985696
H -2.511040 -1.819958 3.861217

H -2.829977 -4.877538 -0.422255
H -4.884683 -3.876875 1.617002
H -4.213194 -1.944506 3.420538
H 1.947541 -1.720894 0.002186
H -5.872171 -5.467059 0.004295
H -1.866442 -1.786696 1.451990
H 0.154848 -5.204838 -2.855711
H -1.277589 -4.717811 -1.983145
H 3.366374 -4.433891 -4.874459
H 3.846714 -1.365797 -2.375979
H -5.269935 -4.526674 -1.354931
H 3.653423 -2.961545 -3.948846
H 2.006937 -3.404551 -4.390367
H 3.313732 -0.117850 -1.254665
H -6.506521 -3.847019 -0.290182
H -6.629568 -2.179312 1.855637
H 2.163285 -0.855952 -2.378056
H -5.966446 -0.611464 2.325684
H -3.251658 -4.032493 -2.753506
H -1.646669 -4.718393 -4.448296
H 2.295770 0.910812 -3.814269
H -0.284608 -3.601650 -4.637210
H 3.013072 2.441580 -4.319474
H -6.273291 -1.622401 -0.548574
H -7.467632 -0.691234 1.406915
H 1.996030 2.375943 -2.876270
H -4.959727 -2.334353 -2.209590
H -0.161649 1.996659 -1.645709
H 0.133775 -1.490233 -5.041470
H -3.966581 -3.321473 -4.212754
H -5.067656 1.016459 0.427116
H -4.039008 -1.101521 -3.029920
H 1.315407 0.676666 -6.213734
H -5.040212 0.445567 -1.239808
H -2.590803 1.225440 -1.559530
H 2.030635 2.231659 -6.628913
H -2.017965 -2.468768 -5.882388
H -2.223621 2.597841 -0.511169
H -6.582254 0.804500 -0.449539
H -2.905741 -1.262298 -4.935903
H -0.851400 -0.300226 -5.855373
H 0.759819 3.356889 -4.832762
H -0.596794 4.404576 -1.247263
H 0.406616 4.202058 -2.685318
H 0.302978 1.966433 -6.875984
H -3.071022 2.847256 -2.038091
H -1.310938 4.576406 -2.848484
H -1.960280 1.506200 -6.107962
H -3.667213 0.795829 -4.337504
H -1.121358 3.857807 -6.115077

H -3.944170 2.503407 -4.007552
H -2.313509 4.291833 -4.887469
H -4.301987 1.856126 -5.605377
H -2.845510 3.784254 -6.488359
H 0.856332 0.247575 -0.067234

Supplementary Table 23. Computed data for the conversion of 1 to 4 (Figure 9)

**U(V)**	Spin density	Atomic Charge	UN length (Å)	Wiberg	Spin	Multiplicity	Contamination	TS imaginary Frequency (cm^{-1})
**A**	U=1.182<br>N=-0.128	U=0.994<br>N=-0.479	1.77372	U-N=2.7151	1/2	2	0.750	
**B**	U=1.243<br>N=-0.149	U=1.097<br>N=-0.558	1.84462	U-N=2.5485	1/2	2	0.750	-1090.337
**C**	U=1.251<br>N=-0.088	U=1.279<br>N=-0.750	1.92747	U-N=1.9751	1/2	2	0.750	
**D**	U=2.340<br>N=-0.420	U=1.090<br>N=-0.686	2.10197	U-N=1.2573	3/2	4	3.750	-1098.673
**6**	U=3.133<br>N=-0.039	U=1.141<br>N=-0.930	2.314	U-N=0.7706	3/2	4	3.750	
**4**	U=2.169<br>N=-0.044	U=1.579<br>N=-0.930	2.2240	U-N=0.8611	1	3	2.000	

Supplementary Table 24. Computed data for the conversion of 1 to 4 (Figure 10)

**U(V)**	**2A**	**2B**	**2C**	**2D1**	**2D2**	**6**	**4**
Spin density	U=1.203<br>N=-0.128	U=1.209<br>N=-0.119	U=1.251<br>N=-0.139	U=2.037<br>N=-0.423	U=2.011<br>N=-0.420	U=3.133<br>N=-0.038	U=2.170<br>N=-0.048
Atomic Charge	U=1.057<br>N=-0.512	U=1.289<br>N=-0.602	U=1.604<br>N=-0.736	U=1.469<br>N=-0.778	U=1.289<br>N=-0.604	U=1.141<br>N=-0.930	U=1.564<br>N=-0.922
UN length (Å)	1.778	1.819	1.927	2.005	1.821	2.314	2.221
Wiberg	U-N=2.720	U-N=2.501	U-N=1.977	U-N=1.391	U-N=1.219	U-N=0.771	U-N=0.861
Spin	1/2	1/2	1/2	1/2	1/2	3/2	1
Multiplicity	2	2	2	2	2	4	3
Contamination	0.750	0.750	0.751	0.751	0.750	3.750	2.000
TS imaginary Frequency (cm^{-1})		-918.227		-1214.225	-888.023		

Supplementary Table 25. Computed data for the hypothetical conversion of 2 to 4

**U(VI)**	**2A**	**2B**	**2C**	**2D1**	**2D2**	**4**
Atomic charge	U=1.389<br>N=-0.412	U=1.641<br>N=-0.589	U=1.746<br>N=-0.744	U=1.625<br>N=-0.690	U=1.782<br>N=-0.812	U=1.784<br>N=-0.898
UN length (Å)	1.736	1.817	1.866	1.991	2.029	2.146
Wiberg	U-N=2.954	U-N=2.477	U-N=2.297	U-N=1.673	U-N=1.543	U-N=1.113
Spin	0	0	0	0	0	1/2
Multiplicity	1	1	1	1	1	2
TS imaginary Frequency (cm^{-1})		-1107.051		-1338.587	-1418.904	

Supplementary Methods

General experimental details

All manipulations were carried out using Schlenk techniques, or an MBraun UniLab glovebox, under an atmosphere of dry nitrogen or argon. Solvents were dried by passage through activated alumina towers and degassed before use. All solvents were stored over potassium mirrors except for ethers, which were stored over activated 4 Å sieves. Deuterated solvent was distilled from potassium, degassed by three freeze-pump-thaw cycles and stored under nitrogen. The compounds $[U^{V}(Tren^{TIPS})(N)][K(B15C5)_2]$ (**1**) and $[U^{VI}(Tren^{TIPS})(N)]$ (**2**) $[Tren^{TIPS} = N(CH_2CH_2NSiPr^i{}_3)_3{}^{3-}$; B15C5 = benzo-15-crown-5 ether] were prepared as described previously.[1,2] Boranes were dried under dynamic vacuum for 12 hours prior to use. H_2 was passed through a column of activated 4 Å sieves prior to use. D_2 (99.9% atom D) and all other reagents were used as supplied.

^{1}H and ^{11}B NMR spectra were recorded on a Bruker 400 spectrometer operating at 400.2 and 128.4 MHz respectively; chemical shifts are quoted in ppm and are relative to $SiMe_4$ (^{1}H) and $BF_3{\bullet}OEt_2$ (^{11}B). FTIR spectra were recorded on a Bruker Alpha spectrometer with Platinum-ATR module. UV/Vis/NIR spectra were recorded on a Perkin Elmer Lambda 750 spectrometer. Data was collected in 1mm path length cuvette loaded in an MBraun UniLab glovebox and was run versus reference solvent. Variable temperature EPR spectra were measured at X-band (ca. 9.3-9.8 GHz) on a Bruker EMX 300 spectrometer equipped with a liquid helium cryostat or on a Bruker EMX Micro spectrometer. Variable-temperature magnetic moment data were recorded in an applied dc field of 0.1 T on a Quantum Design MPMS XL7 superconducting quantum interference device magnetometer using doubly recrystallised powdered samples. Care was taken to ensure complete thermalisation of the sample before each data point was measured and samples were immobilised in an eicosane

matrix to prevent sample reorientation during measurements. Crystals were examined using either a) an Agilent Supernova diffractometer, equipped with an Eos CCD area detector and a Microfocus source with Mo Kα radiation (λ = 0.71073 Å) or b) a Rigaku FR-X diffractometer, equipped with a HyPix 6000HE photon counting pixel array detector with mirror-monochromated Mo Kα (λ = 0.71073 Å) or Cu Kα (λ = 1.5418 Å) radiation. The structures were solved by direct or iterative methods using either SHELXS[3] or SHELXT.[4] CrysAlisPro[5] was used for control and integration, and SHELXL[6] and Olex2[7] were employed for structure refinement. ORTEP[8] and POV-Ray[9] were employed for molecular graphics. Elemental microanalyses were carried out by Mr Martin Jennings at the Micro Analytical Laboratory, School of Chemistry, The University of Manchester.

Reaction between [U(Tren^{TIPS})(N)][K(B15C5)$_2$] (1) and BCF

A suspension of BCF (0.005 g, 0.01 mmol) in C_6D_6 (0.2 ml) was added to a solution of **1** in C_6D_6 (0.3 ml). The resulting mixture was agitated to afford a dark brown solution, which was then analysed by NMR spectroscopy. ^{1}H NMR (C_6D_6, 298 K): δ 35.85 (m, br, 4H), 32.38 (s, 1H), 29.42 (s, 1H), 24.77 (s, 1H), 7.85 (s, 1H) 6.83 (s, 4H), 5.15 (s, 3H), 4.40 (s, 12H), 4.03 (s, 15H), 2.45 (s, 2H), 2.27 (s, 2H), 1.65 (s, 12H), 1.52 (s, 2H), 1.17 (s, 4H), −5.23 (s, 2H), −5.57 (s, 1H), −6.12 (s, 4H), −6.59 (s, 24H), −8.96 (br, s, 4H), −14.28 (d, J= 76 Hz, 1H), −22.74 (s, 2H). ^{19}F{^{1}H} NMR (C_6D_6, 298 K): δ −132.25, −133.20, −133.99, −150.98, −153.87, −159.52, −159.86, −160.91, −161.43, −161.91, −162.91, −163.94, −165.59, −166.18, −166.73.

Reaction between [U(Tren^{TIPS})(N)] (2) and BCF

A solution of **2** (0.010 g, 0.012 mmol) in C_6D_6 (0.4 ml) was added to a suspension of BCF (0.006 g, 0.012 mmol) in C_6D_6. The resulting red solution was agitated briefly and then

analysed by NMR spectroscopy. ^{1}H NMR (C_6D_6, 298 K): (intensities relative to the weakest being 1H) δ 65.91 (s, 1H), 53.61 (s, 1H), 45.35 (s, 1H), 31.88 (s, 2H), 28.73 (s, 3H), 23.06 (m, 1H), 21.45 (m, 1H), 18.96 (m, 1H), 17.34 (m, 2H), 15.19 (m, 2H) , 14.05 (s, 12H), 11.71 (s, 2H), 10.58 (m, 2H), 9.01 (s, 2H), 5.16 (m, 3H), 2.89 (m, 9H), 1.69 (m, 9H), 1.13 (m, 12H), 0.28 (s, 4H), -0.68 (s, 4H), −1.48 (s, 4H), −2.70 (s, 1H), −3.39 (s, 2H), −4.40 (s, 4H), −5.69 (s, 2H), −12.22 (m, 24H), −15.41 (s, 1H). ^{19}F{^{1}H} NMR (C_6D_6, 298 K): δ −124.92, −129.87, −132.51, −134.55, −139.10, −150.90, −157.33, −160.05, −160.89, −162.32, −163.75, −166.51.

Attempted reaction between [U(Tren^{TIPS})(N)] (2) and H_2

A red-brown solution of **2** (0.04 g, 0.05 mmol) in C_6D_6 (0.5 ml) was degassed and exposed to H_2 (1 atm.). The red-brown solution was analysed over 10 days by ^{1}H NMR spectroscopy, with no reaction observed.

Attempted reaction between [U(Tren^{TIPS})(N)] (2), BPh_3, and H_2

A red-brown solution of **2** (0.03 g, 0.04 mmol) and BPh_3 (0.008 g, 0.04 mmol) in C_6D_6 (0.5 ml) was degassed and exposed to H_2 (1 atm.). The red-brown solution was analysed for 8 days by ^{1}H NMR spectroscopy, with no reaction observed.

Attempted reaction between [U(Tren^{TIPS})(N)], $BMes_3$, and H_2

A red-brown solution of **2** (0.03 g, 0.04 mmol) and $BMes_3$ (0.013 g, 0.04 mmol) in C_6D_6 (0.5 ml) was degassed and exposed to H_2 (1 atm.). The red-brown solution was analysed for 7 days by ^{1}H NMR spectroscopy, with no reaction observed.

NMR-scale reactions of 1, $BMes_3$, and H_2/D_2 in H_6-/D_6-benzene or H_8-/D_8-toluene combinations

A J Youngs-valve NMR tube was charged with **1** (72 mg, 50 μmol) and $BMes_3$ (18.4mg, 50 μmol). Solvent (0.8 ml, H_6-/D_6-benzene or H_8-/D_8-toluene) was added and the resulting mixture degassed and exposed to an atmosphere of H_2 or D_2. The resulting brown mixture was left to stand for 1 week and then analysed by ^1H and ^2H NMR spectroscopies.

	NH_2	NHD	ND_2
1 + $BMes_3$ + H_2 + D_6-benzene	✓	×	×
1 + $BMes_3$ + H_2 + D_8-toluene	✓	×	×
1 + $BMes_3$ + D_2 + D_6-benzene	✓	✓	✓
1 + $BMes_3$ + D_2 + D_8-toluene	✓	✓	✓
1 + $BMes_3$ + H_2 + H_6-benzene	✓	×	×
1 + $BMes_3$ + H_2 + H_8-toluene	✓	×	×
1 + $BMes_3$ + D_2 + H_6-benzene	✓	✓	✓
1 + $BMes_3$ + D_2 + H_8-toluene	✓	✓	✓

This shows that hydrogenolysis of the nitride occurs because D as well as H are incorporated, but it shows that H/D exchange is occurring and that this is not from solvent since it is 'one-way' where only H gets incorporated into D-containing molecules not *vice versa*; the conclusion therefore is that solvent is not the source of this exchange so it must be from the TIPS groups in an amide/imido-cyclolometallate+H_2/D_2 equilibrium. We have ruled out the D_2 as the source of H because that is 99.8% atom D. We have further from these experiments been able to determined that when D_2 is the gas on average we get 64% ND_2, 24% NHD, and 12% NH_2; obviously when H_2 is used we get 100% NH_2 with no H/D scrambling.

Computational details

All calculations have been carried out with the Gaussian 09 program[10] at the DFT level of theory using the hybrid functional B3PW91.[11,12] Uranium atoms were described with a small-

core Stuttgart Dresden relativistic effective core potential (RECP), used in combinaison with its adapted basis set.[13-15] A 6-31(d,p) double – ζ quality basis set was used for hydrogen, boron, carbon and nitrogen atoms. Silicon atoms were treated with a small-core RECP with its optimised basis set to which was added a d polarisation function ($\alpha = 0.284$).[16,17] Electronic energies and enthalpies were computed at T = 298 K in the gas phase. Single point calculations including solvent effects with the SMD[18] methodology and dispersion corrections within the D3-BJ[19] approaches were carried out to draw the profiles. Geometry optimisations were performed without any symmetry constraints and analytical frequency calculations allowed to verify the nature of the extrema. Intrinsic Reaction Coordinates (IRC) were carried out to verify the connections of the optimised transition states. Natural Bond Orbital (NBO) analysis[20] was used to analyse electronic density.

Supplementary References

1. King, D. M., Tuna, F., McInnes, E. J. L., McMaster, J., Lewis, W., Blake, A. J. & Liddle, S. T. Synthesis and structure of a terminal uranium nitride complex. *Science* **337**, 717-720 (2012).
2. King, D. M., Tuna, F., McInnes, E. J. L., McMaster, J., Lewis, W., Blake, A. J. & Liddle, S. T. Isolation and characterisation of a uranium(VI)-nitride triple bond. *Nat. Chem.* **5**, 482-488 (2013).
3. Sheldrick, G. M. A short history of SHELX. *Acta Cryst. Sect. A* **64**, 112-122 (2008).
4. Sheldrick, G. M. SHELXT - Integrated space-group and crystal structure determination. *Acta Cryst. Sect. A* **71**, 3-8 (2015).
5. CrysAlisPRO version 39.46, Oxford Diffraction /Agilent Technologies UK Ltd, Yarnton, England.

6. Sheldrick, G. M. Crystal structure refinement with SHELXL. *Acta Cryst. Sect. C* **71**, 3-8 (2015).

7. Dolomanov, O. V., Bourhis, L. J., Gildea, R. J., Howard, J. A. K. & Puschmann, H. OLEX2: a complete structure solution, refinement and analysis program. *J. Appl. Cryst*. **42**, 339-341 (2009).

8. Farugia, L. J. WinGX and ORTEP for Windows: an update. *J. Appl. Cryst.* **45**, 849-854 (2012).

9. Persistence of Vision (TM) Raytracer, Persistence of Vision Pty. Ltd., Williamstown, Victoria, Australia.

10. Gaussian 09 Revision D.01, Frisch, M. J., Trucks, G. W., Schlegel, H. B., Scuseria, G. E., Robb, M. A., Cheeseman, J. R., Scalmani, G., Barone, V., Mennucci, B., Petersson, G. A., Nakatsuji, H., Caricato, M., Li, X., Hratchian, H. P., Izmaylov, A. F., Bloino, J., Zheng, G., Sonnenberg, J. L., Hada, M., Ehara, M., Toyota, K., Fukuda, R., Hasegawa, J., Ishida, M., Nakajima, T., Honda, Y., Kitao, O., Nakai, H., Vreven, T., Montgomery Jr., J. A., Peralta, J. E., Ogliaro, F., Bearpark, M., Heyd, J. J., Brothers, E., Kudin, K. N., Staroverov, V. N., Kobayashi, R., Normand, J., Raghavachari, K., Rendell, A., Burant, J. C., Iyengar, S. S., Tomasi, J., Cossi, M., Rega, N., Millam, J. M., Klene, M., Knox, J. E., Cross, J. B., Bakken, V., Adamo, C., Jaramillo, J., Gomperts, R., Stratmann, R. E., Yazyev, O., Austin, A. J., Cammi, R., Pomelli, C., Ochterski, J. W., Martin, R. L., Morokuma, K., Zakrzewski, V. G., Voth, G. A., Salvador, P., Dannenberg, J. J., Dapprich, S., Daniels, A. D., Farkas, O., Foresman, J. B., Ortiz, J. V., Cioslowski, J. & Fox, D. J. Gaussian, Inc.: Wallingford, CT, USA 2016.

11. Becke, A. D. Density-functional thermochemistry. III. The role of exact exchange. *J. Chem. Phys.* **98**, 5648-5652 (1993).

12. Burke, K., Perdew, J. P. & Wang, Y. In *Electronic Density Functional Theory: Recent Progress and New Directions*; Dobson, J. F., Vignale, G. & Das, M. P. Eds.; Plenum: New York, 1998.

13. Küchle, W., Dolg, M., Stoll, H. & Preuss, H. Energy-adjusted pseudopotentials for the actinides: Parameter sets and test calculations for thorium and thorium monoxide. *J. Chem. Phys.* **100**, 7535-7542 (1994).

14. Cao, X., Dolg, M. & Stoll, H. Valence basis sets for relativistic energy-consistent small-core actinide pseudopotentials. *J. Chem. Phys*. **118**, 487-496 (2003).

15. Cao, X. & Dolg, M. Segmented contraction scheme for small-core actinide pseudopotential basis sets. *J. Molec. Struct. (Theochem)* **673**, 203-209 (2004).

16. Bergner, A., Dolg, M., Küchle, W., Stoll, H. & Preuss, H. *Ab initio* energy-adjusted pseudopotentials for elements of groups 13-17. *Mol. Phys.* **80**, 1431-1441 (1993).

17. Höllwarth, A., Böhme, M., Dapprich, S., Ehlers, A. W., Gobbi, A., Jonas, V., Köhler, K. F., Stegmann, R., Veldkamp, A. & Frenking, G. A set of d-polarization functions for pseudo-potential basis sets of the main group elements AlBi and f-type polarization functions for Zn, Cd, Hg. *Chem. Phys. Lett.* **208**, 237-240 (1993).

18. Marenich, A. V., Cramer, C. J. & Truhlar, D. G. Universal solvation model based on solute electron density and on a continuum model of the solvent defined by the bulk dielectric constant and atomic surface tensions. *J. Phys. Chem. B* **113**, 6378-6396 (2009).

19. Grimme, S., Ehrlich, S. & Goerigk, L. Effect of the damping function in dispersion corrected density functional theory. *J. Comp. Chem.* **32**, 1456-1465 (2011).

20. Reed, E., Curtiss, L. A. & Weinhold, Intermolecular interactions from a natural bond orbital, donor-acceptor viewpoint. F. *Chem. Rev.* **88**, 899-926 (1988).
